# Protocol for a cluster-randomized control trial of a remote workplace resilience intervention for early care and education providers: The OnWARD trial

**DOI:** 10.1371/journal.pone.0340915

**Published:** 2026-03-13

**Authors:** Derek Hales, Regan Burney, Erik Willis, Emmy Clarke, Sarah Peterson, Shakiera Branch, Brandon Fowlin, Sherry Chesak, Lara A. Linnan, Amit Sood, Nazaret C. Suazo, Deborah J. Jones

**Affiliations:** 1 Center for Health Promotion and Disease Prevention, University of North Carolina at Chapel Hill, Chapel Hill, North Carolina, United States of America; 2 Center for Flourishing Health Care Communities, University of Minnesota, School of Nursing, Minneapolis, Minnesota, United State of America; 3 Department of Health Behavior, UNC Gillings School of Global Public Health, University of North Carolina at Chapel Hill, Chapel Hill, North Carolina, United States of America; 4 Sutardja Center for Entrepreneurship & Technology, U.C. Berkeley, Berkeley, California, United States of America; 5 Department of Psychology and Neuroscience, University of North Carolina at Chapel Hill, Chapel Hill, North Carolina, United States of America; PLOS: Public Library of Science, UNITED KINGDOM OF GREAT BRITAIN AND NORTHERN IRELAND

## Abstract

Work-related stressors take a toll on individuals’ health and well-being, a toll which is often heavier for under-resourced, low-paid segments of the essential workforce who serve our communities. Resilience programs have arisen as a promising workplace strategy to improve mental health and well-being. However, emerging programs are constrained by time and resource-intensive implementation strategies that are challenging to scale for marginalized segments of the workforce, including early childcare education (ECE) staff. The goal of this 15-month cluster randomized control trial is to compare change in resilience assets and resources for ECE staff in centers (n = 80 consisting of 640 ECE workers) randomly assigned to either a remotely delivered resilience (intervention) or physical activity (attention control) program. Measures will be collected at four timepoints: baseline (0 months), post-intervention (3 months), and long-term maintenance (9 and 15 months). Secondary outcomes will include changes in well-being, physical activity, organizational support, absenteeism, and turnover. Additionally, we will explore potential moderators of the treatment effects. The RE-AIM Framework will be used to determine the potential for individual (staff) and organizational (center) level reach, adoption, implementation, and maintenance of the two programs. Results will fill key gaps of prior resilience work by focusing on an underserved population in critical need of well-being resources with implications for the feasibility and impact of remote programming in other segments of the workforce.

Trial Registration: This trial is registered with the ClinicalTrials.gov registry (NCT06919952) and approved by the Institutional Review Board (IRB) at the University of North Carolina at Chapel Hill (IRB# 25−0016).

## Introduction

Work-related stressors impact individuals’ mental health and well-being, and contribute to long-term risks such as anxiety, depression, and burnout. Resilience training, aimed at enhancing the ability to maintain or regain mental well-being in the face of adversity [1], has received increased attention as a workplace strategy to support employee health [2,3]. Despite the clear need and potential benefits, there is a lack of scientifically rigorous, adequately powered, randomized controlled trials (RCTs) that assess the efficacy of resilience programs in occupational settings. The few RCTs to date have failed to include long-term maintenance outcomes or representative samples and still rely on resource intensive in-person treatment, which results in little evidence about their scalability, sustainability, and generalizability [1–7]. There is a critical need for rigorous, long-term RCTs to evaluate the efficacy and broader impact of feasible, fully remote resilience interventions, particularly those that can reach underserved and high-stress workforces, including those in early care and education (ECE) settings.

The ECE workforce is a large, essential, and historically underserved group that stands to benefit greatly from accessible, fully remote resilience programming. These professionals care for our nation’s young children and are consistently called upon by public health initiatives to support working families and promote children's physical, social, and emotional well-being; yet the well-being of ECE workers themselves has received limited attention. In recent years, surveys indicate 85% of childcare workers report feeling “more stressed” and 75% report feeling “exhausted or burnt out [8] have limited access to traditional wellness supports, are disproportionately low-income (average $13.00/hour) [9], uninsured (15.7% nationally) [10], and often navigate financial hardship (53% enrolled in public assistance programs) [11]. Remote delivery is thus critical for reaching this population, who often face rigid work schedules, multiple jobs, and transportation barriers. Focus groups conducted by our team found that nearly 70% of ECE workers expressed interest in participating in a remote program, underscoring both the need and relevance of this study. By centering this intervention on the long overlooked ECE workforce, this study advances resilience research in a population facing high distress, while also generating insights that may extend to other marginalized essential workers who face similar barriers and stressors (e.g., health aides, retail staff, janitors, laborers, food service).

The aim of this paper is to present the study protocol of a cluster randomized control trial designed to evaluate the efficacy of a fully remotely delivered, resilience intervention tailored for ECE staff and directors. The primary study hypothesis is that workers in ECE centers randomized to receive the resilience arm of the Online Wellness, Activity, and Resilience Development (OnWARD) program will demonstrate increased resilience scores relative to those in the physical activity arm of the intervention. Secondary outcomes will include changes in well-being, physical activity, organizational support, absenteeism/turnover, and potential moderators of the treatment effects. Process evaluation, fidelity checks, and ECE staff feedback will be used to document how the intervention is implemented and received at each center

## Materials and methods

The description of the study follows the Consolidated Standards of Reporting Trials (CONSORT) statement, with the extension for a cluster randomized control trial.

### Design overview

**Fig 1:**
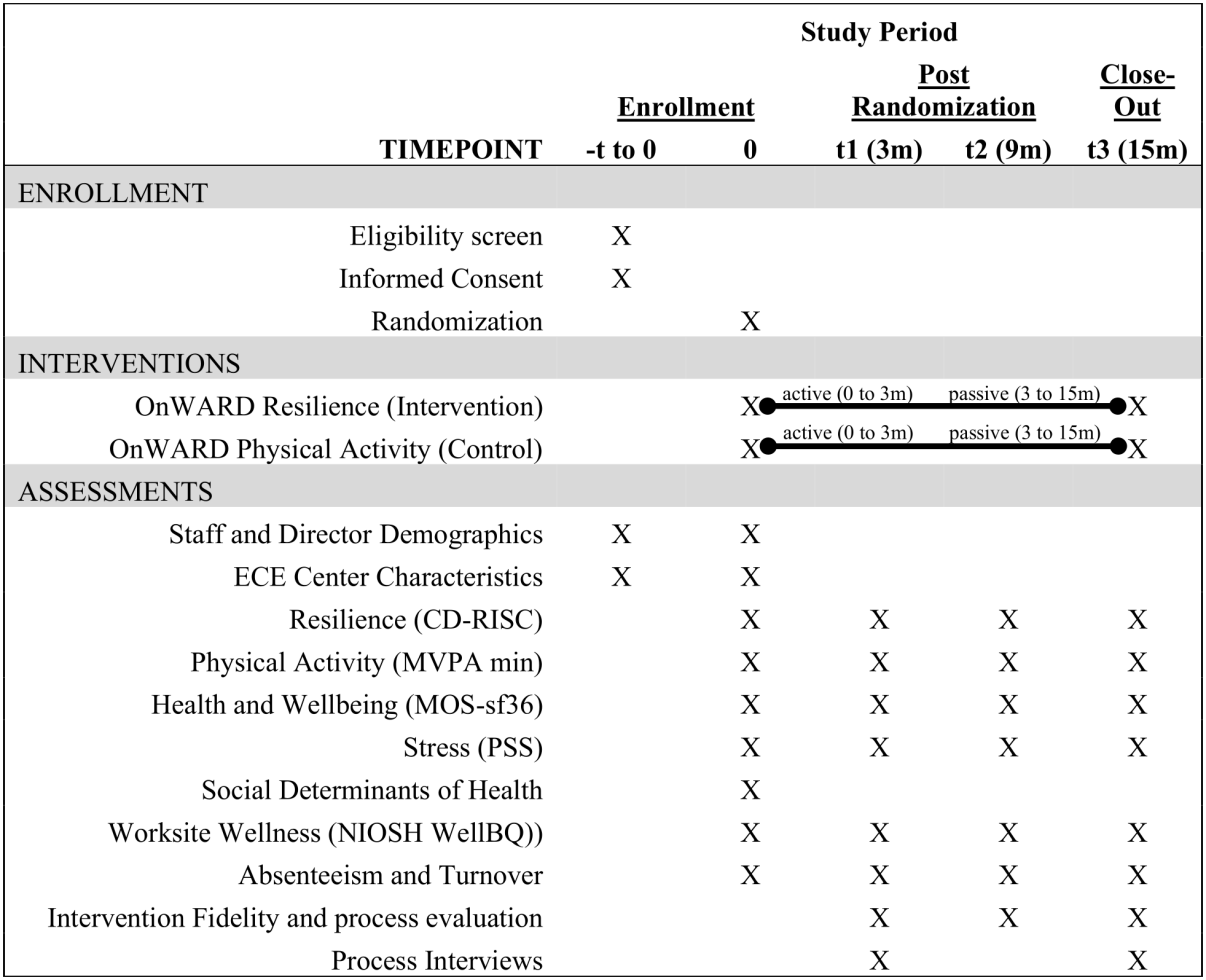
Required SPIRIT Participant Timeline: Schedule of enrollment, interventions, and assessments.

**Fig 2:**
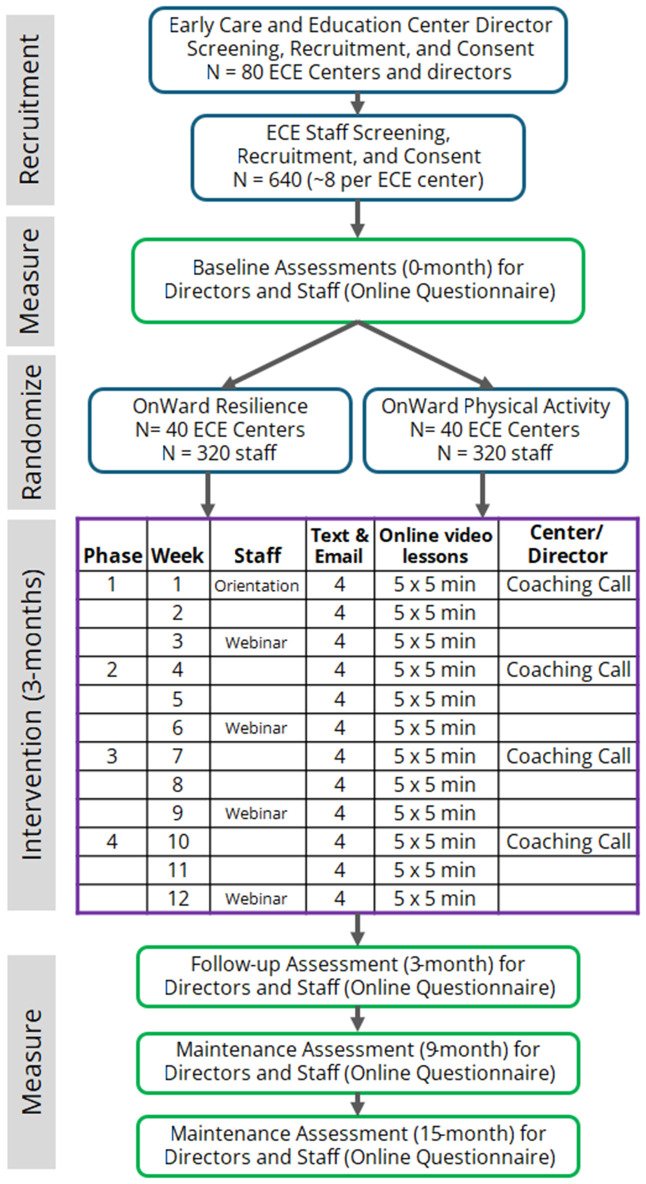
Study Design Overview.

This study is a cluster-randomized controlled trial with a three-month active intervention and 12-month maintenance period. The study sample will include 80 ECE centers and 640 ECE staff randomized (by center, 1:1) into either the resilience or physical activity arms of the intervention. Measures will be collected at baseline (0 months), post-intervention (3 months), and during the maintenance period (9 and 15 months). The primary outcome is change in ECE workers’ Connor-Davidson Resilience Scale scores between baseline and post-intervention (3 months). Secondary outcomes include changes in well-being, physical activity, organizational support, absenteeism, and turnover. Figs 1 and 2 display the required SPIRIT participant timeline and the study design flow overview. This study has been approved by the Institutional Review Board at the University of North Carolina-Chapel Hill (IRB# 25−0016) and registered at Clinicaltrials.gov (NCT06919952, Protocol version 1.0, 05 May 2025; https://clinicaltrials.gov/study/NCT06919952). Modifications to study protocol will be submitted to the IRB and Clinicaltrials.gov.

### Recruitment and setting

A convenience sample of 80 ECE centers and 640 staff (~8 per center including 1 director and 7 staff) will be recruited across four years in four cohorts from the southeastern United States. Recruitment began November 8^th^ 2025 and is expected to be complete in November 2027. Based on the intervention design and delivery, recruitment will be semi-rolling with the goal of starting 4–6 centers together on the same intervention timeline. Recruitment efforts will start by compiling a list of licensed ECE programs from publicly available state databases. From experience, we have had better success with cohorts recruited from a smaller regional area, allowing our team to identify community organizations which have established working relationships with local ECE centers. These community partners will be asked to endorse and promote participation and help distribute study information to center directors through their existing communication channels (e.g., newsletters, emails, partner website, group meetings). As described in Table 1, these communications will be followed by personalized email invitations from the study team with a brief study summary, invitation to participate, and a link to a short video containing additional information. Next, the team will follow up with phone calls to center directors to gauge interest and confirm center eligibility. Eligible ECE centers must 1) have at least 4 staff, 2) be licensed with no plans to close in the next 2 years, and 3) have been in operation for at least 1 year. Once initial interest and eligibility of the center is confirmed, the research team will work with the center director to engage and introduce the study to ECE staff, with the goal of consenting eight ECE staff. During this period, research staff will invite center staff to participate, send links to screening surveys and informational videos, answer questions about participation, and conduct informed consent procedures. Written consent will be obtained from each director and staff member through the digital survey platform. Eligible center staff must be 1) employed at a participating ECE center, 2) be at least 18 years old; 3) able to read and speak English; 4) have access to a device that can connect online (e.g., smartphone, tablet, computer); and 5) be willing to receive text messages. All recruitment, enrollment, and consent procedures will be conducted remotely.

**Table 1:** Schedule of Activities.

	Screening	BL Data Collection	Intervention (3mos)	3-month follow-up	9-month follow-up	15-month follow-up
**Recruitment and Baseline**						
Director: Welcome e-mail	X					
Director: Phone call and Screening	X					
Director: Consent and Online Survey		X				
Director: Follow-up about staff	X	X				
Staff: Online Screener	X					
Staff: Follow-up contact	X					
Staff: Consent and Online Survey		X				
Randomization			X			
**Intervention**						
Orientation			X			
Director: 4 Coaching Calls			X			
Staff: 3 Webinars			X			
Weekly Texts			X			
Adverse Event Reporting			X			
**Follow-up and Maintenance**						
Director: Follow-up contact				X	X	X
Director: Online Survey				X	X	X
Staff: Follow-up contact				X	X	X
Staff: Online Survey				X	X	X
Process Interview (12-Dir, 36 – staff)				X		X

*Note: All contact is remote/online*

### Randomization

Centers will serve as the unit of randomization. Soon after baseline measurement, each center will be randomized to either the OnWARD resilience or physical activity program. Randomization tables will be created by the data manager using a permuted block approach, with block sizes ranging from 2–4. The project manager will use this randomization table to assign centers to each arm (1:1).

### Blinding

Although it is not feasible to blind participants to group assignment in health behavior interventions, both groups in this study will be blinded to the primary research questions and hypotheses. Only the project manager and research staff who are helping to deliver the intervention will have access to the randomization table or be aware of intervention group assignments. Investigators, statisticians, and data collectors will remain blinded until follow-up data are unlocked. If the project manager, or DSMB, deem it necessary (e.g., SAE or substantial trial issue), a participant may be unblinded to investigators, the statistician and data collectors will be excluded from these conversations.

### Adverse events

Given the low-risk nature of the study and interventions, very few, if any, adverse events (AE) are expected. At enrollment, consent, and during orientation, we will inform participants to contact the project manager or interventionist if they believe they are experiencing a change in their mental or physical health may have occurred because of their participation in the study. In addition, every 4 weeks during the active intervention, we will ask via text: “Over the last month as a result of your participation in this study, have you had any physical or mental health problems that made it hard to do your normal daily activities or caused you to go to a doctor, counselor, or other healthcare provider? (Respond 1=Yes/2=No)”. If participants respond “no” or do not respond, no further steps will be taken. If participants respond “yes”, they will receive a follow-up text with a link to a short survey requesting more information about the issue and best contact times for a phone call follow-up. After follow-up, the project manager will send the IRB approved list of resources to the participant, as well as compile a detailed description of the event, the adverse outcome, severity, and whether participants viewed it as related to the study. This report will then be reviewed and assessed by the Data Safety and Monitoring Board (DSMB) (i.e., severity and relatedness) and the final AE report will be submitted to the IRB and funder when required. Given the minimal risk associated with this intervention study, it is highly unlikely that an accumulation of excess SAEs would prompt halting the trial. However, we will monitor SAE rates in all participants, with particular attention to those rated as severe and probably/definitely associated with study participation. If larger than expected SAE rates occur in either intervention the IRB and NCCIH will be alerted that halting or modifications are under review by the study team.

### Data safety and monitoring board

The three members of the DSMB have expertise in interventions, physical activity, mindfulness, statistics, and clinical psychology, but are not part of the study key personnel. No member of the committee has collaborated or co-published with the PIs within the past three years. The DSMB is tasked with reviewing each AE and determining severity and relatedness to participation in the study. In addition, they will review quarterly reports, including summaries of recruitment, AEs, withdrawal, and attrition. An annual report will also be compiled and address (1) whether AE rates are consistent with pre-study assumptions; (2) reasons for dropouts; (3) whether all participants met eligibility criteria; (4) whether continuation of the study is justified based on current enrollment and withdrawal rates; and (5) conditions whereby the study might be terminated prematurely (i.e., excessive SAEs, dropout over 50%, or substantial delivery issues [fidelity below 30%]).

### Intervention

**Fig 3:**
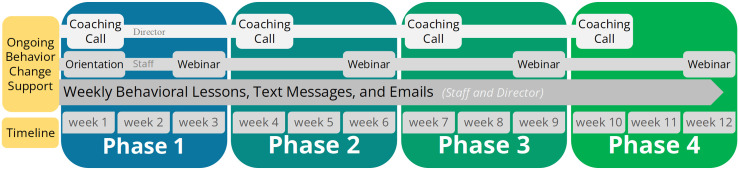
Outline of the Active Intervention Phases: Timeline and Components.

An outline of the intervention timeline and components are shown in Fig 3. Overall, both groups will participate in a 12-week fully remote delivered intervention focused on a particular aspect of their personal health and well-being (i.e., resilience/mindfulness or physical activity/sedentary). Over the 12 weeks, ECE staff will have access to weekly lessons, resources, webinars, and behavior prompts through text and email. ECE directors will also participate in coaching calls and develop worksite supports (organizational) for staff health. Following the 12-week active intervention phase (months 0–3), center directors and ECE staff will continue to have access to all study materials and resources for an additional 1 year no contact follow-up phase (months 4–15). Both groups will be encouraged to continue using the online resources and cycle through the improvement process, but weekly messages, webinars, coaching calls, and active interventionist support will stop. There are no restrictions on participant comcomitant care in this study and no study related intervention content or resources will be provided after the 1 year no contact follow-up.

### OnWARD resilience overview

The Stress Management and Resilience Training (SMART) will serve as the core component for the OnWARD resilience arm of the study. SMART offers an evidence-based, web-delivered resilience program that has shown initial efficacy regardless of the source of stressful thoughts or experiences. [1,2] The goal of SMART in this study is to improve the ECE workers’ mental health and well-being within the demands of their workplace. The program components will be delivered in four phases over 3-months (12-weeks) and implementation will be supported by trained research staff. The SMART program includes four modules: Gratitude, Mindful Presence, Kindness, and Resilient Mindset. As described elsewhere [12], the content of each module (four total) is designed to be covered in a group session, online training program with instructional videos, and a book [13]. The program also highlights and promotes daily skills practice and ongoing follow-up with module topics. Adaptations to the SMART program for this study are described below (*see Common Components).*

#### Rationale for use of SMART for resilience arm.

The SMART program builds upon principles and techniques of mindfulness, including attention training, with the goal of increasing intentional engagement with the present moment as an alternative to the more typical default of letting the mind wander [26–28]. Core strategies include joyful attention or delaying judgement, paying attention to novelty with the environment and people, kind attention, and practicing compassion for self and others in daily life. To this end, SMART includes a focus on interpretation refinement, or the practice of distinguishing between objective sensory information (e.g., sights, sounds) and our interpretations and judgements of that information, with the goal of approaching each day with less reactivity, more mindfulness, and enhanced resilience. SMART has produced promising results in several populations (e.g., teachers, nurses, physicians, family caregivers) through remote delivery [1,2,14,15].

### OnWARD physical activity (attention control) overview

Centers randomized to the attention control arm will receive a tailored ECE-based physical activity promotion program similar to those used by our team in previous trials [16–22]. Each staff member will have access to a private study specific website through Canva (account set up at orientation) where they can access materials that will support their adoption of evidence-based strategies for improving their own physical activity. Offering an alternative health promotion program affords an opportunity to assess the efficacy of the resilience arm compared to a program that has the potential to be an alternative therapy for stress and anxiety reduction for ECE workers. Like the OnWARD resilience arm, program components will be delivered in four phases over 3-months (12-weeks) with implementation supported by trained research staff. Timeline and logistical components will match the OnWARD resilience group (Fig 3). In the OnWARD physical activity arm, a progressive moderate-intensity exercise prescription will be utilized (i.e., walking, jogging, biking, etc.) as recommended by the Physical Activity Guidelines for Americans [23]. In addition, wrist-worn activity tracker will be provided (e.g., Amazfit, Fitbit) to help participants self-monitor their activity level in real time. The phone APP/website of the device will be used to view and keep track of activity minutes, steps, and progress. Other functions of these devices (i.e., sleep, weight, stress) are not the focus of this intervention, but process outcomes will be included to ask about uses of additional features of the devices.

#### Rationale for use of physical activity attention control.

Recently, there has been a call for resilience researchers to move beyond single-arm and wait-list control study designs to those that are randomized, controlled, and include matched attention comparison groups [3,4]. Although there is no current consensus on what type of attention control is best for resilience research [7], the use of physical health promotion interventions (e.g., physical activity, nutrition) have been shown to be feasible and acceptable [5,6]. The health benefits of increased physical activity are well established.

### Common intervention components

Both the OnWARD resilience and OnWARD physical activity groups will receive or be exposed to each of the following. Content will be group specific.

**Welcome package.** Each center will be mailed a “welcome box” at the start of the program. Boxes will provide directors and the ECE staff with intervention specific materials (e.g., workbooks [13], fitness trackers, wellness tools such as exercise bands and stress-relief items).

#### ECE worker orientation.

A 60-minute introductory, virtual group session for each ECE center will be led by the study interventionist. All participating ECE workers at a center will be encouraged to attend. Content will cover general components of the program, timelines, interacting and accessing study materials (e.g., website, book, journal), and a review of group specific components (e.g., activity tracker, daily practice) of the program. Based on past participant preference, this session will be offered twice to each center during the orientation week and will be scheduled after normal operating hours, or on the weekend, to maximize attendance. It will also be recorded and shared for staff unable to attend.

#### Behavioral Lessons.

Workers will have access to 12 online modules (~60-minute time commitment per module) which contain the core lessons and information for each program. The suggested pace during the intervention period is one module per week, but participants will have access to all modules from orientation until the end of the maintenance period and can complete or review lessons at their own pace. Table 2 contains an overview of the topics and content by phase, week, and group.

**Table 2:** Overview of Intervention Topics and Content by Phase, Week, and Group.

		OnWARD Resilience *(intervention)*		OnWARD Physical Activity *(control)*	
**Phase**	**Week**	**Topic**	**Example Content**	**Topic**	**Example Content**
1	1	Gratitude	Neurobiology of stress and the brain	Exercise 101	Setting SMART goals for physical activity
	2		Attention training through gratitude		Types of exercise and the benefits of exercise
	3		Resilience is the core strength to lift the load of life		Tracking activity or progress to encourage habit building
2	4	Mindful Presence	Intentional engagement with the present moment	Building a Routine	Practical ways to maintain motivation
	5		Curiosity in everyday tasks and interactions		Being active as a way of life
	6		Finding novelty in the familiar with people and things		Integrating activity into your workday
3	7	Kindness	Changing the brain's innate wiring	Exercise 201	Incorporating more variety into exercise routine
	8		Self-kindness and kind attention		Injury prevention and recovery
	9		Moving past tendencies of negativity and fear		Type, frequency, and intensity of exercise
4	10	Resilient Mindset	Interpretation refinement	Moving Forward	Re-evaluating goals over time
	11		Reinforcing a positive mindset		Planning ahead to maintain your routine
	12		Principles to live by Gratitude, Compassion, Acceptance, Meaning, Forgiveness		Relapse prevention

#### Director coaching calls.

We will provide training, consultation, and support via a 60-minute virtual one-on-one coaching call with center directors prior to each phase (Fig 3). Based on our worksite intervention experience [1,2,24–26], particularly with small businesses [27–31], these calls improve implementation efficacy. The goal of coaching calls is to support and encourage the director to work on organizational factors and environments that will enhance staff efforts to improve health behaviors as part of the intervention. Planned topics include ways to promote the relative benefits of the program with staff, strategies on how staff can use recommended practices within the workday, brainstorm ways to continue use of the program, techniques for identifying and informing staff members about positive changes, and referrals to the local, state, and national resources that may be helpful for long-term sustainability.

#### Weekly Messaging.

At the start of each week, a message will be sent to each participant with an introduction to the new module and a link to the lesson website. ECE staff will also receive 3 simple text messages each week. These messages are intended to: connect participants to content (intro to new lessons, highlight resources), motivate people to action (remind to watch, practice skill, try a suggestion from weekly lesson), and promote engagement through question and feedback. Messages will be sent by email and text unless a participant would prefer email only.

#### Webinars.

During the last week of each phase, participants will be invited to attend a 60-minute webinar led by the study interventionist. Webinars will be study group specific with staff from 2–3 centers invited to each. A webinar guide will be developed for each session to outline key information, example discussion questions, and potential activities to facilitate conversation and open discussion. Before each webinar ECE staff will be asked if they have questions or if there is anything they would like to discuss from the assigned modules. During the session, participants will be encouraged to talk about their progress and any concerns or challenges encountered. Based on past participant preference, this session will be offered twice during the week and will be scheduled after normal operating hours, or on the weekend, to maximize attendance. Sessions will be recorded and shared with any staff unable to attend.

#### Supplemental educational materials.

Participants will be mailed a resilience workbook [13] or physical activity promotion workbook [32] as part of their welcome package. These materials are designed to provide additional guided exploration of the program content and topics in the online modules. Other supplemental materials will be made available through study websites or email.

### Interventionist training and supervision

The Behavioral Health Counselor (BHC) will be trained to deliver both arms of the ONWARD intervention. For the SMART program, the BHC will complete the Trainer Skills Intensive program (~6 months), conducted Dr. Amit Sood (collaborator), to become a certified resilience trainer using the SMART program model. This standardized program provides the necessary knowledge and skills of the science and art of resilience. For the physical activity program, the BHC will receive standard training and certification which includes a mix of written materials, didactic sessions with the investigative team, and hands-on/practice-based exercises (~6 months). Dr. Hales (Co-PI) and Dr. Willis (Co-I) have extensive experience with physical activity interventions and will lead this training. Finally, the BHC will meet regularly with Dr. Jones (Co-PI) for general supervision related to their interaction with participants, including discussing the any challenges with the online delivery of program content, group dynamics in webinars, and/or participant responses to text messages.

### Theoretical model

**Fig 4:**
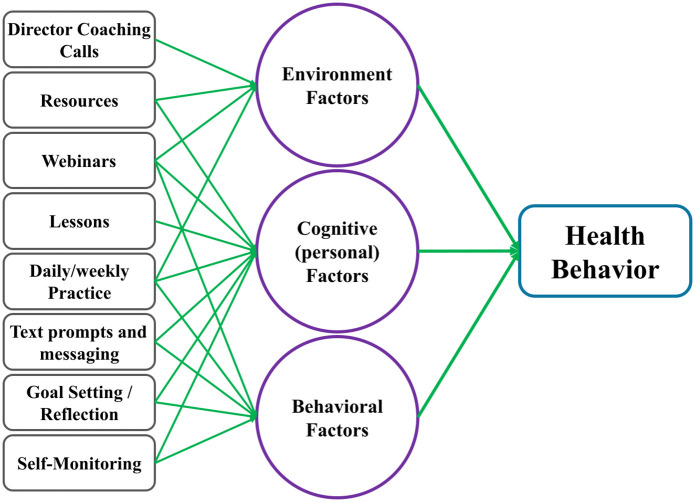
Simple Theoretical Model for Core Intervention Components.

The OnWARD intervention is informed by Social Cognitive Theory (SCT) [33] and incorporates evidence-based behavior change techniques aimed at improving adherence and promoting sustained engagement in both study arms, resilience and physical activity (attention control) [34–37]. Core strategies informing the structure and delivery include self-efficacy, observational learning, forming intentions, outcome expectations, worksite influences, knowledge building, cues to action, and self-regulation. With the goal of gradual behavior shaping, the intervention includes multiple reinforcing components. Webinars and short video lessons offer observational learning opportunities and knowledge-building, while text and email messages provide ongoing prompts and reinforcement. Goal setting, self-monitoring, and daily practice aim to support self-regulation and personal agency. The integration of coaching calls for center directors aims to further enhance the social and worksite support. A digital resource library offers staff practical tools to overcome barriers, build knowledge, and bolster expectations. By targeting both individual and organizational levels, the OnWARD program aims to influence childcare staff behaviors through enhanced personal factors, better quality behavioral techniques, and supportive environmental influences within the childcare setting (see Fig 4).

## Data management and sharing

### Data collection

Because the funding mechanism for this project requires fully remote recruitment, intervention delivery, and measurement (no in-person contact) all consent and data collection will occur through online systems (e.g., Qualtrics, REDCap). Some screening information and participant interviews will occur over the phone or on video conference (e.g., Zoom, Teams). Prior to baseline measurement, all participants will provide informed consent. Assessments will occur at baseline, 3 months (post-intervention), 9 months (maintenance), and 15 months (maintenance). During each assessment phase, participants will be informed (e.g., call, text, flyer to center) to expect a survey link (via email) during a specific week. If surveys are not completed after link is sent, a reminder will be emailed after 3 and 5 days. After 7 days, participants will be called and texted about survey completion. Surveys are designed to take 20–30 minutes and can be completed using any online connected device. Primary outcomes will be assessed at each time point, with demographics, secondary outcomes, and process/fidelity assessments administered as needed for the planned analysis. Additional details about measures are presented in the outcome measures section. Fifty participants (~12 per cohort) will be selected to be interviewed about their experience with their assigned program at the 3-month time point (post intervention). Data collection is expected to be completed February 2029.

Participants will receive incentives to complete different parts of the study. Once randomized, centers will receive a welcome box which contains items to welcome and support each participating staff member and the director. The welcome box for OnWARD resilience will include: a SMART book, a journal, a stress ball, an eye mask, and general instructions. Centers randomized to the physical activity arm will receive activity tracking devices (e.g., Fitbit), a journal, water bottle, a stretchy band, and general instructions. Consented and enrolled staff members at a center will receive one of each item in the welcome box. Centers, through the director, will receive $100 in gift cards for participating at baseline and again at 3 months (total = $200). They will receive these incentives after all consented staff have completed their online survey for the relevant timepoint. Participating ECE staff will receive a $50 TANGO gift card after completing surveys at each data collection timepoint (total of $200 over 4 time points [BL, 3m, 9m, 15m]). After completing the 3-month active intervention period, a random sample of participants will be chosen to participate in structured feedback and process interviews. Each participant will receive $25 (added to gift card) once they complete the interview. Participants in both arms can also receive up to 8 contact hour continuing education credits during the 3 months active intervention. These hours can be applied to the state-level requirements for on-going training that providers must meet each year. For both groups, contact hours will be earned in 2-hour segments through watching/completing the videos and lessons related to each module (4 modules = 8 total contact hours possible) and completing a required feedback and reflection where they answer a few questions about the relevant module. After module completion is confirmed and the feedback and reflection are completed by the participant, a certificate will be emailed or mailed (by request).

### Data management

Data management and processing will involve a collaborative effort between the project and data managers with guidance and feedback from the statistical team. Several data streams will be established and maintained throughout the project. During recruitment and screening the project manager and staff will maintain a contact and tracking database allowing for a streamlined center/participant engagement process. During measurement periods all surveys will be administered and collected through the Qualtrics online system. Separate surveys will be administered to directors and staff. Surveys will be distributed using participant specific links and programed with appropriate data validation checks, response requirements, and several “data quality check” items. The project and data managers will work together to monitor survey completion and conduct initial data quality assessments (i.e., missing responses, straight line responding, survey completion times). If a participant is missing needed items, they will be contacted to collect this information (e.g., skipped CD-RISC). Data cleaning and scoring will follow the established protocol for all surveys selected (e.g., CD-RISC). The project manager will lead the compilation of process data, using it to create implementation reports that are reviewed regularly during biweekly meetings. Website/lesson engagement data and attendance/call tracking information will be maintained by interventionist and project manager. The quality of data collected via online surveys will be regularly reviewed by the data manager to check for completeness. The project manager will be engaged immediately if follow-up is needed to address any errors or missing data. After each measurement period, the project manager and data manager will clean and manage the data (e.g., apply data labels, examine distributions, review data for extreme values, create and check derived variables, create and save datasets, and develop a data user manual) under the direction and guidance of the study statistician. The project manager and data manager will report on the progress of their activities and issues that need to be addressed (e.g., adverse event, non-response) during biweekly meetings. All data are stored on servers maintained by the university with 2-factor authentication and VPN requirements for staff access.

### Data sharing

Public use study data and associated documentation will be deposited in the Research Data Management Core’s UNC Dataverse. Dataverse is a digital archive for scholarly materials produced by members of the University of North Carolina at Chapel Hill community. Dataverse provides searchable study-level metadata for dataset discovery and has a robust preservation plan to ensure long-term access. Upon conclusion of data collection for this study, all direct respondent identifiers (e.g., names and addresses) will be expunged and securely maintained in a separate control file. Deidentification will be finalized by the end of data processing. The cleaned data used to assess study aims will be made publicly available no later than the time of publication of the primary results. Public data maintained in the Dataverse will be discoverable online. The duration of preservation and sharing of the data will be a minimum of 10 years after the funding period. There are no anticipated factors or limitations that will affect the access, distribution, or reuse of the survey data generated by the proposal.

### Objectives and outcome measures

Table 3 presents a description of the study objectives and outcomes measures. Outcomes are measured at baseline, post active intervention (3 months), and twice during the no contact intervention maintenance phase (9- and 15-months) and in both treatment and control groups. Data to assess objectives is collected from both ECE directors and staff enrolled in the study. To comply with the fully remote mandate for the funding mechanism, all primary and secondary outcome measures are collected using online survey systems.

**Table 3:** Summary of Outcome Measures and Associated Objectives.

Outcome	Level	Who	When	Instrument	Objective(s)
Demographics	IP	S/D	0,3	Study specific	Descriptive Exploratory- Moderating effects
Resilience	IP	S/D	0,3,9,15	Connor-Davison Resilience Scale (CD-RISC)	Primary – change BL to 3m Secondary – change 3m to 9m, change 9m to 15m
Health and well being	IP	S	0,3,9,15	Medical Outcomes Study 36-Item Short-Form Health Survey (MOSsf36)	Secondary – change BL to 3m
Stress	IP	S	0,3,9,15	Perceived Stress Scale (PSS)	Secondary – change BL to 3m
Readiness for action	IP	D/S	0	Study specific	Exploratory- moderating effects
Social Determinants of Health	IP	S	0	adapted from AHC Health-Related Social Needs screening tool and AAFP Social Needs screening tool	Exploratory- moderating effects
Center Descriptives	Org	D	0,3	Study specific	Descriptive Exploratory- moderating effects
Worksite Wellness	Org	D/S	0,3,9,15	NIOSH worker well-being questionnaire (WellBQ).	Secondary – change BL to 3m
Absenteeism and Staff Turnover	Org	D/S	0,3,9,15	adapted from Work Limitations Ques., Work and Health interview, WHO health and work performance Ques.	Secondary – change BL to 3m
Intervention Fidelity and Process Evaluation	IP/Org	D/S	3,9,15	study specific questions and interviews guided by RE-AIM	Secondary – differences in engagement Exploratory- moderating effects

NOTE:IP = Intrapersonal, Org = Organizational, BL = Baseline, m = months, S = Staff, D = Director

### Objectives

The primary aim of the study is to compare change in resilience scores from baseline to 3-months between the invention (OnWARD resilience) and control groups (OnWARD physical activity). Secondary and exploratory objectives are highlighted in Table 3.

### Outcome measures

All measures will be collected from ECE staff and directors using the Qualtrics online survey system. Personal survey links will be sent with reminders. Question order and surveys will be randomized when appropriate.

#### Resilience.

The primary outcome will be assessed with the Connor-Davison Resilience Scale (CD-RISC), a 25-item self-administered questionnaire that captures personal qualities that support one’s ability to bounce back in the face of stress and adversity [38]. It is the most commonly used assessment of resilience in research [3]. Item are rated on a 5-point Likert-type scale with a total score ranging from 0 to 100. Internal consistency is good (Cronbach’s alpha = 0.89) and scores are negatively correlated with stress (*r* = −076, p < 0.001) [38] and have been shown to be sensitive to change in several studies [1,2,39].

#### Health and Well Being.

The Medical Outcomes Study 36-Item Short-Form Health Survey (MOSsf36) will be administered to assess overall physical and mental health quality. The survey captures a broad spectrum of physical and psychological distress which can be summarized into component scores. The MOSsf36 has strong psychometric properties and widespread cross-cultural implementation [40,41,42,43]. The Mental Health Inventory-5 (MHI-5), a subscale of the MOSsf36, will be used to specifically measure psychological well-being and distress. The brief 5-item scale assesses positive affect and symptoms of depression and anxiety. The MHI-5 has strong test-retest reliability and sound internal consistency and compares well with other measures of mental health [44–46].

#### Stress.

The Perceived Stress Scale (PSS) is a self-report assessment of life stress and control over common stressors [47]. The 10-items of the PSS ask participants to rate the frequency of stress related events over the last month on a 5-point (never to very often) likert-type scale. Items have been shown to have strong internal consistency (Cronbach’s Alpha = 0.75–0.89) with scale scores also demonstrating good reliability/validity evidence as well as its sensitivity to change in intervention studies [47–49]. The scale is extensively used in diverse populations.

#### Worksite Wellness.

Organizational support and resources will be assessed using the National Institute for Occupational Safety and Health (NIOSH) worker well-being questionnaire (WellBQ). The WellBQ is a comprehensive instrument designed to assess occupational and non-occupational influences on health [50,51]. Thirty items from the ‘work evaluation” and “workplace policies and culture” sections will be included. Items are generally rated on a 4- or 5-point Likert-type scale assessing satisfaction, frequency or level of agreement with a statement. Subcomponent scores are computed on a 0–100 scale. Psychometric testing has demonstrated strong internal consistency across domains and evidence of construct validity through expected associations and factor structure [50,52].

#### Absenteeism and Staff Turnover.

Measures of productivity losses were adapted from common survey questions used to assess missed work, turnover, and hiring impacts on organizational function [53–56]. Staff absenteeism will be assessed by asking enrolled staff: “In the last 4 weeks, how many days have you…”: “missed an entire shift, or workday”, “...missed part of a shift or part of a workday”, and “...missed 15 to 60 minutes because you were late to work”. In addition, ECE directors will be asked “On average, how many days per month does at least one classroom staff member miss a full day, partial day, or are late. Items will be used individually and in combination to estimate rates of absenteeism and average days per month staff missed work. Staff turnover and hiring will be assessed by asking directors about the number of open positions, hiring in the last 3-months, the number of staff who have left, or resigned, by choice, and the number of staff who were dismissed or fired. This information will be used to compute rates of position vacancies and voluntary/involuntary turn-over (i.e., involuntary turnover rate = 100 x [# staff fired/# total employees]).

#### Readiness for action.

To measure participant readiness for behavior change, a study specific survey with questions related to intervention actions was developed. Each of the 20-items asks: “Over the next 3 months, how likely are you to...[*behavior/action that is part of one or both intervention arms*]” (e.g., …track your daily health habits”, “...practice 2-minutes of daily gratitude.”). Items are rated on a 7-point scale ranging from “will not do” to “very likely”. In order to estimate intervention specific readiness 10 items related to general intervention targets, 5 were specific to the OnWARD resilience arm, and 5 were actions related to the physical activity group (attention control).

#### Social Determinants of Health.

Based on the accepted components of the social determinants of health framework (e.g., Healthy People 2030, CDC, WHO), we will assess economic stability, built environment, social cohesion, community context, food insecurity, education; and health care access [57,58]. Survey items are adapted from two widely recognized tools: the Accountable Health Communities Health-Related Social Needs screening tool developed by the Centers for Medicare & Medicaid Services and the American Academy of Family Physicians Social Needs Screening Tool [59,60]. Incorporating these measures provides critical insight into the contextual factors that shape health outcomes beyond individual clinical or behavioral characteristics [61–64].

#### Personal Demographics and Center Descriptives.

Descriptive information will be collected for the ECE centers, including quality rating, participation in Child and Adult Care Food Program, and numbers, ages, and race/ethnicity of children enrolled. For the childcare workers (directors and staff) we will collect age, race/ethnicity, marital status, income, and size of household.

**Additional health behaviors.** Surveys also include simple assessments of common health related behaviors including sleep, diet, physical activity/exercise, self-care, meditation, and mindfulness activities.

#### Intervention fidelity and process evaluation.

The process evaluation will be guided by Steckler and Linnan [65] and the RE-AIM model framework [16,66]. We will measure reach (% who participate in each intervention component, engagement with website and worker representativeness); *effectiveness* (assessed via aims); *adoption* (center level measure of uptake & representativeness); *implementation* (dose delivered; adherence to quality and fidelity of established protocols); and *maintenance* (worker and center level durability of effects 12-months post-intervention) [66]. Our previous process evaluation work also includes measuring *recruitment* (# approached, # eligible, # enrolled and costs per center & workers enrolled), and *responsiveness* (worker satisfaction and use/engagement with intervention components). Surveys administered at 3-, 9-, and 15-months will include satisfaction, engagement (initial, continued, and timing), and feedback questions related to the intervention components for the assigned group. Website use will also be summarized using website platform tracking metrics (logins per month, # lessons viewed) at 3-,9-, and 15-months. The interventionist and project manager will also maintain attendance and scheduling attempt records and text delivery/response rates to quantify intervention engagement.

All orientations, coaching calls, and webinars will be recorded; 10% will be rated by 2 independent raters using a fidelity checklist. Fidelity raters will be blinded to participant and center names involved in each session using audio and transcript materials that have identifying information removed by the interventionist; however, the intervention content discussed during a session makes it impossible to completely blind raters to study arm. Data from raters will be used to assess competence and adherence to content standards. The automated nature of the lesson materials and feedback messages in this study ensure fidelity of core intervention content following orientation but will be routinely monitored by the research team to ensure protocol compliance.

More detailed feedback will be obtained through structured interviews with 12 directors and 36 staff (balanced over cohorts). Interviews will be conducted by phone or video conference in a random sample of participants across two strata (race/ethnicity and program engagement) at 3- and 15-months to gather qualitative information that might be useful in improving the intervention. Topics will include preference for intervention format, length, barriers to intervention components, suggestions to improve the intervention, overall satisfaction, including all the online trainings [67].

## Data analysis

Data analyses will be performed using SAS statistical software (Version 9.4 or higher, Cary, NC). Baseline descriptive characteristics will be summarized globally, and by arm, using frequencies and percentages for categorical variables, and means and standard deviations (or median [Q1, Q3] for non-symmetric data) for continuous variables. Prior to conducting data analyses, we will audit the data for completeness and quality, including missing data. We will evaluate distributions to ensure that they meet the assumptions of planned analyses, including the detection of outliers. Study result are expected to be released August 2029.

### Primary outcomes

Primary analyses will be performed using data from all randomized participants (ITT population). Additional assessments of primary endpoints will be completed for study completers (baseline and follow-up assessments) and on participants completing at least 50% of the assigned intervention tasks (i.e., lessons and webinars). Our primary analyses will involve testing change in the total score from CD-RISC between OnWARD resilience and physical activity arms at 3 months. Using maximum likelihood methods, we will use multi-level linear mixed models (PROC MIXED) that include random effect for cluster to account for covariance between participants within the same center as well as fixed effects for time (0, 3, 9, 15 months), trial arm (OnWARD resilience or physical activity), time arm interaction. To assess the primary endpoint from this mixed model we will include a contrast statement to calculate mean and 95% confidence intervals to estimate change for our primary outcome from baseline to 3- (primary endpoint), 9-, and 15-months (secondary endpoints). A proper error covariance structure will be chosen based on model fit indicated by model likelihood, Akaike Information Criterion and Bayesian Information Criterion. Residuals will be examined to check the assumptions of models. To further explore the effect of the intervention, sensitivity analyses will be conducted that adjust for baseline variables distributed differently between groups and to examine completers only.

### Secondary and exploratory outcomes

Analysis of secondary end points will follow the same procedures as outlined for our primary analyses. Similar statistical analyses will be completed for these outcomes.

### Covariates

While randomization should be sufficient to balance baseline characteristics of the staff and directors recruited to each arm, we will compare age, race/ethnicity, weight, income, education, physical activity level, years working at ECE center, center size, and center cost (per week) between study arms using simple mean or frequency comparisons. If group differences are deemed statistically significant and clinically meaningful, we will fit models with and without covariates for assessment. Baseline variables including center and worker demographics will be included as covariates to assess their potential moderating effect

### Missingness

All analyses will be conducted using the intention-to-treat principle, in which all available data on all randomized participants are included. This approach minimizes bias if individuals drop out of the trial for different reasons. Every effort will be made to obtain follow-up data on all participants randomized, whether they follow their assigned treatment. Our maximum likelihood approach assumes that any missing outcome data are missing at random (i.e., missing data including that due to drop-out can be dependent on any previously observed outcomes or treatment assignment) [68]. With this approach, we use all data that have been collected without regard to whether data are missing for a participant at another visit, including drop-out, and without explicit imputation of missing data. If the combined rate of missingness on variables is above 5%, we will impute missing endpoint data using multiple imputation techniques [69] and will assess the sensitivity of our results to various assumptions of missing data patterns [70]. The methodological literature currently recommends an inclusive analysis strategy that incorporates auxiliary variables into the missing data handling procedures because this approach can make the missing at random assumption more plausible and can improve statistical power [71, 72]. All outcome variables and auxiliary variables (i.e., measured worker and center characteristics) will be incorporated into the imputation process.

### Power calculations

The study is powered to detect a between group difference (Resilience group vs attention control) of overall resilience assets and resources as assessed with the CD-RISC (primary outcome). An effect size of at least 0.35 is expected, representing a clinical meaningful difference and a conservative estimate based off the well-documented efficacy of the SMART program upon which the OnWARD resilience intervention is based (with effect sizes ranging from 0.3 to 1.1). Based on previous work we anticipate that, on average, about 8 workers will participate at each childcare center. Since this is a cluster randomized design, we assume that there will be an inherent intraclass correlation coefficient (ICC) within centers. The ICCs observed in our previous work assessing childcare worker health outcomes ranged from 0.00 to 0.13.

Table 4 describes the power for a range of ICCs, number of centers per arm, and effect sizes. Given these assumptions, a sample size of 80 centers (40 per arm), with an average of 8 workers per center, and an ICC ~ 0.10 would allow us to detect an effect size of 0.35 with 90% power at α = 0.05 level of significance. Even with a 15% loss to follow-up at 3 months (e.g., 12 centers or 96 workers), which is higher attrition than we expect based on our previous childcare-based trials, a completer’s only analysis will still provide 84% power. An important secondary aim is to compare the maintenance of resilience interventions from 3 months to 15 months. Anticipating higher loss to follow-up at 15 months and assuming a minimal detectable effect size of at least 0.35, we retain 82% power with an ICC = 0.10 with 20% attrition (e.g., 16 centers or 128 workers). Missing data will be fully recovered, which will remove or minimize (if present) confounding effects of missingness on our statistical power.

**Table 4:** Estimates of Statistical Power Considering Sample Size, Effect Size, and ICC.

Centers per arm	Effect Size	ICC			
		0.00	0.03	0.10	0.13
**30**	**0.30**	0.91	0.84	0.67	0.60
**30**	**0.35**	0.97	0.93	0.80	0.73
**30**	**0.40**	0.99	0.98	0.89	0.84
**35**	**0.30**	0.94	0.89	0.73	0.67
**35**	**0.35**	0.99	0.96	0.85	0.80
**35**	**0.40**	1.00	0.99	0.93	0.89
**40**	**0.30**	0.97	0.92	0.79	0.73
**40**	**0.35**	0.99	0.98	**0.90**	0.85
**40**	**0.40**	1.00	0.99	0.96	0.93

## Discussion

This paper presents the protocol for a cluster-randomized control intervention trial aiming to improve resilience among staff in ECE settings. The fully remote intervention aims to provide an accessible and scalable program for this at-risk and underserved group with implications for other essential and full-time segments of the workforce. The research team’s history of collaboration with staff in early childcare settings affords a unique opportunity to extend the science and methodology of workplace resilience interventions generally toward a potentially more sustainable model for promoting the health and well-being of workforces like childcare. ECE workers in particular, despite the importance of their work, are paid low wages, are un- or underinsured, experience high levels of stress and, in turn, experience high levels of burnout resulting in high levels of turnover in ECE settings with significant detrimental effects on childcare workers themselves as well the children and families they serve. As such, the results of this study will further our scientific understanding of workplace resilience interventions and their cascading effects for absenteeism and turn-over, while also learning more about the feasibility and scalability of such approaches.

### Limitations

Like all research, the proposed study design and methods have potential challenges that must be anticipated. First, recruiting both center directors and staff remotely will be complicated and challenging. However, our team has a long history of successful recruitment for ECE based RCTs and many strong community partners that can facilitate introductions and encourage participation. We also know from experience that diligent follow-up is essential, including both phone and email. The recruitment strategies build on experience from our past studies used to successfully recruit over 6,000 childcare programs and 1,100 workers [11,21,73]. If recruitment, withdrawals, or loss to follow-up becomes a concern we will engage with our network of community and research partners outside of southeastern U.S. Through our Go NAPSACC program [74–76], we have partners in 29 states and have been successfully enrolling and collecting data remotely for several years. Beyond recruitment, we also acknowledge that our outcome measures are self-report. Given the fully remote mandate by the funding agency, self-report was the only feasible option. That said, we have tried to select measures with strong evidence for reliability, validity, and ability to detect change. Survey design and incentives should also help alleviate burden and promote completion. Given the fully remote nature of the study, reliable internet is needed to access and engage with nearly all materials and meetings. With 99% of 18- to 64-year-olds using the internet and more than 80% having home broadband access, we do not anticipate access limiting participation [77]. If someone has internet issues and would like to participate, we will try to help them identify options for Wi-Fi access (e.g., work, library, etc.).

### Strengths

We are an experienced, multi-disciplinary research team with considerable expertise in design, implementation, and analysis of effective, digital interventions conducted in both childcare and workplace settings. In addition, our process evaluation will provide the opportunity to clarify why, how, and for whom the OnWARD resilience intervention is most effective. Third, the use of attention match control group affords confidence that any treatment gains in resilience are a function of the intervention, which has not been the case in prior resilience intervention research. In addition, using a physical activity program for comparison strengthens confidence in independent vs complementary effects. Fourth, we focus on childcare workers who are essential workers at elevated risk given they are often under-resourced and experience high levels of stress resulting in burn-out and turnover. Fifth, we include measures capturing interpersonal and organizational level outcomes, which will help us to assess the real-world scalability of this approach. Finally, our assessment of demographic and social determinants of health as potential moderators of the intervention will provide insight into the factors which impact intervention effectiveness.

### Impact of results

The project’s fully remote delivery and integrative approach has the potential to increase the reach and accessibility of resilience programs for the 1.15 million childcare workers and other essential and full-time workers in the United States. Findings will provide guidance for a scientifically sound, replicable therapeutic program with potential for rapid translation, implementation, and sustainability with implications for other critical, yet marginalized segments of the workforce.

## Supporting information

S1 File Original Protocol Submitted and Accepted by Funding Agency. (PDF)

S2 File Survey Questions Used to Measure Primary and Secondary Outcomes. (PDF)
