# Correction: Protocol for a cluster-randomized control trial of a remote workplace resilience intervention for early care and education providers: The OnWARD trial

**DOI:** 10.1371/journal.pone.0347833

**Published:** 2026-04-21

**Authors:** Derek Hales, Regan Burney, Erik Willis, Emmy Clarke, Sarah Peterson, Shakiera Branch, Brandon Fowlin, Sherry Chesak, Laura A. Linnan, Amit Sood, Nazaret C. Suazo, Deborah J. Jones

The ninth author’s name is spelled incorrectly. The correct name is: Laura A. Linnan.

The ORCID iD is missing for the ninth author. Please see the author’s ORCID iD here:

- Author Laura A. Linnan’s ORCID iD is: 0000-0002-8702-4624 (https://orcid.org/0000-0002-8702-4624).
