# Supplementary material for: Protocol for a cluster-randomized control trial of a remote workplace resilience intervention for early care and education providers: The OnWARD trial
Source: PLoS One. 2026 Mar 13;21(3):e0340915. doi: 10.1371/journal.pone.0340915 (PMC12987477; doi:10.1371/journal.pone.0340915)
Supplement: S1 File — (PDF) [file pone.0340915.s001.pdf]

OnWARD: Comparing a Workplace Resilience and a Physical Activity Intervention on Early Childhood Educators Well-being

Protocol Number*: IRB# 25-0016

National Clinical Trial (NCT) Identified Number: NCT06919952

Principal Investigator*: MPI Dr. Deborah Jones and Dr. Derek Hales

Sponsor: University of North Carolina at Chapel Hill

Grant Title: A Cluster-Randomized Control Trial of a Workplace Resilience Intervention for Child Care Providers' Mental Health & Well-Being

Grant Number*: R01AT012620

Funded by: National Center for Complementary and Integrative Health

Version Number: v.1.0

2025-05-06

CONFIDENTIALITY STATEMENT
[Original statement removed per PLOS Editorial Policy]

Protocol is published under the Creative Commons Attribution (CC BY) 4.0 license. Please include proper attribution with any use of these materials.

Table of Contents

STATEMENT OF COMPLIANCE 1
INVESTIGATOR'S SIGNATURE 2
1 PROTOCOL SUMMARY 3
1.1 Synopsis 3
1.2 Schema 5
1.3 Schedule of Activities 6
2 INTRODUCTION 7
2.1 Study Rationale 7
2.2 Background 7
2.3 Risk/Benefit Assessment 8
2.3.1 Known Potential Risks 8
2.3.2 Known Potential Benefits 8
2.3.3 Assessment of Potential Risks and Benefits 9
3 OBJECTIVES AND ENDPOINTS 11
4 STUDY DESIGN 13
4.1 Overall Design 13
4.2 Scientific Rationale for Study Design 14
4.3 Justification for Intervention 14
4.4 End-of-Study Definition 14
5 STUDY POPULATION 15
5.1 Inclusion Criteria 15
5.2 Exclusion Criteria 15
5.3 Lifestyle Considerations 15
5.4 Screen Failures 15
5.5 Strategies for Recruitment and Retention 15
6 STUDY INTERVENTION(S) OR EXPERIMENTAL MANIPULATION(S) 17
6.1 Study Intervention(s) or Experimental Manipulation(s) Administration 17
6.1.1 Study Intervention or Experimental Manipulation Description 17
6.1.2 Administration and/or Dosing 17
6.2 Fidelity 18
6.2.1 Interventionist Training and Tracking 18
6.3 Measures to Minimize Bias: Randomization and Blinding 18
6.4 Study Intervention/Experimental Manipulation Adherence 18
6.5 Concomitant Therapy 19
6.5.1 Rescue Therapy 19
7 STUDY INTERVENTION/EXPERIMENTAL MANIPULATION DISCONTINUATION AND PARTICIPANT DISCONTINUATION/WITHDRAWAL 20
7.1 Discontinuation of Study Intervention/Experimental Manipulation 20
7.2 Participant Discontinuation/Withdrawal from the Study 20
7.3 Lost to Follow-Up 20
8 STUDY ASSESSMENTS AND PROCEDURES 21
8.1 Endpoint and Other Non-Safety Assessments 21
8.2 Safety Assessments 21
8.3 Adverse Events and Serious Adverse Events 22
8.3.1 Definition of Adverse Events 22
8.3.2 Definition of Serious Adverse Events 22
8.3.3 Classification of an Adverse Event 23

8.3.4 Time Period and Frequency for Event Assessment and Follow-Up 23
8.3.5 Adverse Event Reporting 24
8.3.6 Serious Adverse Event Reporting 24
8.3.7 Reporting Events to Participants 25
8.3.8 Events of Special Interest 25
8.3.9 Reporting of Pregnancy 25
8.4 Unanticipated Problems 25
8.4.1 Definition of Unanticipated Problems 25
8.4.2 Unanticipated Problems Reporting 26
8.4.3 Reporting Unanticipated Problems to Participants 26
9 STATISTICAL CONSIDERATIONS 27
9.1 Statistical Hypotheses 27
9.2 Sample Size Determination 27
9.3 Populations for Analyses 28
9.4 Statistical Analyses 28
9.4.1 General Approach 28
9.4.2 Analysis of the Primary Endpoint(s) 28
9.4.3 Analysis of the Secondary Endpoint(s) 29
9.4.4 Safety Analyses 29
9.4.5 Baseline Descriptive Statistics 29
9.4.6 Planned Interim Analyses 29
9.4.7 Sub-Group Analyses 29
9.4.8 Tabulation of Individual Participant Data 29
9.4.9 Exploratory Analyses 29
10 SUPPORTING DOCUMENTATION AND OPERATIONAL CONSIDERATIONS 31
10.1 Regulatory, Ethical, and Study Oversight Considerations 31
10.1.1 Informed Consent Process 31
10.1.2 Study Discontinuation and Closure 31
10.1.3 Confidentiality and Privacy 31
10.1.4 Future Use of Stored Specimens and Data 32
10.1.5 Key Roles and Study Governance 32
10.1.6 Safety Oversight 32
10.1.7 Clinical Monitoring 33
10.1.8 Quality Assurance and Quality Control 33
10.1.9 Data Handling and Record Keeping 35
10.1.10 Protocol Deviations 35
10.1.11 Publication and Data Sharing Policy 36
10.1.12 Conflict of Interest Policy 36
10.2 Additional Considerations 36
10.3 Abbreviations and Special Terms 36
10.4 Protocol Amendment History 38
11 REFERENCES 39

STATEMENT OF COMPLIANCE

The trial will be carried out in accordance with International Council on Harmonisation Good Clinical Practice (ICH GCP) and the following:

- United States (US) Code of Federal Regulations (CFR) applicable to clinical studies (45 CFR Part 46, 21 CFR Part 50, 21 CFR Part 56, 21 CFR Part 312, and/or 21 CFR Part 812).

National Institutes of Health (NIH)-funded investigators and clinical trial site staff who are responsible for the conduct, management, or oversight of NIH-funded clinical trials have completed Human Subjects Protection and ICH GCP Training.

The protocol, informed consent form(s), recruitment materials, and all participant materials will be submitted to the IRB for review and approval. Approval of both the protocol and the consent form(s) must be obtained before any participant is consented. Any amendment to the protocol will require review and approval by the IRB before the changes are implemented to the study. All changes to the consent form(s) will be IRB approved; a determination will be made regarding whether a new consent needs to be obtained from participants who provided consent, using a previously approved consent form.

DO NOT COPY

INVESTIGATOR'S SIGNATURE

The signature below constitutes the approval of this protocol and provides the necessary assurances that this study will be conducted according to all stipulations of the protocol, including all statements regarding confidentiality, and according to local legal and regulatory requirements and applicable US federal regulations and ICH guidelines, as described in the *Statement of Compliance* above.

Principal Investigator or Clinical Site Investigator:

Signed: [redacted]__________________ Date: ______________________

Name: Deborah Jones
Title: Professor, Associate Chair

Investigator Contact Information

Affiliation: The University of North Carolina at Chapel Hill

Address: 267 Davie Hall, Campus Box 3270, Chapel Hill, NC 27599

Telephone: 919-843-2351

Email: djjones@email.unc.edu

DO NOT COPY

1 PROTOCOL SUMMARY

1.1 SYNOPSIS

Title: A Cluster-Randomized Control Trial of a Workplace Resilience Intervention for Child Care Providers' Mental Health & Well-Being

Grant Number: R01AT012620-01A1

Study Description: The goal of this 15-month cluster clinical trial is to compare a resilience and stress management program to a physical activity program for early childhood education (ECE) staff on change in well-being. It will also look to see if changes in well-being continue over time. The study sample will include 80 child care centers. Including 80 ECE center directors and approximately 640 ECE center staff. There will be 3 months of active program participation and a 12 month maintenance period.

Objectives*: The primary objective will test the efficacy of a resilience and stress management program compared to a physical activity program on change in resilience resources and assets (baseline to 3 months). A secondary objective will be to examine change in resilience over a maintenance period (3 months to 9 and 15 months). An additional secondary objective will be to examine change in organizational support and resources, absenteeism, and staff turnover between programs in the two groups.

Endpoints*: There will be 4 time points for this study: Baseline (0 months), 3 months, 9 months, and 15 months.

Study Population: Childcare center staff (N=640) and directors (N=80)

Phase* or Stage: N/A Behavioral Intervention

Description of Sites/Facilities Enrolling Participants: Enrollment of child care centers and individual participants will be coordinated from one university site (University of North Carolina at Chapel Hill). Participants will be clustered within Child care centers.

Description of Study Intervention/Experimental Manipulation: All tasks and activities will be remotely delivered, and guidance will be provided by the behavioral health counselor. Participants will complete their assigned program over four modules spanning 3 months (3 weeks per module).

- For the Physical Activity Arm participants will be encouraged to do moderate to vigorous activities like moderate walking, resistance band training, and group exercise classes with the goal of meeting the current physical activity recommendations for adults.
- For the Resilience Arm participants will be encouraged to take part in techniques including mindfulness, gratitude building, and mediation.

Activities during the active 3-month intervention for both groups will include:

- A 1-hour online orientation with the behavioral health counselor
- Four self-paced modules delivered online, consisting of weekly video lessons (~1 hour each week) and resources about either physical health or resilience training/stress management
- A 1-hour online seminar at the end of each module
- 3-5 text message reminders each week to encourage and support
- All participants will receive a journal. Those in the Resilience intervention will also receive a book.

DO NOT COPY

- Directors of each center will have a 1-hour coaching call every 3 weeks with the counselor for support and preparation.

After the active 3-month intervention, participants will enter a 12-month maintenance period to assess long-term program effectiveness. Participants will continue to have access to program materials and resources, but webinars, coaching calls, and text messages will cease. Participants will be able to contact the counselor if they have questions about the materials.

**Study Duration*:**	42 months from enrollment of first participant to final follow-up measurement
**Participant Duration:**	15-18 months (from screening to final maintenance follow-up)

DO NOT COPY

1.2 SCHEMA

Figure 01. Flow of Study Activities

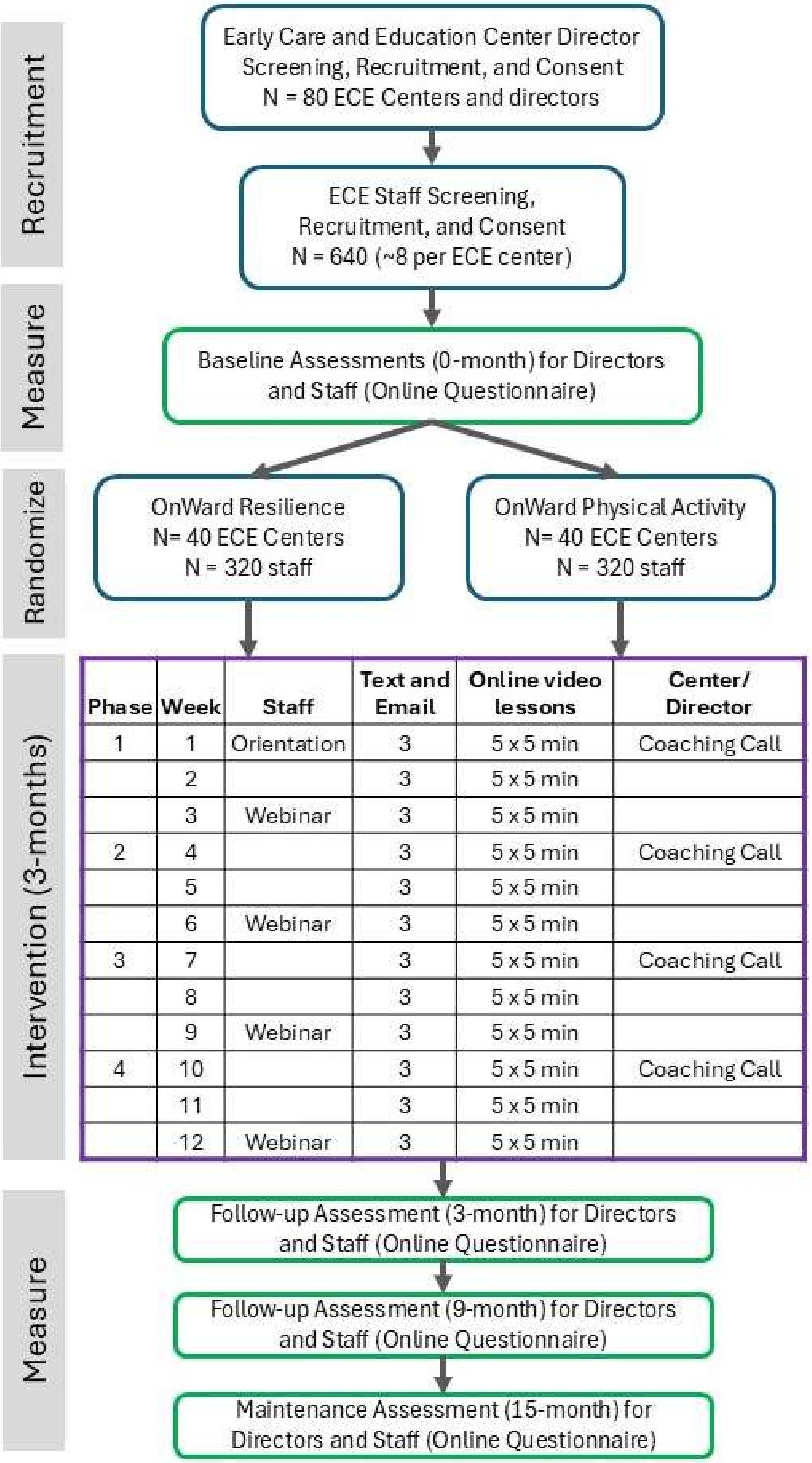

Phase	Week	Staff	Text and Email	Online video lessons	Center/ Director
1	1	Orientation	3	5 x 5 min	Coaching Call
	2		3	5 x 5 min	
	3	Webinar	3	5 x 5 min	
2	4		3	5 x 5 min	Coaching Call
	5		3	5 x 5 min	
	6	Webinar	3	5 x 5 min	
3	7		3	5 x 5 min	Coaching Call
	8		3	5 x 5 min	
	9	Webinar	3	5 x 5 min	
4	10		3	5 x 5 min	Coaching Call
	11		3	5 x 5 min	
	12	Webinar	3	5 x 5 min	

1.3 SCHEDULE OF ACTIVITIES

	Screening	BL Data Collection	Intervention (3mos)	3-month follow-up	9-month follow-up	15-month follow-up
Director: Welcome e-mail	X					
Director: Phone call and Screening	X					
Director: Consent and Online Survey		X				
Director: Follow-up about staff	X	X				
Staff: Online Screener	X					
Staff: Follow-up contact	X					
Staff: Consent and Online Survey		X				
Randomization			X			
Intervention						
Orientation			X			
Director: 4 Coaching Calls			X			
Staff: 3 Webinars			X			
Weekly Texts			X			
Adverse Event Reporting			X			
Follow-up and Maintenance						
Director: Follow-up contact				X	X	X
Director: Online Survey				X	X	X
Staff: Follow-up contact				X	X	X
Staff: Online Survey				X	X	X
Randomly Selected: Process Interview				X		

Note: Survey includes Demographics, Connor-Davison Resilience Scale, General Health Questionnaire, Perceived Stress scale, Social Determinants of Health, Work Well BQ, Work Limitations Questionnaire, Absenteeism, Readiness; Follow-up: Process questions
Note: All contact is remote/online

DO NOT COPY

2 INTRODUCTION

2.1 STUDY RATIONALE

Work-related stressors take a heavy toll on individuals' health and well-being. Which has been made more evident by the increased awareness of essential workers during the pandemic and the still lingering impact on workplace settings.

Resilience programs have arisen as a promising workplace strategy to improve mental health and well-being for those at greatest risk; however, emerging programs are limited by time- and resource-intensive in-person strategies limiting scalability and practicality for the most marginalized of the workforce. Additionally, the literature on resilience programs are largely from preliminary studies that lack a priori sample size calculations, diversity of participants, and long-term maintenance following the intervention. In addition, many trials recruit inadequate control groups, which limits translation.

Consistent with the aim of PAR-24-086 to test a fully remotely delivered clinical trial with no in-person contact, this team has developed and translated the Stress Management and Resilience Training (SMART) program for web-based delivery. Our pilot work demonstrates feasibility, usability, satisfaction, and initial efficacy of the brief self-paced web-based learning sessions (1 hour per week) with only 5-10 minutes of daily practice suggesting a fully powered study is timely and warranted. An adequately powered, cluster randomized controlled trial to test the efficacy of the web-based SMART program compared to a matched attention control in a high-need, diverse, and under-resourced and under-studied segment of essential workers – early childhood education (ECE) workers - will be conducted.

ECE Centers (80 ECE centers consisting of 640 ECE workers) will be randomly assigned to either a Resilience (SMART) or a Physical Activity matched attention control condition with the primary outcome of mean change in resilience assets and resources (Connor-Davison Resilience Scale). Measures will be collected at four timepoints: baseline (0 months), post-intervention (3 months), and long-term maintenance (9 and 15 months). Secondary outcomes will include changes in overall mental health, negative and positive mental health indicators, social support, and organizational assets and resources. Additionally, we will explore potential moderators' (e.g., sociodemographic) influence on treatment effects. The RE-AIM Framework will be used to determine reach and representativeness, and potential for organizational level adoption, implementation, and maintenance of the two programs. This study fills key research gaps of previous resilience work in an underserved population in critical need of mental health and well-being resources with implications for the feasibility and impact of remote programming in other marginalized segments of the workforce.

2.2 BACKGROUND

Work-related stressors knowingly impact individuals' mental health and well-being. Resilience training has been shown to improve our ability to maintain or regain good mental health and well-being despite exposure to stressors or adversity,[1] and thus has received increased attention as a workplace strategy to support employee wellness.[2,3] Although the need and potential benefits of worksite mental health support is evident, there is a shortage of scientifically rigorous, adequately powered, randomized controlled trials (RCT) that assess efficacy of workplace resilience programs. The few RCTs to date fail to include long-term maintenance outcomes or diverse samples and still rely on resource intensive in-person treatment telling us little about their sustainability and generalizability.[1-7] There is a critical need for

DO NOT COPY

rigorous, long-term RCTs to evaluate the efficacy and impact of feasible, fully remote workplace resilience programs to expand their reach.

This study aims to optimize individuals' mental health and well-being and address notable gaps in the resilience intervention literature. SMART, Stress Management And Resilience Training, is a web-based program delivered in four phases over 3-months through three self-paced modules (~1 hour each), a webinar (1-hour), and ongoing support (text/email) during each phase. Our pilot work demonstrates feasibility and usability of the SMART program with essential workers (completion rates >80%) with improvements in resilience assets and resources (Connor-Davison Resilience Scale; CD-RISC) of 5-10% suggesting that a fully powered RCT is critical to confirm and extend these novel findings with populations who may benefit the most, such as childcare workers.

Our preliminary data highlight an opportunity to leverage ECE workers' needs and interest in mental health programs to address limitations in the state of the resilience literature and meaningfully advance both science and practice via fully remote delivery. For example, our prior work with ECE workers reveals that they are diverse (51% minority, 53% enrolled in public assistance programs, 51.6% non-college),[8] low income ($11.65 per hour),[9] with high risk of depression,[10] and little access to wellness programs. Moreover, the recent ECE worker focus groups found that the majority (~70%) would be interested in SMART. Focus on the childcare setting shifts the prevailing research paradigm of resilience training toward a more diverse population who may maximally benefit with implications for other marginalized essential workers as well.

Leveraging the novel collaborative expertise of our team in childcare-based health promotion, e/mHealth delivery, workplace health promotion, resilience interventions, and engaging individuals with low-income, we will evaluate the efficacy of a fully remotely delivered, childcare-based resilience intervention to improve workers' mental health and well-being compared to a physical activity attention control condition. Using a two-arm, cluster RCT, childcare centers (n=80) and workers (n=640) will be randomized to either 1) Resilience (SMART) or 2) a physical activity matched attention control. The 3-month active web-based intervention (1 hour/week, 5-6 minutes daily practice) will be followed by a 12-month, no-contact maintenance phase. Assessments will occur at 0-, 3-, 9-, and 15-months. The primary outcome is change in resilience (CD-RISC) at 3-months.

2.3 RISK/BENEFIT ASSESSMENT

2.3.1 KNOWN POTENTIAL RISKS

There are low, but non-zero, potential psychological (emotional distress) risks; participants may feel distress while completing surveys, or as a result of the increased introspection during the resilience intervention. There are possible social (loss of reputation or standing in community) risks; participants may feel coerced to participation by their employer and possible disagreements related to the increase in discussion about workplace wellness. Finally, there is the possibility of physical (discomfort, injury) risks by increasing physical activity. These risks are unlikely, and we have addressed these risks in section 2.3.3.

2.3.2 KNOWN POTENTIAL BENEFITS

Society will benefit from the proposed study as we identify, develop, and test effective strategies for low-wage workers – a group that tends to be affected by health disparities. Finding effective, low-cost

DO NOT COPY

worksite-based programs that encourage the improvement of the well-being of workers with low wages can be an important strategy for addressing the existing health disparities and may improve the environment for the children enrolled in childcare centers.

ECE workers may benefit from participation in the proposed study in several ways. First, for ECE workers in either intervention program, these efforts have the potential to improve their well-being. For centers, these efforts could also reduce turnover and absenteeism (critical to business productivity) and improve the quality of care they can provide to children. We expect that this information will help workers become more informed decision makers about their health. Additionally, participants in the Physical Activity arm will learn about the benefits of physical activity and may increase their strength.

2.3.3 ASSESSMENT OF POTENTIAL RISKS AND BENEFITS

There are potential psychological (emotional distress) risks. It is possible that childcare workers may experience distress when completing surveys about their occupation-related and daily life stressors. To minimize these risks, we have identified well-established and widely used tools that have been designed to be minimally intrusive. Further, participants always have the right to refuse to complete any assessments. These rights are clearly described in the consent form. While unlikely, the resilience program does promote increased awareness of thoughts, emotions, or personal experiences, which may lead to moments of discomfort or self-reflection. If participants report experiencing any distress or emotional discomfort during the study, resources will be provided to assist as needed. To minimize these risks, we are using an established program with a focus on general personal resilience, not mental health diagnosis. The program does have a behavior health coach, but is not intended as individual counseling for personal struggles or mental health issues. In the highly unlikely event that a staff member feels a participant is considering self-harm the following resources will be provided.

- *We are sorry to hear that it has been a difficult time for you recently. If you are currently having thoughts of harming yourself, please reach out for help. Here are some options:*
    - *National Suicide Prevention Hotline:*
        - *Call 988 or visit https://988lifeline.org and use the "click to chat" feature to be connected to someone immediately*
    - *Crisis Text Line: Text "HOME" to 741741*
    - *BlackLine: 1-800-604-5841*
    - *Trans Lifeline: 1-877-565-8860*
    - *The Trevor Project: 1-866-488-7386*
    - *Contact your primary care or mental health provider*
    - *Consider going to your local emergency department*
- **NOTE: Many of these resources could utilize restrictive interventions such as active rescues (wellness or welfare checks) involving law enforcement or emergency services. You can ask if this is a possibility at any point in your conversation if this is a concern for you.*

Additionally, the research team will check in with each participant regularly via text to monitor any possible adverse events experienced as a result of the study. If participants share that they have experienced any adverse events, they will receive a link to a Qualtrics survey to collect more details, and a member of the research team will reach out to share external resources and support as needed. Participation in the study is voluntary, and participants may withdraw at any time without consequence.

DO NOT COPY

There are potential social (loss of reputation or standing in community) risks. One potential risk is that childcare staff may feel coerced into participating in this research study by their director. To minimize risks for staff, we will obtain assurances from directors, owners (if applicable), and assistant directors (if applicable) that a staff member's decision to participate in any aspect of the study or discontinue at any time will not affect their job standing, or performance ratings. In addition, it is possible (though unlikely) that the proposed intervention may have negative effects on social relationships among workers in the participating childcare centers. We expect that the intervention will lead to an increase in discussions in the childcare centers about health in general and about mental health and well-being. During our 10 years of working in worksites, we have never witnessed these discussions turning into disagreements, debates, or even arguments between workers, but we recognize that it is possible. Such negative interactions could then impact the social relationships between workers in these childcare centers. However, we believe it is unlikely that the proposed topics will spark debates or arguments serious enough to have lasting negative effects on existing social relationships. Moreover, messages will be framed using a strengths-based approach letting workers know that there is something they can do to improve their mental health and well-being. This will likely generate positive discussions. It is our experience that these types of discussions tend to bring people together in a positive (vs. negative) way.

There are potential physical risks (discomfort, injury). Increasing one's physical activity, particularly for individuals who are inactive, is infrequently accompanied by mild discomfort and soreness of muscles for a short period. Additionally, rare mild or moderate injury is possible as the result of participation in physical activity. In the study information and consent we recommended that participants consult their primary care physician before making major changes to their physical activity habits.

DO NOT COPY

3 OBJECTIVES AND ENDPOINTS

OBJECTIVES	ENDPOINTS	JUSTIFICATION FOR ENDPOINTS
Primary		
Change in Resilience from Baseline to 3 months	Resilience of ECE staff will be assessed using the Connor-Davidson Resilience Scale (CD-RISC). ECE staff will fill it out through an online survey. The CD-RISC is a 25-item scale. Scores range from 0-100 with higher scores reflecting greater resilience.	The CD-RISC is the most commonly used assessment of resilience in research.[1] Data are used to calculate an overall resilience score. The overall score has demonstrated internal consistency (Cronbach's alpha = (0.89), been shown to be negatively correlated with stress (r=-076, $p<0.001$),[11] and sensitive to change in several of our team's previous pilot studies.[12,13,14]
Secondary		
Change in Resilience from 3 months to 9 months	Resilience of ECE staff will be assessed using the Connor-Davidson Resilience Scale (CD-RISC). ECE staff will fill out through an online survey. The CD-RISC is a 25-item scale. Scores range from 0-100 with higher scores reflecting greater resilience.	The CD-RISC is the most commonly used assessment of resilience in research.[1] Data are used to calculate an overall resilience score. The overall score has demonstrated internal consistency (Cronbach's alpha = (0.89), been shown to be negatively correlated with stress (r=-076, $p<0.001$),[11] and sensitive to change in several of our team's previous pilot studies.[12,13,14]
Change in Resilience from 9 to 15 months	Resilience of ECE staff will be assessed using the Connor-Davidson Resilience Scale (CD-RISC). ECE staff will fill out through an online survey. The CD-RISC is a 25-item scale. Scores range from 0-100 with higher scores reflecting greater resilience.	The CD-RISC is the most commonly used assessment of resilience in research.[1] Data are used to calculate an overall resilience score. The overall score has demonstrated internal consistency (Cronbach's alpha = (0.89), been shown to be negatively correlated with stress (r=-076, $p<0.001$),[11] and sensitive to change in several of our team's previous pilot studies.[12,13,14]
Change in Well-Being from Baseline to 3 months	Global assessment of well-being will be measured through the General Health Questionnaire. This is a 30-item survey captures a broad spectrum of psychological distress encompassing emotional, cognitive, and social aspects of mental health. Scores range from 0-90, with higher scores indicating greater psychological distress.	The Global Health Questionnaire was recommended in a review of resilience interventions for its ability to capture a global assessment of mental health, including anxiety, difficulty coping, depression, feelings of incompetence, and social dysfunction. Cronbach's alpha= 0.92

DO NOT COPY

OBJECTIVES	ENDPOINTS	JUSTIFICATION FOR ENDPOINTS
Change in Organizational Support and Resources from Baseline to 3 months	Organizational support and resources will be assessed using the NIOSH worker well-being questionnaire (WellBQ). Specifically, eight Likert-type items from the supportive work culture and health culture at work sections of the survey will be averaged to create this outcome. Items are rated on 1 to 4 scale from strongly disagree to strongly agree, with the final outcome being an average score ranging from 1.00 to 4.00.	The NIOSH WellBQ is a well researched and strongly developed assessment of worker well-being across multiple domains.
Absenteeism from Baseline to 3 months	Staff absenteeism will be assessed by asking staff how often they "missed a full day of work" and "missed 1/2 day of work" in the past 4 weeks. Directors will also be asked "On average, how many days per month do staff miss a full day of work when scheduled to be at your center?". Items will be combined to estimate "Days per month staff missed work" at a center.	Common questions used to assess missed work and impact on organizational function.
Staff Turnover rate from Baseline to 3 months	Staff turnover will be assessed by asking directors: "How many total staff work at center?" "How many staff have left, quit, or been fired in the past 3 months?", and "how many of those staff have you replaced in the past three months or are currently trying to replace?". Questions will be asked at baseline and 3 month follow-up. Turnover rate for a center will be expressed as: ((# staff left job-(#staff not need replacing)/(# total staff)) * 100 = % turnover.	A study specific survey will measure 4 types of turnover: voluntary (by choice), involuntary (reasons beyond their control), external (leaves employer), and internal (changes job with employer). Turnover rate will be calculated using data provided by the employer. The precise rate (i.e., turnover events/workers employed in a fiscal year) will be calculated.
Tertiary/Exploratory		
Readiness for change	*Readiness to perform or implement 20 intervention related changes will be assess. Items are score on a 7-point Likert-type scale.*	Theoretically to more “ready” or willing a person is to change, or add, health-related behaviors to more change they will experience through behavioral health intervention.

DO NOT COPY

4 STUDY DESIGN

4.1 OVERALL DESIGN

This study will use a cluster-randomized control trial to evaluate change in resilience assets and resources for early childhood education (ECE) center staff. To evaluate the impact, measurements will be collected at four time points, baseline, post intervention (3 months), and maintenance (9 months and 15 months). All data collection will be completed remotely, using self-report surveys and phone/audio conferencing interviews. ECE centers will be randomized to receive either the resilience intervention or a physical activity (attention matched control) intervention.

Randomization.
Centers will serve as the unit of randomization. Soon after baseline testing, each center will be randomized to either the Resilience program or the Physical Activity program. Randomization tables with a permuted block approach (block sizes of 2-4) will be used to ensure equal distribution between arms throughout the randomization period. Intervention assignment will only be known to the project manager and the behavioral health counselor delivering the intervention. Investigators and data collectors will be kept blinded.

Intervention
All tasks and activities will be remotely delivered, and guidance will be provided by the behavioral health counselor. Participants will complete their assigned program over four modules spanning 3 months (3 weeks per module). For the Physical Activity program participants will be encouraged to participate in activities that meet the current physical activity recommendations for adults. These would include moderate to vigorous activities like moderate walking, resistance band training, and group exercise classes. For the Resilience program participants will be encouraged to take part in techniques including mindfulness, gratitude building, and mediation.
Activities will include:

- A 1-hour online orientation with the behavioral health counselor
- Four self-paced modules delivered online, consisting of weekly video lessons (~1 hour each week) and resources about either physical health or resilience training/stress management
- A 1-hour online seminar at the end of each module
- 3-5 text message reminders each week to encourage and support
- All participants will receive a journal to reflect in. Those in the Resilience intervention will also receive a book.
- Directors only: a 1-hour coaching call every 3 weeks with the behavioral health counselor for support and preparation prior to the start of each module

Follow-up Data Collection:
Once the program is completed data will be collected at 3 time points post-intervention: after 3 months, after 9 months, and after 15 months. Similar to initial data collection, participants will complete an online survey to measure resilience, mental health, and process related questions about their experience with the assigned program. Participants will be randomly selected to be interviewed over the phone for 30-45 minutes about their experience with their assigned program at 3 months.

Maintenance Period:

DO NOT COPY

After the active 3-month intervention, participants will enter a 12-month maintenance period to assess long-term program effectiveness. Participants will continue to have access to their assigned program materials and resources, but the webinars and coaching calls will cease. Participants will still be able to contact the behavioral health counselor if they have questions about the materials provided.

4.2 SCIENTIFIC RATIONALE FOR STUDY DESIGN

Recently, there has been a call for resilience researchers to move beyond single-arm and wait-list control study designs to those that are randomized, controlled, and include matched attention comparison groups.[1,7] Although there is no current consensus on what type of attention control is best for resilience research, the use of physical health promotion interventions (e.g., physical activity, nutrition) have been shown to be feasible and acceptable. Therefore, we have chosen our teams' physical activity promotion program used in previous[17-23] and ongoing (R01 DK128174) childcare-based worksite wellness trials. Offering an alternative health promotion program will be perceived as a positive alternative to a waitlist for workers randomized to the control group. Additionally, this affords an opportunity to assess the efficacy of the Resilience program compared to a program that has the potential to be a comparable, or alternative, therapy for stress and anxiety reduction for ECE workers. In the attention control group, participants will be provided a low-cost activity tracker (e.g., Amazfit Band). Similar to the Resilience program components will be delivered in four phases over 3-months with implementation supported by trained research staff. Timeline and logistical components will match the Resilience program.

4.3 JUSTIFICATION FOR INTERVENTION

The intervention delivery mode to test a fully remotely delivered clinical trial with no in-person contact is consistent with the aim of PAR-24-086. Members of our team have demonstrated the feasibility and initial efficacy of a web-based delivery approach of SMART in both the school-based setting and nursing workforce. In a recent 12-month pilot trial, 55 (78.6% completion rate) public school teachers and other staff completed a web-based delivered SMART. To follow up on this promising work we assessed interest in the SMART program with ECE workers and assessed resilience and related constructs. We conducted focus groups with ECE directors (n=17) and staff (n=15). Workers clearly recognized personal stress as a pervasive problem. Workers also indicated that the time requirement to participate and lack of access to mental health programs as major concerns. There was great interest in workplace stress reduction programs, with a majority (~70%) indicating an interest in the SMART program. These data highlight an opportunity to leverage childcare workers' needs and interests in the SMART program to improve mental health and well-being and the importance of options that decrease time requirements of such programs. Based on this information the SMART program was tailored to ECE staff and intervention period is set for 3 months and includes an orientation, use of texting and a website for lesson and resource delivery, 3 webinars, and 4 coaching calls.

4.4 END-OF-STUDY DEFINITION

A participant is considered to have completed the study if he or she has completed the online baseline assessment and the 3-month, 9-month, and 15-month follow-up online assessments. The end of the study is defined as completion of the 15-month follow-up assessment shown in the Schedule of Activities (SoA), **Section 1.3**.

DO NOT COPY

5 STUDY POPULATION

The 640 estimated ECE workers will be nested within the 80 participating ECE centers.

5.1 INCLUSION CRITERIA

In order to be eligible to participate in this study, an ECE center must meet all of the following criteria:
1. Be a licensed ECE center with no plans to close in the next 2 years
2. Have been in operation for at least 1 year

In order to be eligible to participate in this study, an individual must meet all of the following criteria:
1. Be employed at a participating ECE center
2. Be at least 18 years old
3. Be able to read and speak English
4. Have access to an online resource (e.g., smartphone, tablet, computer)
5. Be willing to receive text messages

5.2 EXCLUSION CRITERIA

No Exclusion Criteria

5.3 LIFESTYLE CONSIDERATIONS

N/A

5.4 SCREEN FAILURES

Participants are screened ahead of consent so screen failures should not occur.

5.5 STRATEGIES FOR RECRUITMENT AND RETENTION

Recruitment
Per our sample size/power, 640 childcare workers will be required to ensure adequate statistical power for assessment of our primary aim, resilience resources and assets as assessed by the Connor-Davidson Resilience Scale. These workers will be nested within 80 childcare centers. Recruitment efforts will first identify potential childcare centers using the North Carolina Division of Child Development and Early Education website, a publicly accessible database of all licensed childcare centers in North Carolina. Leveraging our existing relationships with state-wide agencies, we will engage community partners in North Carolina (e.g., Smart Start programs, Partnership for Children, Go NAPSACC technical assistants in relevant counties), to provide a soft introduction to the research study that endorses participation (e.g., email announcements, flyer, regular communication channels). These local introductions encourage participation because centers are learning about the research study from someone they know and trust. The research team then follows up with direct contacts using multiple communication channels (i.e., email invitation and phone calls) to recruit centers, directors, and staff. Contacts always start with the center/director to confirm interest and eligibility. The director will be emailed a link to a consent form and survey once they agree to participate.

DO NOT COPY

Once a director is on board, the research team works with the director to engage and recruit center staff (e.g., email/post a flyer, talk virtually at a staff meeting). Center staff will fill out an eligibility screener and be given multiple opportunities to learn more about the project before agreeing to participate. Eligible centers must have at least 8 childcare workers employed; however, no upper limit will be imposed on the number of workers that can take part in order to accommodate the needs of larger centers.

In North Carolina, there are 4,456 licensed childcare centers. We conservatively estimate that at least half of our potential pool will meet eligibility criteria. Thus, we are confident we can enroll 80 (3.6%) of these centers to meet our recruitment goal. We anticipate recruiting 5 centers per month on average, completing all 80 centers in about 2 years. The population of childcare workers is highly diverse (99% women, 51% minority, 53% enrolled in public assistance programs, 51.6% non-college) and generally low income. Our study design shifts the prevailing research paradigm of resilience intervention toward a more diverse population which may maximally benefit from availability and implementation of resilience principles and training.

Incentives

Participants will receive incentives for completing different parts of the study. Centers (through the director) will receive $100 in gift cards for participating at baseline and again at 3 months for a total of $200 in gift cards. They will receive these incentives once all staff have filled out their online survey for the relevant timepoint. Participating center staff will receive $50 in gift cards for data collection at baseline, 3 months, 9 months, and 15 months for a total of $200 over 4 time points. They will receive these incentives as they fill out their relevant timepoint survey. Once randomized, centers will receive a welcome box. The Resilience welcome box will include items to welcome and help support center staff: A SMART book, a journal, a stress ball, an eye mask, and general instructions. Centers randomized to the Physical Activity arm will receive a welcome box including: an activity device (e.g., Amazefit), a journal, water bottle, a stretchy band, and general instructions. Participants in both arms will have the opportunity to receive up to 8 contact hours during the 3 months active intervention. These are earned through watching/completing the videos and lessons related to the module and completing an online survey where they answer questions about the relevant module and reflect on that module. A certificate or each module will be emailed once all items are confirmed completed. A random sample of participants will be chosen to participate in a structured interview at 3 months. Each participant will receive $25 in gift cards for their time once they complete the interview.

We do not anticipate any potential for coercion given that the level of incentives as planned are not excessive. The maximum individual incentive at any point in time is expected to be $100. As small to medium size businesses, the maximum center incentive of $100 is not viewed as a large or coercive amount of money. While contact hours are required for some ECE workers, there are many ways to obtain these hours.

DO NOT COPY

6 STUDY INTERVENTION(S) OR EXPERIMENTAL MANIPULATION(S)

6.1 STUDY INTERVENTION(S) OR EXPERIMENTAL MANIPULATION(S) ADMINISTRATION

6.1.1 STUDY INTERVENTION OR EXPERIMENTAL MANIPULATION DESCRIPTION

Centers randomized to **Resilience** will receive the Stress Management And Resilience Training program. Participating staff will be led through the program by a behavioral health counselor for 3 months. This counselor will orient participants to the Resilience program and the 4 modules. The counselor will also provide 4 coaching calls with directors (at the start of each module). Additionally, the counselor will provide a webinar every 3 weeks (at the end of each module) to all participants. Participants will have access to video lessons and resources. They will receive weekly reminders and encouragement through text messages. The Resilience program focuses on drawing on resilience assets and resources, both internal and external (mechanism). Participants will have access to materials (e.g., lessons, videos, webinars, coaching calls) supporting the adoption of strategies for improving well-being. Behavior change strategies used are meant to increase intervention adherence and improve well-being and resilience.
Centers randomized to the control arm will receive a **Physical Activity Program**. Participating staff will be led through the program by a behavioral health counselor for 3 months. This counselor will orient participants to the physical activity program and the 4 modules. The counselor will also provide 4 coaching calls with directors (at the start of each module). Additionally, the counselor will provide a webinar every 3 weeks (at the end of each module) to all participants. Participants will have access to video lessons and resources. They will receive weekly reminders and encouragement through text messages. The Physical Activity program focuses on supporting physical activity health habits (mechanism). Participants will have access to materials (e.g., lessons, videos, webinars, coaching calls) supporting the adoption of strategies of evidence-based strategies for physical activity goals. Behavior change strategies used are meant to help establish, improve or maintain healthy physical activity habits.

6.1.2 ADMINISTRATION AND/OR DOSING

All tasks and activities will be delivered through virtually meeting, phone calls, text messages, website lessons, email, and guidance will be provided by the behavioral health counselor. As required by the grant mechanism, not in-person intervention delivery or participant contact is allowed.

Participants will complete their assigned program over four modules spanning 3 months (3 weeks per module). For the **Resilience** program participants will be encouraged to take part in techniques including mindfulness, gratitude building, and meditation. For the **Physical Activity** program participants will be encouraged to participate in activities that meet the current physical activity recommendations for adults. These would include moderate to vigorous activities like moderate walking, resistance band training, and group exercise classes (intensity).

Activities will include: A 1-hour online orientation with other participants and the behavioral health counselor; four self-paced modules delivered online, consisting of weekly video lessons (~1 hour each week) and resources about either physical health or resilience training/stress management; A 1-hour online seminar with other participants and the behavioral health counselor at the end of each module; 3-5 text message reminders each week to encourage and support. All participants will receive a journal to reflect in daily. Those in the Resilience intervention will also receive a book containing information mirroring the video lessons.

DO NOT COPY

Directors only: a 1-hour one-on-one coaching call every 3 weeks with the behavioral health counselor for support and preparation prior to the start of each module

6.2 FIDELITY

6.2.1 INTERVENTIONIST TRAINING AND TRACKING

Resilience. The Behavioral Health Counselor will complete the Trainer Skills Intensive program (~6 months), conducted Dr. Sood (collaborator), to become a certified resilience trainer using a modified SMART program to participants. This standardized program provides the necessary knowledge and skills of the science and art of resilience.

Physical Activity. The Behavioral Health Counselor will receive standard training and certification which includes a mix of written materials, didactic sessions with the investigative team, and hands-on/practice-based exercises (~6 months) to be prepared to deliver this program to participants. Dr. Hales has extensive experience with physical activity interventions and will lead this training.

All orientations, coaching calls, and webinars will be recorded; 10% will be rated by 2 independent raters using a fidelity checklist. These data will be used to assess competence and adherence to content standards. The automated nature of the lesson materials and feedback messages in this study ensure fidelity of core intervention content following orientation but will be routinely monitored by the research team to ensure protocol compliance. Although all research staff-led communication will end following week 12, participants will still have access to past behavioral lessons for one year. Use of lessons will continue to be tracked during this no contact follow-up period.

6.3 MEASURES TO MINIMIZE BIAS: RANDOMIZATION AND BLINDING

Although it is not feasible to blind participants to group assignment in health behavior interventions, both groups in this study will be blinded to the primary research questions and hypotheses to decrease the likelihood that the design will affect validity of results. All investigators and follow-up data collectors will remain blinded. Only the project manager and the health behavior counselor will be unblinded.

6.4 STUDY INTERVENTION/EXPERIMENTAL MANIPULATION ADHERENCE

Attendance will be taken by the behavioral health counselor for the virtual coaching calls with center directors and webinars with child care staff. The behavioral health counselor will also use Zoom meeting reports to track attendance and the duration of time each participant spent in webinars and coaching calls.

Exposure to other intervention materials will also be tracked, including web-trainings (i.e., user engagement data), texts (i.e., delivery and response reports from the messaging platform), and books and journals (i.e., participant self-report and reflection of journal prompts in webinars).

In addition to website user reports, Zoom meeting reports, text delivery and response reports, and self-reporting from participants, the behavioral health counselor will maintain a tracking database with notes and completion logs for all participants.

No aspects of the intervention are mandatory to remain an active participant and there are no instances where we would withdraw an individual subject from the study.

DRAFT COPY

During biweekly meetings, the project manager and data manager will also report on study protocols and any adherence questions or issues. The content of these reports will vary depending on the current study activities. During implementation, the project manager will create reports summarizing the process data collected and completion of core components of each study group.

6.5 CONCOMITANT THERAPY

For this protocol, participants may be enrolled in ongoing exercise classes, fitness programs, or therapy (group or individual). Current use of health or wellness programs/therapies will be assessed in questionnaires at each time measurement time point. If a participant responds yes to participation, they will be asked how long they have been involved in the program.

6.5.1 RESCUE THERAPY

N/A

DO NOT COPY

7 STUDY INTERVENTION/EXPERIMENTAL MANIPULATION DISCONTINUATION AND PARTICIPANT DISCONTINUATION/WITHDRAWAL

7.1 DISCONTINUATION OF STUDY INTERVENTION/EXPERIMENTAL MANIPULATION

There are no instances where the research team would discontinue a study participant from the intervention.

7.2 PARTICIPANT DISCONTINUATION/WITHDRAWAL FROM THE STUDY

There are no instances where the research team would discontinue a study participant from the study. If a study participant withdraws themselves from the study post-randomization, they will not be replaced.

7.3 LOST TO FOLLOW-UP

A participant will be considered lost to follow-up if study team members are unable to reach the participant after at least 5 attempts, including email, text, and direct phone call, during all follow-up timepoints (i.e., 3 months, 9 months, 15 months). Maintaining study contacts during the 12-month maintenance period will be important for data collection. Each month participants will be sent an email or text thanking them for participating and asking if we should update any of their contact information.

DO NOT COPY

8 STUDY ASSESSMENTS AND PROCEDURES

8.1 ENDPOINT AND OTHER NON-SAFETY ASSESSMENTS

As required by the grant mechanism all recruitment, screening, and measurement procedures must take place virtually or over the phone. No in-person contact with participants is allowed. For screening and recruitment, we will first identify potential childcare centers using the North Carolina Division of Child Development and Early Education website, a publicly accessible database of all licensed childcare centers in North Carolina. Leveraging our existing relationships with state-wide agencies, we will engage community partners (e.g., Smart Start programs, Partnership for Children, Go NAPSACC technical assistants in relevant counties), to provide a soft introduction to the research study that endorses participation (e.g., email announcements, flyer, regular communication channels).

The research team then follows up with direct contacts using multiple communication channels (i.e., email invitation and phone calls) to recruit centers, directors, and staff. Contacts always start with the center director/owner to confirm interest and eligibility. Centers must have at least 8 childcare workers to be eligible. Once a director is screened and agrees to participate, the research team works with the director to engage and recruit center staff (e.g., email/post a flyer, talk virtually at a staff meeting). Center staff will fill out an eligibility screener and be given multiple opportunities to learn more about the project before agreeing to participate. A complete list of inclusion and exclusion criteria can be found in sections 5.1 and 5.2.

After screening and verbal agreement, directors and staff will be sent informed consent and a demographic questionnaire. All measures will be collected through the Qualtrics online survey platform. After consent is complete a link to the full baseline survey will be sent to each participant. If the baseline questionnaire is not completed within 4-days, study staff will attempt to call or text participants to encourage they finish surveys. Staff with incomplete consent, demographics, or primary outcome measures will not be randomized. During each follow-up measurement period (3-, 9-, and 15-months), participants will be sent a link to a survey and reminders for 7 days (1 per day until survey completed). If a survey is not completed within 4-days, study staff will attempt to contact a participant by phone or text.

8.2 SAFETY ASSESSMENTS

At enrollment, we will inform child care centers and their participating providers to contact the project manager if they feel an injury, illness or related event may have occurred to them as a result of their participation in this study. Additionally, as part of both intervention arms, participants will be texted monthly to report on any adverse events that may have occurred. If such an event occurs or is reported, the project manager will collect a detailed description of the event, the adverse outcome, severity of the adverse event, and whether or not participants viewed it as related to the study. This report will then be reviewed by the DSMB and Adverse event reports will be submitted to the IRB after completion by the DSM officer if related to participation in the study.

As required by IRB the study will also include common statements related to seeking medical care or prescreening when starting, or during, a wellness program.

DO NOT COPY

For the RESILIENCE arm the following will be included in provided materials…

> *The information in this program is not a substitute for medical advice. This resilience program does promote increased awareness of thoughts, emotions, and personal experiences, which may lead to moments of discomfort or self-reflection. If you experience any distress or emotional discomfort during the study, please seek the advice and counseling of a trained mental health professional. If needed, resource contacts can be provided by study staff.*

While the RESILIENCE program does have a behavior health coach, it is not intended as individual counseling for personal struggles or mental health issues. In the highly unlikely event that a staff member feels a participant is considering self-harm IRB also requires the following message be sent through email or text:

> *We are sorry to hear that it has been a difficult time for you recently. If you are currently having thoughts of harming yourself, please reach out for help. Here are some options:*
>
> - *National Suicide Prevention Hotline: Call 988 or visit https://988lifeline.org and use the "click to chat" feature to be connected to someone immediately*
> - *Crisis Text Line: Text "HOME" to 741741*
> - *BlackLine: 1-800-604-5841*
> - *Trans Lifeline: 1-877-565-8860*
> - *The Trevor Project: 1-866-488-7386*
> - *Contact your primary care or mental health provider*
> - *Consider going to your local emergency department*
>
> **NOTE: Many of these resources could utilize restrictive interventions such as active rescues (wellness or welfare checks) involving law enforcement or emergency services. You can ask if this is a possibility at any point in your conversation if this is a concern for you.*

For the PHYSICAL ACTIVITY arm the following statement will be included in the materials provided:

> *The information in this program is not a substitute for medical advice. Before starting any new physical activity routine, especially if you have any health concerns, consult a health care professional to determine the appropriate level of exercise for your individual needs. The CDC recommends that adults engage in at least 150 minutes of moderate-intensity aerobic activity per week, but the appropriate level of activity for you should be determined by your doctor.*

8.3 ADVERSE EVENTS AND SERIOUS ADVERSE EVENTS

8.3.1 DEFINITION OF ADVERSE EVENTS

An adverse event (AE) is any untoward medical occurrence in a subject during participation in the clinical study or with use of the experimental agent being studied. An adverse finding can include a sign, symptom, abnormal assessment (laboratory test value, vital signs, electrocardiogram finding, etc.), or any combination of these regardless of relationship to participation in the study.

8.3.2 DEFINITION OF SERIOUS ADVERSE EVENTS

A serious adverse event (SAE) is one that meets one or more of the following criteria:

DO NOT COPY

- Results in death
- Is life-threatening (places the subject at immediate risk of death from the event as it occurred)
- Results in inpatient hospitalization or prolongation of existing hospitalization
- Results in a persistent or significant disability or incapacity
- Results in a congenital anomaly or birth defect

An important medical event that may not result in death, be life threatening, or require hospitalization may be considered an SAE when, based upon appropriate medical judgment, the event may jeopardize the subject and may require medical or surgical intervention to prevent one of the outcomes listed in this definition.

8.3.3 CLASSIFICATION OF AN ADVERSE EVENT

8.3.3.1 SEVERITY OF EVENT

The following scale will be used to grade adverse events:

1. Mild: no intervention required; no impact on activities of daily living (ADL)
2. Moderate: minimal, local, or non-invasive intervention indicated; moderate impact on ADL
3. Severe: significant symptoms requiring invasive intervention; subject seeks medical attention, needs major assistance with ADL

8.3.3.2 RELATIONSHIP TO STUDY INTERVENTION/EXPERIMENTAL MANIPULATION

To assess relationship of an event to study intervention, the following guidelines will be used:

1. Related (Possible, Probable, Definite)
   a. The event is known to occur with the study intervention.
   b. There is a temporal relationship between the intervention and event onset.
   c. The event abates when the intervention is discontinued.
   d. The event reappears upon a re-challenge with the intervention.
2. Not Related (Unlikely, Not Related)
   a. There is no temporal relationship between the intervention and event onset.
   b. An alternate etiology has been established.

8.3.3.3 EXPECTEDNESS

The Study MPIs and DSMB will be responsible for determining whether an SAE is expected or unexpected. An adverse event will be considered unexpected if the nature, severity, or frequency of the event is not consistent with the risk information previously described for the intervention.

8.3.4 TIME PERIOD AND FREQUENCY FOR EVENT ASSESSMENT AND FOLLOW-UP

Collection of adverse events will use both active and passive assessments methods.

- Active assessment will occur in two ways:
  - During our outcome measurement at 3, 9, and 15 months, will collect data regarding possible adverse events using the Unwanted Events and Adverse Treatment Reaction Checklist.

DO NOT COPY

        - This checklist is recommended by methodical review for resilience interventions to assess unwanted or adverse effects of the training program.
    - Each month during the intervention period, we will use the 3-way messaging capabilities of our text messaging platform, or email (participant preference), to ask about possible adverse events.
        - A message asking "Over the past month. have you experienced a physical injury, distress, anxiety, or illness as a result of your participation in this study? Yes/No" will be sent. Those who do not reply will be sent a second message within 24 hours with the same text. Those that reply yes to either message will be contacted to gather additional details.
- Passive assessment will be ongoing during the intervention period. At enrollment, we will inform all participants to contact the project manager or interventionist if they think an injury or illness they have had during the study period may have occurred as a result of their participation in this study.

In our previous center-based intervention studies, no injuries related to participation have occurred. We therefore anticipate that injury risk is minimal. If such an event occurs, the project manager will collect a detailed description of the event, the adverse outcome, severity of the adverse event, and whether or not participants viewed it as related to the study. Information about the event will be shared with the MPIs and the Independent Monitoring Committee (IMCR). The IMC, as an unbiased party, will be responsible for categorizing it as: definitely unrelated, or unlikely, possibly, probably, or definitely related to study participation. Adverse event reports will be submitted to the IRB after completed by the IMC. A summary of these reports will be submitted to the project officer on an annual basis.

8.3.5 ADVERSE EVENT REPORTING

Summary of reporting procedures:

- Adverse Event Reports
    - If an AE occurs, the project manager will collect a detailed description of the event, the adverse outcome, severity of the adverse event, and whether or not participants viewed it as related to the study. This information will then be reviewed by the IMC, who will determine relationship to the study (definitely unrelated, or unlikely, possibly, probably, or definitely related), expectedness of the AE (expected or unexpected), and severity of the AE (mild, moderate, servere) to study participation.
    - SAE reports will be submitted by the MPIs to the IRB and NCCIH immediately after IMC review. Non-severe AEs will be reported to IRB, as required, and to NCCIH in the annual report. The monthly (MPIs), or quarterly (IMC), reports will include a summary of all AEs for review by MPIs and IMC.

8.3.6 SERIOUS ADVERSE EVENT REPORTING

Given the nature of the study and its relation to our prior work, we believe it is very unlikely that an unanticipated SAE will occur that is possibly related to the study intervention. That said, If such an event occurs, the project manager will collect a detailed description of the event, the adverse outcome, severity, and whether or not participants viewed it as related to the study. Information about the event will be shared with the MPIs and the IMCR. The IMC, as an unbiased party, will be responsible for

DO NOT COPY

categorizing it as: definitely unrelated, or unlikely, possibly, probably, or definitely related to study participation.

- Unexpected fatal or life-threatening SAEs related to the intervention will be reported to NCCIH Program Officer, the IMC, and IRB within 3 days of the PIs becoming aware of the event. Other serious and unexpected AEs related to the intervention will be reported within time frame required by IRB.
- Anticipated or unrelated SAEs will be handled in a less urgent manner but will be reported to the IMC, IRB, and other oversight organizations in accordance with their requirements. and will be reported to NCCIH on an annual basis.
- All other AEs documented during the course of the trial will be reported to NCCIH in the annual report and in the annual AE summary which will be provided to NCCIH and to the IMC, including The Independent Safety Monitor(s) Report which will state that all AEs have been reviewed.

8.3.7 REPORTING EVENTS TO PARTICIPANTS

Unrelated events will not be reported to participants, unless IRB asks for a specific case to be reported. When required by IRB, events considered related to study participation will be reported back to the individual involved. If deemed necessary by IRB, a general report (no identifiable info) of an SAE may be reported to all participants by email.

8.3.8 EVENTS OF SPECIAL INTEREST

N/A

8.3.9 REPORTING OF PREGNANCY

Being, or becoming, pregnant does not preclude childcare staff from study participation. Individuals who report being pregnant during recruitment will be asked to provide medical clearance for participation in physical activity before they are enrolled. During the study, pregnancy status will be monitored by asking participants "if they are currently pregnant" at the 0, 3, 9, and 15 month measurements time points and by asking participants to inform the project manager or interventionist if they become pregnant during the study. Current physical activity recommendations have been found to be safe for those who are pregnant. Specific recommendations and resources regarding physical activity during pregnancy and the post-partum period will be provided for all participants. Anyone reporting pregnancy will be personally informed about those resources after informing the study team about their pregnancy status.

8.4 UNANTICIPATED PROBLEMS

8.4.1 DEFINITION OF UNANTICIPATED PROBLEMS

The Office for Human Research Protections (OHRP) considers unanticipated problems involving risks to subjects or others to include, in general, any incident, experience, or outcome that meets **all** of the following criteria:

- Unexpected in terms of nature, severity, or frequency given
  - (a) the research procedures that are described in the protocol-related documents, such as the IRB-approved research protocol and informed consent document

DO NOT COPY

    - (b) the characteristics of the subject population being studied;
- Related or possibly related to participation in the research ("possibly related" means there is a reasonable possibility that the incident, experience, or outcome may have been caused by the procedures involved in the research); and
- Suggests that the research places subjects or others at a greater risk of harm (including physical, psychological, economic, or social harm) than was previously known or recognized.

8.4.2 UNANTICIPATED PROBLEMS REPORTING

Incidents or events that meet the OHRP criteria for unanticipated problems require the creation and completion of an unanticipated problem report form. OHRP recommends that investigators include the following information when reporting an adverse event, or any other incident, experience, or outcome as an unanticipated problem to the IRB:

- Appropriate identifying information for the research protocol, such as the title, investigator's name, and the IRB project number;
- A detailed description of the adverse event, incident, experience, or outcome;
- An explanation of the basis for determining that the adverse event, incident, experience, or outcome represents an unanticipated problem;
- A description of any changes to the protocol or other corrective actions that have been taken or are proposed in response to the unanticipated problem.

To satisfy the requirement for prompt reporting, unanticipated problems will be reported using the following timeline:

- Unanticipated problems that are serious adverse events will be reported to the IRB, IMC, and NCCIH within 7 days of the investigator becoming aware of the event.
- Any other unanticipated problem will be reported to the IRB, IMC, and NCCIH within 14 days of the investigator becoming aware of the problem.

All unanticipated problems should be reported to appropriate institutional officials (as required by an institution's written reporting procedures), the supporting agency head (or designee), and OHRP within one month of the IRB's receipt of the report of the problem from the investigator.

8.4.3 REPORTING UNANTICIPATED PROBLEMS TO PARTICIPANTS

N/A

DO NOT COPY

9 STATISTICAL CONSIDERATIONS

9.1 STATISTICAL HYPOTHESES

We hypothesize that:

- Primary Endpoint(s):
  - Participants in the RESILIENCE program arm will demonstrate greater improvements in resilience assets and resources as measured by CD-RISC scores at 3-months (post-treatment) relative to those in the attention control arm (physical activity program).

- Secondary Endpoint(s):
  - Participants in the RESILIENCE arm will demonstrate better maintenance of CD-RISC scores at 9- and 15-months (follow-up) than those in the attention control arm (physical activity).
  - Participants in the RESILIENCE arm will demonstrate greater improvements in well-being (general health questionnaire) at 3-, 9-, and 15-months compared with those in the attention control arm.
  - The organizational support and resources of centers in both groups will improve significantly as assessed using the NIOSH worker well-being questionnaire (WellBQ).

9.2 SAMPLE SIZE DETERMINATION

The study is powered to detect a between group difference (Resilience group vs attention control) of overall resilience assets and resources as assessed with the CD-RISC (primary outcome). An effect size of at least 0.35 is expected, representing a clinical meaningful difference and a conservative estimate based off the SMART resilience program's well-documented efficacy to facilitate change across several mental health related outcomes (with effect sizes ranging from 0.3 to 1.1). Based on previous work we anticipate that, on average, about 8 workers will participate at each childcare center. Since this is a cluster randomized design, we assume that there will be an inherent ICC within centers. The ICCs observed in our previous work assessing childcare worker health outcomes ranged from 0.00 to 0.13.

Statistical Power

Centers per arm	Effect Size	ICC 0.00	ICC 0.03	ICC 0.10	ICC 0.13
30	0.30	0.91	0.84	0.67	0.60
30	0.35	0.97	0.93	0.80	0.73
30	0.40	0.99	0.98	0.89	0.84
35	0.30	0.94	0.89	0.73	0.67
35	0.35	0.99	0.96	0.85	0.80
35	0.40	1.00	0.99	0.93	0.89
40	0.30	0.97	0.92	0.79	0.73
40	0.35	0.99	0.98	**0.90**	0.85
40	0.40	1.00	0.99	0.96	0.93

Statistical power table describes the power for a range of ICCs, number of centers per arm, and effect sizes. Given these assumptions, a sample size of 80 centers (40 per arm), with an average of 8 workers per center, and an ICC ~0.10 would allow us to detect an effect size of 0.35 with 90% power at $\alpha=0.05$ level of significance. Even with a 15% loss to follow-up at 3 months (e.g., 12 centers or 96 workers), which is higher attrition than we expect based on our previous childcare-based trials, a completer's only analysis will still provide 84% power. An important secondary aim is to compare the maintenance of resilience interventions from 3 months to 15 months. Anticipating higher loss to follow-up at 15 months and assuming a minimal detectable effect size of at least 0.35, we retain 82% power with an ICC = 0.10 with 20% attrition (e.g., 16 centers or 128 workers). Missing data will be fully recovered as

DO NOT COPY

discussed in section 9.4.2 (below), which will remove or minimize (if present) confounding effects of missingness on our statistical power.

9.3 POPULATIONS FOR ANALYSES

Primary analyses will be performed using data from all randomized participants (ITT population). Additional assessments of primary endpoints will be completed for study completers (baseline and follow-up assessments) and on participants completing at least 50% of the assigned intervention tasks (i.e. lessons and webinars).

9.4 STATISTICAL ANALYSES

9.4.1 GENERAL APPROACH

Data analyses will be performed using SAS statistical software (Version 9.4 or higher, Cary, NC). Baseline descriptive characteristics will be summarized globally, and by arm, using frequencies and percentages for categorical variables, and means and standard deviations (or median [Q1, Q3] for non-symmetric data) for inherently quantitative variables. Prior to conducting data analyses, we will audit the data for completeness and quality, including missing data. We will evaluate distributions to ensure that they meet the assumptions of planned analyses (see 9.4.2 and 9.4.3), including the detection of outliers.

9.4.2 ANALYSIS OF THE PRIMARY ENDPOINT(S)

Our primary analyses will involve testing change in the total score from CD-RISC between SMART and control groups at 3 months. Using maximum likelihood methods, we will use multi-level linear mixed models (PROC MIXED) that include random effect for cluster to account for covariance between participants within the same center as well as fixed effects for time (0, 3, 9, 15 months), trial arm (SMART or matched attention control), time*arm interaction. To assess the primary endpoint from this mixed model we will include a contrast statement to calculate mean and 95% confidence intervals to estimate change for our primary outcome from baseline to 3- (primary endpoint), 9-, and 15-months (secondary endpoints). A proper error covariance structure will be chosen based on model fit indicated by model likelihood, Akaike Information Criterion and Bayesian Information Criterion. Residuals will be examined to check the assumptions of models. To further explore the effect of the intervention, sensitivity analyses will be conducted that adjust for baseline variables distributed differently between groups and to examine completers only. Similar analyses will be completed for secondary outcomes. Baseline variables including center and worker demographics will be included as covariates to assess their potential moderating effect

All analyses will be conducted using the intention-to-treat principle, in which all available data on all randomized participants are included. This approach minimizes bias if individuals drop out of the trial for different reasons. Every effort will be made to obtain follow-up data on all participants randomized, whether or not they follow their assigned treatment. Our maximum likelihood approach assumes that any missing outcome data are missing at random (i.e., missing data including that due to drop-out can be dependent on any previously observed outcomes or treatment assignment).[24] With this approach, we use all data that have been collected without regard to whether data are missing for a participant at another visit, including drop-out, and without explicit imputation of missing data. If the combined rate of missingness on variables is above 5%, we will impute missing endpoint data using multiple imputation

DO NOT COPY

techniques[25] and will assess the sensitivity of our results to various assumptions of missing data patterns.[26] The methodological literature currently recommends an inclusive analysis strategy that incorporates auxiliary variables into the missing data handling procedures because this approach can make the missing at random assumption more plausible and can improve statistical power.[27,28] All outcome variables and auxiliary variables (i.e., measured worker and center characteristics) will be incorporated into the imputation process.

9.4.3 ANALYSIS OF THE SECONDARY ENDPOINT(S)

Analysis of secondary end points will follow procedures described in section 9.4.2.

9.4.4 SAFETY ANALYSES

N/A

9.4.5 BASELINE DESCRIPTIVE STATISTICS

While randomization should be sufficient to balance baseline characteristics of the staff and directors recruited to each arm, we will compare age, race/ethnicity, weight, income, education, years working at ECE center, center size, and center cost (per week) between study arms using simple mean or frequency comparisons. If group differences are deemed statistically significant and clinically meaningful, we will fit models with and without covariates for assessment.

9.4.6 PLANNED INTERIM ANALYSES

No interim assessment of primary/secondary endpoint is planned.

9.4.7 SUB-GROUP ANALYSES

Exploratory analyses will include examination of the moderating effects of age, race/ethnicity, income, education, readiness for change, center size. Significant interactions between sub-group and study arm will be examined in more detail to summarize the relational impact (e.g. what age group shows most change) and meaningfulness (statistical and clinical significance) in greater detail.

9.4.8 TABULATION OF INDIVIDUAL PARTICIPANT DATA

Individual participant data will be compiled by measure and timepoint for analysis and summary statistics, but all reports and manuscripts will only include group level summaries of available data. After follow-up a public dataset will be made available. All identifiable information will be removed from public data.

9.4.9 EXPLORATORY ANALYSES

Exploratory analyses with follow protocols described in section 9.4.2. Exploratory models will include an additional interaction term to assess the moderating impact of:

- Age –
  - Continuous
  - Categorical (under 30, 30-55, 55+)
- Race/Ethnicity – based on previous work will likely be defined as:

DO NOT COPY

    - non-Hispanic White, non-Hispanic black, Hispanic
    - non-Hispanic white and BIPOC
- Income
    - 7-level “treated” as continuous
    - Median split
- Education: based on previous work will likely define as:
    - 2-level (high school/some college vs college degree+)
- Readiness for change
    - continuous score based on 20-Likert-type items related to intervention components

DO NOT COPY

10 SUPPORTING DOCUMENTATION AND OPERATIONAL CONSIDERATIONS

10.1 REGULATORY, ETHICAL, AND STUDY OVERSIGHT CONSIDERATIONS

10.1.1 INFORMED CONSENT PROCESS

10.1.1.1 CONSENT/ASSENT AND OTHER INFORMATIONAL DOCUMENTS PROVIDED TO PARTICIPANTS

The following consent materials are submitted with this protocol:

- Director consent
- Staff consent

10.1.1.2 CONSENT PROCEDURES AND DOCUMENTATION

Consent will be completed digitally through IRB approved Qualtrics survey. After screening, an information call, and verbal agreement participants will be sent a link to the online consent.

10.1.2 STUDY DISCONTINUATION AND CLOSURE

Given the minimal risk associated with this intervention study, it is highly unlikely that an accumulation of excess SAEs would prompt halting the trial. However, we will monitor SAE rates in all participants, with particular attention to those rated as severe and associated with study participation. If larger than expected SAE rates occur the DSMP, together with the MPIs, will alert the IRB and NCCIH that halting or modifications are under review by the study team.

10.1.3 CONFIDENTIALITY AND PRIVACY

Subject confidentiality is strictly held in trust by the investigators, study staff, and the sponsoring agency. This confidentiality is extended to any study information relating to subjects. The study monitor or other authorized representatives of the sponsor may inspect all study documents and records required to be maintained by the investigator, including but not limited to 0, 3, 9, and 15 month assessments for the study subjects. The study site and PIs will permit access to such records.

A number of steps will be taken to protect participants against any possible risks. Data will be collected by trained and experienced research assistants with clearly established data collection procedures. All study personnel will also be required to complete regular human subjects' protection certification as well as the good clinical practice for social/behavioral research certification available at the University of North Carolina, Chapel Hill (CITI training). Data collection trainings will reiterate the importance of protecting subjects against potential risks and maintaining confidentiality of all data.

Additionally, collection of data is for research purposes only, and is kept in strict confidence by study personnel. To help protect confidentiality, all participants will be assigned a numeric ID. All data collection tools will use that ID to minimize the appearance of identifiers (e.g., subject name) on paper forms and in electronic data tables. Hard copies of forms (if any) will be stored in a locked file cabinet at the UNC Center for Health Promotion and Disease Prevention.

DO NOT COPY

Electronic data files will be stored on UNC's secure password-protected servers. UNC's administration policies and programming coding standards are in compliance with current security regulations. To ensure data security of web-based data, the UNC network utilizes a secure authentication mechanism, the Transport Layer Security (TLS) system, which is the successor to the Secure Sockets Layer (SSL) protocol. This security mechanism supports encryption in both directions (to and from the website) and protects your username and password and also protects data with network encryption. Since TLS is web-based, it provides encryption of data across all devices including mobile devices such as smartphones.

10.1.4 FUTURE USE OF STORED SPECIMENS AND DATA

Public use study data and associated documentation will be deposited in the Research Data Management Core's UNC Dataverse. Dataverse is a digital archive for scholarly materials produced by members of the University of North Carolina at Chapel Hill community. Dataverse provides searchable study-level metadata for dataset discovery. Dataverse assigns DOIs as persistent identifiers and has a robust preservation plan to ensure long-term access. Data will be discoverable online through standard web search of the study-level metadata as well as the persistent pointer from the DOI to the dataset.

Cleaned data used to assess study aims from this project will be made available as soon as possible, and no later than the time of publication or the end of the funding period, whichever comes first. The duration of preservation and sharing of the data will be a minimum of 10 years after the funding period.

There are no anticipated factors or limitations that will affect the access, distribution or reuse of the survey data generated by the proposal. Upon the conclusion of data collection for this study, all direct respondent identifiers (e.g., names and addresses) will be expunged and securely maintained in a separate control file for future contact purposes. Access to participants' identifying information is restricted to approved staff solely as part of their project duties within a secure computing environment. Deidentification will be finalized by the end of data processing, preceding the public release.

10.1.5 KEY ROLES AND STUDY GOVERNANCE

ROLE	NAME	DEGREE	LOCATION	Address	Email
MPI	Debra Jones	PhD	UNC-CH	[redacted]	djjones@email.unc.edu
MPI	Derek Hales	PhD	UNC-CH	[redacted]	derekh@email.unc.edu
				[redacted]	
DSMB	Jimikaye Courtney	PhD	UNC-CH	[redacted]	jimikaye@unc.edu
DSMB	Neal Montgomery	PhD	U Kansas Medical	[redacted]	rmontgomery@kumc.edu
DSMB	Katie Lenger	PhD	UNC-CH	[redacted]	Katherine_Lenger@med.unc.edu

10.1.6 SAFETY OVERSIGHT

The Independent Monitoring Committee (IMC) for this study is comprised of Dr. Jimikaye Courtney, Dr. Neal Montgomery, and Dr. Katie Lenger. The members of the IMC are not part of the key personnel involved in this grant and work independently of the PIs, Drs. Deborah Jones and Derek Hales. No member of the Committee has collaborated or co-published with the PIs within the past three years. Dr. Courtney has experience conducting physical activity intervention in adults. Dr. Montgomery is a PhD prepared

DO NOT COPY

biostatician and has previously served on an IMC (DSMB). Dr. Lenger has experience in mindfulness research and is a clinical psychologist.

Progress and safety will be reviewed monthly, with reports, including summaries of recruitment, Adverse Events, withdrawal, and attrition. These reports will be provided to the IMC quarterly. An annual report will be compiled and will include a list and summary of AEs. In addition, the annual report will address (1) whether AE rates are consistent with pre-study assumptions; (2) reasons for dropouts; (3) whether all participants met eligibility criteria; (4) whether continuation of the study is justified based on current enrollment and withdrawal rates; and (5) conditions whereby the study might be terminated prematurely. The annual report will be sent to the Independent Monitor(s) and will be forwarded to the IRB and NCCIH. The IRB and other applicable recipients will review progress of this study on an annual basis.

10.1.7 CLINICAL MONITORING

N/A (see 10.1.8 Quality Assurance and Quality Control)

10.1.8 QUALITY ASSURANCE AND QUALITY CONTROL

Quality control (QC) procedures will be implemented as follows:

Informed consent

- Study staff will review both the documentation of the consenting process as well as a percentage of the completed consent documents. This review will evaluate compliance with GCP, accuracy, and completeness. Feedback will be provided to the study team to ensure proper consenting procedures are followed.

Source documents and electronic data

- Data will be initially captured on source documents (see Section 10.1.9, Data Handling and Record Keeping) and will ultimately be entered into the study database. Data will be initially captured on source documents (see Section 10.1.9, Data Handling and Record Keeping) and will ultimately be entered into the study database. To ensure accuracy, site staff will compare a representative sample of source data against the database, targeting key data points in that review.

Intervention Fidelity

- Consistent delivery of the study interventions will be monitored throughout the intervention phase of the study. Procedures for ensuring fidelity of intervention delivery are described in Section 6.2.1, Interventionist Training and Tracking.

Protocol Deviations

- The study team will review protocol deviations on a regular basis and will implement corrective actions when the quantity or nature of deviations are deemed to be at a level of concern. Should independent monitoring become necessary, the PI will provide direct access to all trial related sites, source data/documents, and reports for the purpose of monitoring and auditing by the sponsor/funding agency, and inspection by local and regulatory authorities.]

Our quality control and assurance plans fall into the following categories:
Staff training

DO NOT COPY

- In year 1, study staff will be trained to manage the delivery of the SMART program and attention control physical activity program. A primary and back-up member of the research staff will complete all trainings. Staff manuals, which outline protocols and procedures for intervention delivery, will be developed for each intervention arm as we have done in our previous trials.
- For SMART, research staff will complete the Trainer Skills Intensive program (~6 months), conducted by Drs. Chesak (Co-I) and Sood (collaborator), to become a certified resilience trainer. This standardized program provides the knowledge and skills of the science and art of resilience. The Certified Resilience Training Program has successfully trained over 150 resilience trainers.
- For the attention control, similar to our prior studies using physical activity education interventions, a detailed manual of procedures for the physical activity intervention will be provided to research staff that contains descriptions of all intervention components. Staff will receive standard training and certification which includes a mix of written materials, didactic sessions with the investigative team, and hands-on/practice-based exercises (~6 months).

Subject Accrual

- The MPIs, project manager, and data manager will meet on a biweekly basis throughout the study. During all active recruitment periods, time will be dedicated to the review subject accrual. Recruitment for efficacy trial will start during the last quarter of Year 1. Each recruitment period is expected to last 3 months. During these recruitment periods, the project manager and data manager will create and maintain recruitment and participant tracking spreadsheets that will allow for the generation of reports summarizing the number of centers identified for recruitment, the number of centers/directors with confirmed eligibility and interest, number of staff with confirmed eligibility and interest, scheduled/completed recruitment visits, any deviations to anticipated cluster size (number of workers per center with consent), etc. These reports will allow for easy comparison of enrollment numbers for centers, directors, and staff against target enrollment numbers to assess progress.

Adherence to Study Protocols

- During biweekly meetings, the project manager and data manager will also report on study protocols and any adherence questions or issues. The content of these reports will vary depending on the current study activities. During recruitment, the tracking database will provide the data needed to generate reports on adherence to recruitment protocols. During implementation, the project manager will create reports summarizing the process data collected and completion of core components of each study group. During measurement, the project manager and data manager will maintain a measurement activity spreadsheet and issue log that will allow for the generation of reports summarizing scheduled/completed measurement visits, any questions or issues encountered during scheduling, completeness of the data collected, and any issues with missing data (e.g., incomplete online surveys).

Withdrawals

- The project manager will monitor for all requested withdrawals that may come from centers, directors, or staff that may occur at any time throughout the study. When withdrawal requests are received, the project manager will work to confirm with the participant his/her desire to officially withdraw and record any reason he/she is willing to provide. Withdrawals will be logged and reported back to the MPIs and study statistician during biweekly meetings.

Data Quality and Management:

DO NOT COPY

- The infrastructure for the data collection and management proposed for this project have been successfully implemented by our team over the last few years. Data collection systems (Qualtrics, REDCap) are programed with appropriate data validation checks, response requirement (where needed), and several "data quality check" items. Additionally, the project manager and data manager will work together to monitor all data streams, including survey completion and conduct initial data quality assessments (i.e., number of missing responses, straight line responding, short survey completion times). If data quality for a participant is judged to be low during initial assessments, the participants will be asked to complete surveys a second time. If a participant is missing needed items, they will be contacted by email to collect this information (e.g., missing single CD-RISC item, education level). Data cleaning and scoring will follow the established protocol for all surveys selected (e.g., CD-RISC). The project manager will lead the compilation of process data, using it to create implementation reports that are reviewed regularly during biweekly meetings. The quality of data collected via online surveys will be regularly reviewed by the data manager to check for completeness. The project manager will be engaged immediately if follow-up is needed to address any errors or missing data. After each measurement period, the project manager and data manager will clean and manage the data (e.g., apply data labels, examine distributions, review data for extreme values, create and check derived variables, create and save datasets, and develop a data user manual) under the direction and guidance of the study statistician. The project manager and data manager will report on the progress of their activities during biweekly meetings.

10.1.9 DATA HANDLING AND RECORD KEEPING

10.1.9.1 DATA COLLECTION AND MANAGEMENT RESPONSIBILITIES

Data collection will be conducted through online survey with a small amount of contact and screening information collected during screening calls with center directors. The Qualtrics survey platform will be used for all online data collection. This system includes password protection and internal quality checks, such as skip patterns, automatic range limits, and randomization. Data will be examined 1-2 times per quarter to identify values that appear inconsistent, incomplete, or inaccurate.

10.1.9.2 STUDY RECORDS RETENTION

Cleaned data used to assess study aims from this project will be made available as soon as possible, and no later than the time of publication or the end of the funding period, whichever comes first. The duration of preservation and sharing of the data will be a minimum of 10 years after the funding period.

10.1.10 PROTOCOL DEVIATIONS

A protocol deviation is defined as any noncompliance with the clinical trial protocol or this submitted protocol document. Deviations will be classified as “minor” (small changes/events affecting a few people or modifications to intervention with minimal impacts) or “significant” (major changes to intervention delivery, data collection, outcomes, or aims). The noncompliance may be either on the part of the participant, the investigator, or the study site staff. As a result of deviations, corrective actions will be developed by the site and implemented promptly. It will be the responsibility of the site investigator to use continuous vigilance to identify and report deviations in a timely manner. All deviations will be

DO NOT COPY

addressed in study source documents and reports to the Program Official and DSMB. Protocol deviations will be sent to the UNC IRB per policy.

10.1.11 PUBLICATION AND DATA SHARING POLICY

This study will be conducted in accordance with the publication and data sharing policies and regulations set forth by NIH, which ensures that the public has access to the published results of NIH funded research. This study will comply with the NIH Data Sharing Policy and Policy on the Dissemination of NIH-Funded Clinical Trial Information and the Clinical Trials Registration and Results Information Submission rule. As such, this trial is registered at ClinicalTrials.gov, and results will be submitted for public access for all registered primary and secondary outcomes within 6 months of study completion. In addition, every attempt will be made to publish results in peer-reviewed journals. Data from this study may be requested from other researchers. Considerations for ensuring confidentiality of these shared data are described in Section 10.1.3.

10.1.12 CONFLICT OF INTEREST POLICY

The independence of this study from any actual or perceived influence is critical. Our goal is to disclose and manage any actual conflict of interest (COI) for those who have a role in the design, implementation, analysis, publication, or any aspect of this trial. Persons who have a perceived COI will be required to have such conflicts managed in a way that is appropriate to their participation. Currently one collaborator has ownership of the lessons and materials used for some of the resilience arm of the study. This person provides training and access to a website used to deliver intervention material to participants (text and recorded video lessons) but has no oversite or decision-making tasks for the full intervention design or delivery. He is also not permitted access to data and has no analysis or reporting responsibilities. As per IRB, all study personnel are required to regularly complete a COI disclosure form which will help the study leadership track and manage all any actual or perceived influence. All study reports and manuscripts authored by personnel with an established COI will include statements disclosing the conflict.

10.2 ADDITIONAL CONSIDERATIONS

N/A

10.3 ABBREVIATIONS AND SPECIAL TERMS

AE	Adverse Event
CFR	Code of Federal Regulations
CMP	Clinical Monitoring Plan
COC	Certificate of Confidentiality
COI	Conflict of Interest
CONSORT	Consolidated Standards of Reporting Trials
DCC	Data Coordinating Center
DHHS	Department of Health and Human Services
DSMB	Data Safety Monitoring Board
EC	Ethics Committee
ECE	Early Care and Education
FFR	Federal Financial Report

DO NOT COPY

GCP	Good Clinical Practice
ICH	International Council on Harmonisation
IRB	Institutional Review Board
ISM	Independent Safety Monitor
ITT	Intention-To-Treat
MOP	Manual of Procedures
NCT	National Clinical Trial
NIH	National Institutes of Health
NIH IC	NIH Institute or Center
OHRP	Office for Human Research Protections
PA	Physical Activity
PI	Principal Investigator
QA	Quality Assurance
QC	Quality Control
SAE	Serious Adverse Event
SAP	Statistical Analysis Plan
SMC	Safety Monitoring Committee
SOP	Standard Operating Procedure
UP	Unanticipated Problem
US	United States

DO NOT COPY

10.4 PROTOCOL AMENDMENT HISTORY

Version	Date	Description of Change	Brief Rationale
1.0	05.06.2025	Original submission	Required

DO NOT COPY

11 REFERENCES

1. Chmitorz A, Kunzler A, Helmreich I, et al. Intervention studies to foster resilience - A systematic review and proposal for a resilience framework in future intervention studies. Clin Psychol Rev. 2018;59:78-100.
2. Robertson IT, Cooper CL, Sarkar M, Curran T. Resilience training inthe workplace from 2003 to 2014: A systematic review. J Occup Organ Psychol. 2015;88:533-562.
3. Vanhove AJ, Herian M, Perez ALU, Harms PD, Lester PB. Can resilience be developed at work? A meta-analytic review of resilience-building programme effectiveness. J Occup Organ Psychol. 2015:1-30.
4. Joyce S, Shand F, Tighe J, Laurent SJ, Bryant RA, Harvey SB. Road to resilience: a systematic review and meta-analysis of resilience training programmes and interventions. BMJ Open. 2018;8(6):e017858.
5. Leppin AL, Bora PR, Tilburt JC, et al. The efficacy of resiliency training programs: a systematic review and meta-analysis of randomized trials. PLoS One. 2014;9(10):e111420.
6. Macedo T, Wilheim L, Goncalves R, et al. Building resilience for future adversity: a systematic review of interventions in non-clinical samples of adults. BMC Psychiatry. 2014;14:227.
7. Ferreira M, Marques A, Gomes PV. Individual Resilience Interventions: A Systematic Review in Adult Population Samples over the Last Decade. International journal of environmental research and public health. 2021;18(14):7564.
8. Linnan L, Arandia G, Bateman LA, Vaughn A, Smith N, Ward D. The Health and Working Conditions of Women Employed in Child Care. Int J Environ Res Public Health. 2017;14(3).
9. U.S. Bureau of Labor Statistics. Occupational Outlook Handbook. https://www.bls.gov/ooh/home.htm. Accessed Oct 8, 2020.
10. Brody DJ, Pratt LA, Hughes JP. Prevalence of Depression Among Adults Aged 20 and Over: United States, 2013-2016. NCHS data brief. 2018(303):1-8.
11. Connor KM, Davidson JR. Development of a new resilience scale: the Connor-Davidson Resilience Scale (CD-RISC). Depress Anxiety. 2003;18(2):76-82.
12. Sood A, Prasad K, Schroeder D, Varkey P. Stress management and resilience training among Department of Medicine faculty: a pilot randomized clinical trial. J Gen Intern Med. 2011;26(8):858-861.
13. Magtibay DL, Chesak SS, Coughlin K, Sood A. Decreasing Stress and Burnout in Nurses: Efficacy of Blended Learning With Stress Management and Resilience Training Program. J Nurs Adm. 2017;47(7-8):391-395.
14. Chesak SS, Khalsa TK, Bhagra A, Jenkins SM, Bauer BA, Sood A. Stress Management and Resiliency Training for public school teachers and staff: A novel intervention to enhance resilience and positively impact student interactions. Complement Ther Clin Pract. 2019;37:32-38.
15. MacCoon DG, Imel ZE, Rosenkranz MA, et al. The validation of an active control intervention for Mindfulness Based Stress Reduction (MBSR). Behaviour research and therapy. 2012;50(1):3-12.
16. Bay E, Chan RR. Mindfulness-based versus health promotion group therapy after traumatic brain injury. Journal of psychosocial nursing and mental health services. 2019;57(1):26-33.
17. Ward DS, Vaughn AE, Hales D, et al. Workplace health and safety intervention for child care staff: Rationale, design, and baseline results from the CARE cluster randomized control trial. Contemp Clin Trials. 2018;68:116-126.
18. Linnan LA, Vaughn AE, Smith FT, et al. Results of caring and reaching for health (CARE): a cluster-randomized controlled trial assessing a worksite wellness intervention for child care staff. Int J Behav Nutr Phys Act. 2020;17(1):64.
19. Ward DS, Vaughn AE, Burney RV, et al. Keys to healthy family child care homes: Results from a cluster randomized trial. Prev Med. 2020;132:105974.

DO NOT COPY

20. Willis EA, Szabo-Reed AN, Ptomey LT, et al. Distance learning strategies for weight management utilizing online social networks versus group phone conference call. Obes Sci Pract. 2017;3(2):134-142.
21. Hanson P, Vaughn A, Mazzucca S, Erinosho T, Ward DS. Getting Healthy for the Holidays: Results from a worksite wellness intervention for childcare center staff in North Carolina. International Society for Behavioral Nutrition and Physical Activity; May 23-26, 2012, 2012; Austin, TX.
22. Willis EA, Szabo-Reed AN, Ptomey LT, et al. Distance learning strategies for weight management utilizing social media: A comparison of phone conference call versus social media platform. Rationale and design for a randomized study. Contemporary clinical trials. 2016;47:282-288.
23. Østbye T, Mann CM, Vaughn AE, et al. The keys to healthy family child care homes intervention: study design and rationale. Contemporary clinical trials. 2015;40:81-89.
24. Little RJ. Modeling the drop-out mechanism in repeated-measures studies. Journal of the american statistical association. 1995;90(431):1112-1121.
25. Little RJ, Rubin DB. Statistical analysis with missing data. Vol 793: John Wiley & Sons; 2019.
26. Molenberghs G, Kenward M. Missing data in clinical studies. Vol 61: John Wiley & Sons; 2007.
27. Schafer JL, Graham JW. Missing data: our view of the state of the art. Psychological methods. 2002;7(2):147.
28. Collins LM, Schafer JL, Kam C-M. A comparison of inclusive and restrictive strategies in modern missing data procedures. Psychological methods. 2001;6(4):330.

DO NOT COPY
