# Supplementary material for: Protocol for a cluster-randomized control trial of a remote workplace resilience intervention for early care and education providers: The OnWARD trial
Source: PLoS One. 2026 Mar 13;21(3):e0340915. doi: 10.1371/journal.pone.0340915 (PMC12987477; doi:10.1371/journal.pone.0340915)
Supplement: S2 File — (PDF) [file pone.0340915.s002.pdf]

OnWARD Surveys

Staff Surveys

Thank you for your interest and willingness to participate.
For all of our programs we have the people participating answer some questions at the beginning of the study to help us describe and understand child care providers.

- *The information provided is only used to describe the group of centers and staff participating in the project, not individual centers or people. In research reports only summaries for the group as a whole (e.g. averages, % of all people) are presented.*

As a childcare worker, your responses, opinions, and ongoing participation will help us evaluate the exciting new OnWARD program.

We appreciate your help!

Personal Demographics

The next few questions ask a little about you.

TchDem_position
What is your main job at this child care program (center)?
- ○ Lead Teacher (1)
- ○ Assistant Teacher (2)
- ○ Classroom Aide or Floater (3)
- ○ Cook/Food Service (4)
- ○ Office/Adminstrative staff (5)
- ○ Assistant Director (6)
- ○ Director (7)
- ○ Other (specify) (8) ____

TchDem_PosYear
About how long have you worked as ${TchDem_position/ChoiceGroup/SelectedChoices} for this child care program?
- ○ DROPDOWN
  - i) Less than 3 months TO 20 years or more

TchDem_CntWrkYrs
About how long have you worked at this child care program?
(working in any position at the center)
- ○ DROPDOWN
  - i) Less than 3 months TO 20 years or more

TchDem_wrkFull
Do you currently work full-time or part-time at this child care program?
- ○ Part-Time (less than 30 hours per week) (1)
- ○ Full-Time (30 or more hours per week) (2)

TchDem_WrkHours
About how many hours do you work for this center during an average week?
- ○ DROPDOWN
  - i) less than 5 hours per week TO 65 or more hours per week

TchDem_edu

What is the highest level of school you have completed?
- ○ Middle School (less than 9th grade) (1)
- ○ Some High School (2)
- ○ High School diploma or equivalent (GED) (3)
- ○ Some College (no degree) (4)
- ○ Associate Degree (2-year) (5)
- ○ Bachelor's Degree (4-year) (6)
- ○ Masters/Doctoral/Professional Degree (7)
- ○ Prefer not to answer (77)

TchDem_CEtrain
Have you completed any training related to early childhood education through a community college, university, or other accredited training program?
- ○ Yes (1)
- ○ No (0)

Display this question:
If Have you completed any training related to early childhood education through a community college,... = Yes
And If
What is the highest level of school you have completed? = Associate Degree (2-year)
Or What is the highest level of school you have completed? = Bachelor's Degree (4-year)
Or What is the highest level of school you have completed? = Masters/Doctoral/Professional Degree
Or What is the highest level of school you have completed? = Prefer not to answer

TchDem_CEdegree
Do you have a degree, or certificate, in child development or teaching?
- ○ Yes (1)
- ○ No (0)

TchDem_Age
What is your age? (in years)

____

TchDem_sex1
Which of the following describes you?
- ○ Male (1)
- ○ Female (2)
- ○ Provide other options --> (3)

Display this question:
If Which of the following describes you? = Provide other options -->

TchDem_sex2
Which of the following describes you?
[mark all that apply]
- ▢ Female (1)
- ▢ Male (2)
- ▢ Non-binary (4)
- ▢ Transgender (5)
- ▢ Cisgender (6)
- ▢ Agender (7)
- ▢ Gender queer (8)
- ▢ Self-Describe (specify) (9) ____
- ▢ ⊗Prefer no response (77)

TchDem_livsit

Which of the following best describes your current living situation?

- ○ Living alone (i.e. no other adult) (1)
- ○ Living with parent(s) or roommate(s) (2)
- ○ Living with a partner/spouse (3)
- ○ Living temporarily with a relative/friend (short term) (4)
- ○ Self Describe (5) ____

TchDem_adlt

Including yourself, how many adults live in your household? *(Adult is anyone 18+ years who helps with household expenses or duties)*

- ○ 1 (just me) (1)
- ○ 2 adults (2)
- ○ 3 adults (3)
- ○ 4 or more adults (4)

TchDem_Nchild

How many children (under 18 years) live in your household?

- ○ 0 (no children) (0)
- ○ 1 child (1)
- ○ 2 children (2)
- ○ 3 children (3)
- ○ 4 or more children (4)

TchDem_Eng

Are you fluent in a language other than English? *(e.g. Spanish, ASL, Chinese, German)*

- ○ YES (1)
- ○ NO (0)

Display this question:

If Are you fluent in a language other than English? (e.g. Spanish, ASL, Chinese, German) = YES

TchDem_Lang

Which language(s), other than English, do you speak fluently? *[Mark all that apply]*

- ▢ Spanish (1)
- ▢ American Sign language (hearing impaired) (2)
- ▢ Chinese (Mandarin, Cantonese, Hokkien and other varieties) (3)
- ▢ Tagalog (including Filipino) (4)
- ▢ Vietnamese (5)
- ▢ French (6)
- ▢ Arabic (7)
- ▢ Korean (8)
- ▢ Other (specify) (9) ____

TchDem_race

Which of these race/ethnicity options describes you? *[Mark all that apply]*

- ▢ American Indian or Alaska Native (1)
- ▢ Asian (including India) (2)
- ▢ Black or African-American (3)
- ▢ Hispanic, Latino/a/x, or Spanish origin (4)
- ▢ Native Hawaiian or Other Pacific Islander (5)
- ▢ White (6)
- ▢ Self-Describe (specify) (7) ____
- ▢ ⊗Prefer not to answer (77)

Display this question:

If Which of these race/ethnicity options describes you? [Mark all that apply] != Hispanic, Latino/a/x, or Spanish origin

TchDem_Ethn

Do you consider yourself of Hispanic, Latino/a/x, or Spanish origin?

- ○ Yes, Hispanic, Latino/a/x, or Spanish ethnicity (1)
- ○ No, Non-Hispanic (2)
- ○ Prefer not to answer (3)

Display this question:
If Which of these race/ethnicity options describes you? [Mark all that apply] = Hispanic, Latino/a/x, or Spanish origin
Or Do you consider yourself of Hispanic, Latino/a/x, or Spanish origin? = Yes, Hispanic, Latino/a/x, or Spanish ethnicity

TchDem_Hisp

Which of these options describes you? *[Mark all that apply]*

- ▢ Cuban (1)
- ▢ Mexican, Mexican-American, Chicano/a/x (2)
- ▢ Central-American (3)
- ▢ Spanish (4)
- ▢ Puerto Rican (5)
- ▢ Self-Describe (specify) (6) ____
- ▢ ⊗Prefer not to answer (77)

TchDem_income

Please provide an estimate of the total annual income for your household? includes income from all adults contributing to household expenses

- ○ under $25,000 per year (25000)
- ○ $25,000 to $34,999 per year (30000)
- ○ $35,000 to $44,999 per year (40000)
- ○ $45,000 to $54,999 per year (50000)
- ○ $55,000 to $64,999 per year (60000)
- ○ $65,000 to $74,999 per year (70000)
- ○ $75,000 to $84,999 per year (80000)
- ○ $85,000 to $99,999 per year (90000)
- ○ $100,000 or more per year (100000)
- ○ Prefer not to answer (77)

TchDem_preg

Are you currently pregnant?

- Why we ask...
  - People who are pregnant can participate.
  - We do not disclose who might be pregnant, but we are required by funding agency to report the number, or percent, of people in a few broad categories in progress reports a few times each year (e.g. # by age group [20-30yrs], % who might be pregnant). Example. Of the 200 people currently enrolled in the program 3% report being pregnant.

- ○ Yes (1)
- ○ No (0)

TchWtHis_Height

How tall are you without shoes? (current height)

- ○ DROP down list from [under 4 ft] to [7 ft or taller]

TchWtHis_Weight

About how much do you currently weigh without shoes (in pounds)? (number only)

____

HealthInsur

What type of health insurance do you currently have?

- ○ I currently DO NOT have health insurance (1)

- Health insurance through my employer (this childcare center) (2)
- Health insurance through spouse's employer (3)
- Medicare (4)
- Medicaid (5)
- Health insurance through HealthCare.gov or other insurance marketplace (6)
- Other (specify) (7) ____

DentalInsur
Do you currently have insurance that covers dental care?

- Yes (1)
- No (0)

TchBehBL01
These questions ask about programs, behaviors, or therapies you may be currently involved with. We are asking about things you currently do.   **Are you currently...**

	**NO** (0)	**YES** (1)
...enrolled in a stress management or meditation program? (TchBehBL01_1)	○	○
...enrolled in an exercise program or regular exercise class? *(gym, fitness center, recreation dept, community center)* (TchBehBL01_2)	○	○
... working with a nutritionist or dietician to improve your diet? (TchBehBL01_3)	○	○
...working with an exercise trainer or physical therapist to increase your activity level? (TchBehBL01_4)	○	○
...going to individual, or group, therapy or working with a mental health provider or employee assistance program, for counseling? (TchBehBL01_5)	○	○

Display this question:
If These questions ask about programs, behaviors, or therapies you may be currently involved with. W... = ...enrolled in a stress management or meditation program? [YES]

TchBehBLstress
About how long have you been enrolled in a meditation or stress management program?

- Less than 1 week (1)
- 1-2 weeks (2)
- 1 month (4)
- 2 months (8)
- 3 months (12)
- 4 months (16)
- 5 months (20)
- 6 months or more (24)

Display this question:
If These questions ask about programs, behaviors, or therapies you may be currently involved with. W... = ...enrolled in an exercise program or regular exercise class? (gym, fitness center, recreation dept, community center) [YES]

TchBehBLexProg
About how long have you been regularly participating in exercises classes or programs?

- Less than 1 week (1)
- 1-2 weeks (2)
- 1 month (4)
- 2 months (8)
- 3 months (12)
- 4 months (16)
- 5 months (20)
- 6 months or more (24)

Display this question:
If These questions ask about programs, behaviors, or therapies you may be currently involved with. W... = ... working with a nutritionist or dietician to improve your diet? [YES]

TchBehBLnutr

About how long have you been following the guidance of the nutritionist or dietician?

- ○ Less than 1 week (1)
- ○ 1-2 weeks (2)
- ○ 1 month (4)
- ○ 2 months (8)
- ○ 3 months (12)
- ○ 4 months (16)
- ○ 5 months (20)
- ○ 6 months or more (24)

Display this question:

If These questions ask about programs, behaviors, or therapies you may be currently involved with. W... = ...working with an exercise trainer or physical therapist to increase your activity level? [YES]

TchBehBLexTrain

About how long have you been following the guidance of an exercise trainer or physical therapist?

- ○ Less than 1 week (1)
- ○ 1-2 weeks (2)
- ○ 1 month (4)
- ○ 2 months (8)
- ○ 3 months (12)
- ○ 4 months (16)
- ○ 5 months (20)
- ○ 6 months or more (24)

Display this question:

If These questions ask about programs, behaviors, or therapies you may be currently involved with. W... = ...going to individual, or group, therapy or working with a mental health provider or employee assistance program, for counseling? [YES]

TchBehBLmenhealth

About how long have you been following the guidance of a therapist or mental health counselor?

- ○ Less than 1 week (1)
- ○ 1-2 weeks (2)
- ○ 1 month (4)
- ○ 2 months (8)
- ○ 3 months (12)
- ○ 4 months (16)
- ○ 5 months (20)
- ○ 6 months or more (24)

Display this question:

If Please provide an estimate of the total annual income for your household? includes income from al... = Prefer not to answer

TchDem_incMed56

Is your current total household income more than $55,000 per year?

- ○ Yes (1)
- ○ No (0)

Display this question:

If Please provide an estimate of the total annual income for your household? includes income from al... = Prefer not to answer

And Is your current total household income more than $55,000 per year? = No

TchDem_incMed35

Is your current income more than $35,000 per year?

- ○ Yes (1)
- ○ No (0)

Class Information

The next few questions ask a little about your classroom at the center. *If you work in more than one classroom think about the room where you spend most of your time.*

TchCls_numChld

How many children are currently enrolled in your classroom at this center? *if you work in more than one classroom, answer based on the classroom where you have spent most time during the past few weeks.*

____

TchCls_AgeGrp

What age children are in your classroom? *[mark all that apply]*

- 5 month olds and younger (1)
- 6 - 11 month olds (2)
- 1 year olds (3)
- 2 year olds (4)
- 3 year olds (5)
- 4 year olds (6)
- 5 year olds (7)
- 6 year olds and older (8)

TchCls_NumStaff

Including yourself, how many teachers, assistants, or aides/floaters work in your classroom during inside time on most days? example during inside circle, center, or group activity times

- (1)
- (2)
- (3)
- or more (4)

TchCls_progs

Is your classroom currently using any of these health and wellness programs or curricula? *[Mark all that apply]*

- Color Me Healthy (1)
- Healthy Kids, Healthy Future (2)
- Let's Move Child Care (3)
- I am Moving I am Learning (4)
- The Incredible Years (5)
- Growing Minds (6)
- Rainbow in My Tummy (7)
- Go NAPSACC (8)
- CASEL (social emotional learning) (9)
- Changing Perspectives (social emotional program) (10)
- Other physical activity, motor skills, or social emotional wellness curriculum *[Specify]* (11) ________________
- ⊗None of the above (12)

TchCls_Eng

Do any children in your classroom speak a language other than English as their primary means of communication? *(e.g. Spanish, ASL, Chinese, German)*

- YES (1)
- NO (0)

Display this question:

If Do any children in your classroom speak a language other than English as their primary means of c... = YES

TchCls_Lang

Which languages, other than English, do these children usually speak? *[Mark all that apply]*

- Spanish (1)
- American Sign language (hearing impaired) (2)
- Chinese (Mandarin, Cantonese, Hokkien and other varieties) (3)
- Tagalog (including Filipino) (4)
- Vietnamese (5)
- French (6)
- Arabic (7)
- Korean (8)
- Other (specify) (9) ____

ClsDSenroll

Does your classroom currently have any enrolled children with a Down Syndrome or Autism Spectrum Disorder diagnosis?

- YES (1)
- NO (0)

TchCntHealthProm

During the last 12 months has the center where you work offered, or promoted, any type of heath or wellness program for staff?

- Yes (1)
- No (0)

CD-RISC

We are administering CDRISC-25. This survey requires small fee for use and cannot be reproduced here. If interested, please contact survey creator at https://www.cd-risc.com/index.php

Physical Activity

As you answer the next few questions think about the exercise and other physical activities you did over the past week (last 7 days). We will ask you to report activities in two broad categories:

- Vigorous intensity - you are breathing hard and fast, and your heart rate has gone up quite a bit, like jogging.
- Moderate intensity - you are working hard enough to increase your heart rate and breathing some, like walking.

PAvigIntro

First, we will ask about physically strenuous, or vigorous, physical activity you did outside of work during the last week. Moderate intensity activities will be reported in the next question.

- DO NOT count job-related activities or things done as part of your job at the child care program.
- Vigorous activities feel as intense as jogging. Usually, you are breathing hard and fast, and your heart rate has gone up quite a bit.
  - *Examples might be: Zumba, jogging, high intensity exercise class, HITT workout, fast biking, swimming laps, basketball, soccer, jumping rope, heavy weight lifting.*

PAvigDays

Outside of job-related work, **How many days last week did you participate in strenuous, or vigorous intensity, physical activity?**

- 0 days (0) to 7 days (7)

Display this question:
If Outside of job-related work, How many days last week did you participate in strenuous, or vigorou... != 0 days

PAvigMinPerDay

On the ${PAvigDays/ChoiceGroup/SelectedChoices} days you did vigorous activity last week, about how many minutes per day did you do? *if the amount of time each day differs, try to report average day.*

- Less than 5 min per day (3)
- 5 minutes per day (5)
- 10 minutes per day (10)
- 15 minutes per day (15)
- 20 minutes per day (20)
- 30 minutes per day (30)
- 45 minutes per day (45)
- 60 minutes per day (60)
- 75 minutes per day (75)
- 90 minutes per day (90)
- 120 minutes (2 hours) per day or more (120)

PAmodIntro

Now, we will ask about the moderate intensity physical activity you did outside of work during the last week.

- DO NOT count job-related activities or things done as part of your job at the child care program.
- Moderate Activities feel as intense as walking at a normal pace. When doing them you are working hard enough to increase your heart rate and breathing.
  - *Examples might be: Walking briskly, water aerobics, biking slower than 10 MPH, pushing lawn mower, softball, general gardening, light weight lifting.*

PAmodDays

Outside of job-related work, **How many days last week did you participate in moderate intensity physical activity?**

- 0 days (0) to 7 days (7)

Display this question:
If Outside of job-related work, How many days last week did you participate in moderate intensity ph... != 0 days

PAmodMinPerDay

On the ${PAmodDays/ChoiceGroup/SelectedChoices} days you did moderate intensity physical activity last week,

about how many minutes per day did you do? *if the amount of time each day differs, try to report average day.*

- ○ Less than 5 min per day (3)
- ○ 5 minutes per day (5)
- ○ 10 minutes per day (10)
- ○ 15 minutes per day (15)
- ○ 20 minutes per day (20)
- ○ 30 minutes per day (30)
- ○ 45 minutes per day (45)
- ○ 60 minutes per day (60)
- ○ 75 minutes per day (75)
- ○ 90 minutes per day (90)
- ○ 120 minutes (2 hours) per day or more (120)

PAstrIntro

Next, we will ask about muscle strengthening exercise.

- You likely included this type of exercise in your moderate or vigorous activity already reported.
- *Muscle strengthening activities include things like lifting weights, using resistance bands, or doing exercises that use your body weight for resistance (example: push-ups, sit-ups, Yoga, etc.)*

PAstrDays

Outside of job-related work, **How many days last week did you do exercise that included muscle strengthening activities?**

- ○ 0 days (0) to 7 days (7)

Display this question:
If Outside of job-related work, How many days last week did you do exercise that included muscle str... != 0 days

STR_type

What type of muscle strength activities did you do last week? *[Mark all that apply]*

- ☐ ⊗NO strength training activities this week (6)
- ☐ Lifting Weights (free weights or machines) (1)
- ☐ Workout with Resistance Bands (2)
- ☐ Body weight strength exercises (push-ups, pull-ups, burpee) (3)
- ☐ Class/Group Workout with Muscle Strength Focus (like Muscle Pump, HiiT) (4)
- ☐ Other (5) ____

PA_rating

How would you describe your level of physical activity and exercise last week?

- ○ A lot less Active than normal (1)
- ○ Less Active than normal (2)
- ○ Activity level same as usual this week (3)
- ○ More Active than normal (4)
- ○ A lot More Active than normal (5)

Readiness for Action

The next few items ask about things people sometimes do, or change. For each, think about how likely you are to do the behavior over the next 3 months.

	Over the next 3 months, how likely are you to...	*I will Not do (1)*	*Very Unlikely (2)*	*Unlikely (3)*	*Somewhat Unlikely (4)*	*Somewhat Likely (5)*	*Likely (6)*	*Very Likely (7)*
TchRdy13c	...respond by text message to a question about your daily habits?	○	○	○	○	○	○	○
TchRdy06c	...visit a website weekly to look through lessons, information, and resources, related to your health and wellness goals?	○	○	○	○	○	○	○
TchRdy20c	...attend a group webinar *(online discussion and training)* every 3 weeks to learn more about health and wellness?	○	○	○	○	○	○	○
TchRdy21c	...encourage coworkers to try the things you have learned in a health and wellness program?	○	○	○	○	○	○	○
TchRdy22c	...read a short chapter in a book each week with information related to a health and wellness program you are attending?	○	○	○	○	○	○	○
TchRdy23c	...track your daily health habits? *(like stress management, sleep, meditation, or physical activity)*	○	○	○	○	○	○	○
TchRdy24c	...use a journal each day to reflect on your personal growth and wellness journey?	○	○	○	○	○	○	○
TchRdy03c	...weigh yourself each day?	○	○	○	○	○	○	○
TchRdy25c	...decrease prolonged sitting each day? *(sitting more than 30 minutes without moving around)*	○	○	○	○	○	○	○
TchRdy26c	...sleep 7 to 9 hours each night?	○	○	○	○	○	○	○
TchRdy18c	...add more exercise or physical activity to your weekly routine?	○	○	○	○	○	○	○
TchRdy14c	...do a vigorous activity (e.g. running game) with the children in your classroom everyday?	○	○	○	○	○	○	○
TchRdy1c	...exercise 150 or more minutes per week? *(about 22 minutes per day)*	○	○	○	○	○	○	○
TchRdy15c	...start an active game with children in your classroom everyday?	○	○	○	○	○	○	○
TchRdy2c	...wear a fitness/activity tracker to help monitor your exercise? *(like a Fitbit, Amazfit band, or smart watch)*	○	○	○	○	○	○	○
TchRdy27c	...lead classroom activities that encourage gratitude, forgiveness, and acceptance each day?	○	○	○	○	○	○	○
TchRdy28c	...practice personal mindfulness through meditation, gratitude practice, and self-compassion for at least 5 minutes each day?	○	○	○	○	○	○	○
TchRdy29c	...do a mindfulness or calming activity with the children in your classroom most days? *(e.g. eye closed breathing, thankful thinking)*	○	○	○	○	○	○	○
TchRdy30c	...add new mindfulness and calming techniques to your weekly routine?	○	○	○	○	○	○	○
TchRdy31c	...spend 2 minutes before getting out of bed each morning to think of 5 people you are grateful for and why?	○	○	○	○	○	○	○

Stress PSS

TchStrs12

How would you rate your ability to handle stress?

- ○ Terrible (1)
- ○ Poor (2)
- ○ Fair (3)
- ○ Good (4)
- ○ Excellent (5)

TchStrs_intro1

Below is a list of some of the ways you may have felt or behaved. For each, please indicate how often you felt or thought a certain way in the past month. *In the last month, how often have you...*

	In the last month, how often have you...	*Never*	*Almost Never*	*Some times*	*Fairly Often*	*Very Often*
TchStrs1	...been upset because of something that happened unexpectedly?	○	○	○	○	○
TchStrs2	...felt that you were unable to control the important things in your life?	○	○	○	○	○
TchStrs9	...been angered because of things that were outside of your control	○	○	○	○	○
TchStrs3	...felt nervous and stressed?	○	○	○	○	○
TchStrs4	...felt confident about your ability to handle your personal problems?	○	○	○	○	○
TchStrs5	...felt that things were going your way?	○	○	○	○	○
TchStrs6	...found that you could not cope with all the things that you had to do?	○	○	○	○	○
TchStrs7	...been able to control irritations in your life?	○	○	○	○	○
TchStrs8	...felt that you were on top of things?	○	○	○	○	○
TchStrs10	...felt difficulties were piling up so high that you could not overcome them?	○	○	○	○	○
TchStrs11	...found it hard to deal with the stress in your life?	○	○	○	○	○

TchStrs13

During the past week, how often have you experienced stress that makes you feel tense, restless, nervous, anxious, or has caused you to lose sleep because your mind was troubled all the time?

- ○ Never (1) (1)
- ○ Almost Never (2) (2)
- ○ Rarely (2) (3)
- ○ Sometimes (4) (4)
- ○ Often (5) (5)
- ○ Very Often (6) (6)

MOS 36

SF36_Intro
The next questions ask for your views about your health. Thank you for participating.

SF36_02
Compared to one year ago, how would you rate your health in general now?

- Much better now than one year ago (1)
- Somewhat better now than one year ago (2)
- About the same (3)
- Somewhat worse now than one year ago (4)
- Much worse now than one year ago (5)

SF36_03grp
The following items are about activities you might do during a typical day. **Does your health now limit you in these activities? If so, how much?**

	Yes, limited a lot (1)	Yes, limited a little (2)	No, not limited at all (3)
**Vigorous activities**, such as running, lifting heavy objects, participating in strenuous sports (SF36_03)			
**Moderate activities**, such as moving a table, pushing a vacuum cleaner, bowling, or playing golf (SF36_04)			
Lifting or carrying groceries (SF36_05)			
Climbing **several** flights of stairs (SF36_06)			
Climbing **one** flight of stairs (SF36_07)			
Bending, keeling, or stooping (SF36_08)			
Walking **more than a mile** (SF36_09)			
Walking **several blocks** (SF36_10)			
Walking **one block** (SF36_11)			
Bathing or dressing yourself (SF36_12)			

SF36_13grp
During the past 4 weeks, have you had any of the following problems with your work or other regular daily activities as a result of your physical health?

	Yes (1)	No (0)
Cut down the **amount of time** you spent on work or other activities (SF36_13)		
**Accomplished less** than you would like (SF36_14)		
Were limited in the **kind** of work or other activities (SF36_15)		
Had **difficulty** performing the work or other activities (for example, it took extra effort) (SF36_16)		

SF36_17grp
During the past 4 weeks, have you had any of the following problems with your work or other regular daily activities as a result of any emotional problems (such as feeling depressed or anxious)?

	Yes (1)	No (0)
Cut down the **amount of time** you spent on work or other activities (SF36_17)		
**Accomplished less** than you would like (SF36_18)		
Didn't do work or other activities **as carefully as usual** (SF36_19)		

SF36_20
During the past 4 weeks, to what extent has your physical health or emotional problems interfered with your normal social activities with family, friends, neighbors, or groups?
- ○ Not at all (1)
- ○ Slightly (2)
- ○ Moderately (3)
- ○ Quite a bit (4)
- ○ Extremely (5)

SF36_21
How much bodily pain have you had during the past 4 weeks?
- ○ None (1)
- ○ Very mild (2)
- ○ Mild (3)
- ○ Moderate (4)
- ○ Severe (5)
- ○ Very Severe (6)

SF36_22
During the past 4 weeks, how much did pain interfere with your normal work? *including both work outside the home and housework*
- ○ Not at all (1)
- ○ A little bit (2)
- ○ Moderately (3)
- ○ Quite a bit (4)
- ○ Extremely (5)

SF36_23grp
These questions are about how you feel and how things have been with you during the past 4 weeks. For each question, please give the one answer that comes closest to the way you have been feeling.

How much of the time during the past 4 weeks...

	All of the time (1)	Most of the time (2)	A good bit of time (3)	Some of the time (4)	A little of the time (5)	None of the time (6)
Did you feel full of pep? (SF36_23)						
Have you been a very nervous person? (SF36_24)						
Have you felt so down in the dumps that nothing could cheer you up? (SF36_25)						
Have you felt calm and peaceful? (SF36_26)						
Did you have a lot of energy? (SF36_27)						
Have you felt downhearted and blue? (SF36_28)						
Did you feel worn out? (SF36_29)						
Have you been a happy person? (SF36_30)						
Did you feel tired? (SF36_31)						

SF36_32
During the past 4 weeks, how much of the time has your physical health or emotional problems interfered with your social activities (like visiting with friends, relatives, etc.)**?**

- All of the time (1)
- Most of the time (2)
- Some of the time (3)
- A little of the time (4)
- None of the time (5)

SF36_33grp
How TRUE or FALSE is each of the following statements for you.

	Definitely true (1)	Mostly true (2)	Don't know (3)	Mostly false (4)	Definitely false (5)
I seem to get sick a little easier than other people (SF36_33)					
I am as healthy as anybody I know (SF36_34)					
I expect my health to get worse (SF36_35)					
My health is excellent (SF36_36)					

Social DOH

SoDHintro Below are questions about your life and health. *We understand that these questions may be personal, but they are important and will be kept in strict confidence. You can skip a question if you prefer.*

SODH01

Do you have a primary care provider for your medical care? *Doctor's office where you are a registered patient Provider you go to for routine check-ups or when sick*

- ○ YES (1)
- ○ NO (0)

SODH02

There are many reasons people delay getting medical care. **Have you delayed getting medical care in the PAST 12 MONTHS because of cost or lack of insurance coverage?**

- ○ YES (1)
- ○ NO (0)

SODH3grp

How true are each of these statements for you?

	Never True (1)	Sometimes True (2)	Often true (3)
People in my neighborhood help each other out (SODH03)			
Within the past 12 months, you worried that your food would run out before you got money to buy more. (SODH04)			
There are people in my neighborhood I can count on. (SODH05)			
Within the past 12 months, the food you bought just didn't last and you didn't have money to get more. (SODH06)			
The place I live feels safe. (SODH07)			
I feel physically and emotionally cared for at home. (SODH08)			

SODH09grp

In the past two weeks how often have you felt the following ways?

	Not at all (1)	Several days (2)	More than half the days (3)	Nearly every day (4)
Little interest or pleasure doing things (SODH09)				
Feeling down depressed or hopeless (SODH10)				

SODH11

In the next 2 months are you worried you may not have stable housing?

- ○ YES (1)
- ○ NO (0)

SODH12

Think about the place you live. **Do you have problems with the following?** *(mark all that apply)*

- ▢ Mold (1)
- ▢ Water leaks (2)
- ▢ Lead paint or pipes (3)
- ▢ Inadequate heat or air conditioning (4)
- ▢ No or not working smoke detectors (5)

- ☐ Oven or stove not working (6)
- ☐ Pest infestation such as mice, ants, or bugs (7)
- ☐ ⊗None of the above (8)

SODH13

How hard is it for you to pay for the very basics like food, housing, medical care, and heating?

- ○ Very Hard (1)
- ○ Somewhat Hard (2)
- ○ Not Hard at all (3)

SODH14

In the past 12 months, has lack of transportation kept you from medical appointments, meetings, work or from getting things needed for daily living?

- ○ YES (1)
- ○ NO (0)

SODH15

In the past 12 months has the electric, gas, oil, or water company threatened to shut off services in your home?

- ○ YES (1)
- ○ NO (0)

Worksite Wellness *(Center – level outcomes, staff get random selection of items)*

CNTwell_INTRO

The next set of questions are about your center's current efforts to promote health, wellness, and safety among staff.
As you answer questions, please focus on what your center has been doing during the past 6 months.

CNTwell_offerYN

During the past 6 months, did your center offer or promote any staff wellness, health, or safety programs, educational materials, or trainings?

- This includes programs, educational materials, and trainings offered by your center, a local community organization, insurance company, or other group.
- *Programs include things like group meetings, classes, or activities.*
  - *Educational materials can be print/paper or electronic and offer helpful information about a health or safety-related topic or issue.*
  - *Trainings include activities that might be required to work, issue continuing education credits, or result in a certification/certificate*

○ YES (1)
○ NO (0)

Skip To: CNTwell_BenfA If During the past 6 months, did your center offer or promote any staff wellness, health, or safety... = NO

CNTwell_Topics

During the past 6-months, **Were any staff wellness, health, or safety programs, materials, or trainings offered that covered these topics?** *[Mark all topics covered including those you did NOT participated in]*

▢ Physical activity and exercise (1)
▢ Personal nutrition (2)
▢ Weight management (3)
▢ Stress management (4)
▢ Sleep health (5)
▢ Meditation/Relaxation (6)
▢ Smoking cessation (7)
▢ Breathing/calming techniques (8)
▢ Resilience (9)
▢ Flu, cold, illness prevention (10)
▢ Injury prevention at work (11)
▢ Reducing exposure to work place hazards (12)
▢ Reducing work stress (13)
▢ Staff communication or conflict resolution (14)
▢ Back health (15)
▢ Strength Training (16)

Carry Forward Selected Choices from "During the past 6-months, Were any staff wellness, health, or safety programs, materials, or trainings offered that covered these topics? [Mark all topics covered including those you did NOT participated in]"

CNTwell_BenfA

During the past 6 months, did your center offer any of the following for center staff? Our center was able to provide...

	**YES** (1)	**NO** (0)
...a mentor for new teachers (CNTbenefit01)		
...behavior specialist for classroom help (CNTbenefit02)		
...reduced/free tuition for staff member's child (CNTbenefit03)		

...on-site gym or space for working out (CNTbenefit04)		
...free or low cost membership to local gym/fitness center (CNTbenefit05)		
...free or low cost mental health support (CNTbenefit06)		
...space at center for quiet relaxation that staff can use (CNTbenefit07)		
...health risk appraisals (CNTbenefit08)		
...health assessments or screenings (CNTbenefit09)		
...free meal(s) and snack when working (CNTbenefit10)		
...common space separate from children for socializing or group activities. (CNTbenefit11)		
...training for new teachers and staff (CNTbenefit12)		

Carry Forward Selected Choices from "During the past 6 months, did your center offer any of the following for center staff? Our center was able to provide..."

CntWellBenUse
Of these things that your center provided in past 6 months, which did you use or take advantage of? [Select all that you used]

- ▢ ...a mentor for new teachers (1)
- ▢ ...behavior specialist for classroom help (2)
- ▢ ...reduced/free tuition for staff member's child (3)
- ▢ ...on-site gym or space for working out (4)
- ▢ ...free or low cost membership to local gym/fitness center (5)
- ▢ ...free or low cost mental health support (6)
- ▢ ...space at center for quiet relaxation that staff can use (7)
- ▢ ...health risk appraisals (8)
- ▢ ...health assessments or screenings (9)
- ▢ ...free meal(s) and snack when working (10)
- ▢ ...common space separate from children for socializing or group activities. (11)
- ▢ ...training for new teachers and staff (12)

CNTwell_stfint
In your opinion, how interested do you think staff are in participating in health and wellness programs offered through the center?
Examples: Diabetes prevention, Back health, Weight loss, Healthy eating, Physical activity, Stress reduction Try to rate their overall interest on a scale of 1 to 10, where 1 is not at all interested and 10 is extremely interested.

- ○ 1 Not at all interested (1)
- ○ 2 (2)
- ○ 3 (3)
- ○ 4 (4)
- ○ 5 (5)
- ○ 6 (6)
- ○ 7 (7)
- ○ 8 (8)
- ○ 9 (9)
- ○ 10 Extremely Interested (10)

CNTwell_CommHealthYN
During the past 6 months, **Did your center and staff work with other businesses or community organizations on improving the health and wellness of the city, town, county, or community where your center is located?**

- ○ YES (1)

- NO (0)

CNTwell_offerimp

How important is it for your center to offer health and wellness programs for staff? *Examples: Diabetes prevention, Back health, Weight loss, Healthy eating, Physical activity, Stress reduction*

- 1 Not at all important (1)
- 2 (2)
- 3 (3)
- 4 (4)
- 5 (5)
- 6 (6)
- 7 (7)
- 8 (8)
- 9 (9)
- 10 Extremely Important (10)

CNTwell_goals

Does your center have goals, written policies, or action plans for staff wellness programs and activities?

- YES (1)
- NO (0)
- Not Sure (2)

CNTwell_ComOrg

During the past 6 months, **Did your center offer or promote any health, wellness, or safety related resources from any of these community organizations?** *Mark all that apply*

- ▢ State or County Health Department (1)
- ▢ Health Insurance Provider (2)
- ▢ Insurance Company (3)
- ▢ Health Related Organization (American Heart Assoc, American Cancer Society) (4)
- ▢ Local Hospital (5)
- ▢ YMCA or YWCA (6)
- ▢ Town, city, or county government organization (Chamber of commerce, town wellness council) (7)
- ▢ Parks and Recreation Department (local or state) (8)
- ▢ Local Gym or Fitness center (9)
- ▢ Community College or Local University (10)
- ▢ Other Community Group (11)
- ▢ ⊗We have NOT worked with any of these organizations (12)

CNTwell_who

During the past 6-months, **Who is mainly responsible for planning, finding, and getting word to staff about wellness and health initiatives/programs?**

- Individual: Administrative staff (Director, Assistant Director, HR, Office Manager) (1)
- Individual: Teacher or other staff (2)
- Small group of administrative staff (3)
- Small group of teachers/staff (4)
- Group, or committee, including teachers and administrative staff (5)
- No one at our center does this (6)
- I am not sure (7)

CNTwell_inform

What are the top ways, staff are informed about staff wellness, health, and safety programs and activities being offered?

Select 1 to 4 methods most often used

- ☐ Employee Orientation (1)
- ☐ During Staff Meetings (2)
- ☐ Texts (3)
- ☐ Emails (4)
- ☐ Flyers or Brochures (5)
- ☐ Bulletin Board or Resource Table in Staff Area (6)
- ☐ Newsletter (7)
- ☐ Personal Conversations (8)
- ☐ Word of Mouth (9)
- ☐ Insurance Company (10)
- ☐ Other (specify) (11) ____

CNTwell_BenfB

During the past 6 months, did your center offer any of the following for center staff? Our center was able to provide...

	**YES** (1)	**NO** (0)
...health insurance (CNTbenefit13)		
...funding assistance for educational costs (CNTbenefit14)		
...retirement program such as 401K (CNTbenefit15)		
...employer contributions to retirement savings (CNTbenefit16)		
...paid maternity leave (CNTbenefit17)		
...paid sick leave (CNTbenefit18)		
...paid vacation days (CNTbenefit19)		
...paid time-off for job-related training or classes (CNTbenefit20)		
...ability to take unpaid leave (CNTbenefit21)		
...help with transportation to and from work (CNTbenefit22)		

CntWell_Champ

Do you feel like the center where you work has an employee wellness champion, or network of champions, who actively publicize and promote health and wellness programs? *Wellness Champions are health-minded employees who are passionate about creating a healthier workplace. They serve as positive role models, provide peer support, and offer feedback to leadership to improve wellness offerings.*

- ○ YES (1)
- ○ NO (0)

The questions in this section ask how you feel about different aspects of your job at this child care center. *If you have more than one job, please answer questions as they apply to your job at the center involved in the OnWARD program.*

WellBq01
Which response completes this statement best for you today. **Overall, I am ______ with my job.**
- not at all satisfied (1)
- not too satified (2)
- somewhat satisfied (3)
- very satisfied (4)

WellBq02
Which response completes this statement best for you today. **I am ______ with my wages.**
- not at all satisfied (1)
- not too satified (2)
- somewhat satisfied (3)
- very satisfied (4)

WellBq03
Which response completes this statement best for you today. **I am ______ with my chances for advancement in this job.**
- not at all satisfied (1)
- not too satified (2)
- somewhat satisfied (3)
- very satisfied (4)

WellBq04
Which response completes this statement best for you today. **I am ______ with the benefits provided by my employer.**
- not at all satisfied (1)
- not too satified (2)
- somewhat satisfied (3)
- very satisfied (4)

WellBq13a
How often do you experience physical fatigue when you are working?
- Never (1)
- Almost Never *(a few times a year or less)* (2)
- Rarely *(once a month or less)* (3)
- Sometimes *(a few times a month)* (4)
- Often *(once a week)* (5)
- Very Often *(a few times a week)* (6)
- Always *(every day)* (7)

WellBq13b
How often do you experience mental fatigue when you are working?
- Never (1)
- Almost Never *(a few times a year or less)* (2)
- Rarely *(once a month or less)* (3)
- Sometimes *(a few times a month)* (4)
- Often *(once a week)* (5)
- Very Often *(a few times a week)* (6)
- Always *(every day)* (7)

WellBq14

How often true for you? **My work inspires me.**

- ○ Never (1)
- ○ Almost Never *(a few times a year or less)* (2)
- ○ Rarely *(once a month or less)* (3)
- ○ Sometimes *(a few times a month)* (4)
- ○ Often *(once a week)* (5)
- ○ Very Often *(a few times a week)* (6)
- ○ Always *(every day)* (7)

WellBq15
How often true for you? **I am immersed in my work.**

- ○ Never (1)
- ○ Almost Never *(a few times a year or less)* (2)
- ○ Rarely *(once a month or less)* (3)
- ○ Sometimes *(a few times a month)* (4)
- ○ Often *(once a week)* (5)
- ○ Very Often *(a few times a week)* (6)
- ○ Always *(every day)* (7)

WellBq16
How often true for you? **When I get up in the morning, I feel like going to work.**

- ○ Never (1)
- ○ Almost Never *(a few times a year or less)* (2)
- ○ Rarely *(once a month or less)* (3)
- ○ Sometimes *(a few times a month)* (4)
- ○ Often *(once a week)* (5)
- ○ Very Often *(a few times a week)* (6)
- ○ Always *(every day)* (7)

WellBq27
How often do the demands of your job interfere with your personal life?

- ○ Never (1)
- ○ Almost Never *(a few times a year or less)* (2)
- ○ Rarely *(once a month or less)* (3)
- ○ Sometimes *(a few times a month)* (4)
- ○ Often *(once a week)* (5)
- ○ Very Often *(a few times a week)* (6)
- ○ Always *(every day)* (7)

WellBq28
How often do the demands of your personal life interfere with your work at this center?

- ○ Never (1)
- ○ Almost Never *(a few times a year or less)* (2)
- ○ Rarely *(once a month or less)* (3)
- ○ Sometimes *(a few times a month)* (4)
- ○ Often *(once a week)* (5)
- ○ Very Often *(a few times a week)* (6)
- ○ Always *(every day)* (7)

The questions in this section ask how you feel about different aspects of your job at this child care center. *If you have more than one job, please answer questions as they apply to your job at the center involved in the OnWARD program.*

How much do you agree or disagree with each statement?

	How much do you agree or disagree with each statement about your job at this center.	*Strongly Disagree (1)*	*Disagree (2)*	*Slightly Disagree (3)*	*Slightly Agree (4)*	*Agree (5)*	*Strongly Agree (6)*
WellBq05	I can count on my supervisor for support when I need it.						
WellBq06	I can count on my coworkers for support when I need it.						
WellBq07	I feel my job is secure.						
WellBq08	I am given a lot of freedom to decide how to do my own work.						
WellBq09	I never seem to have enough time to get everything done on my job.						
WellBq10	The work I do is meaningful to me.						
WellBq11	The work I do serves a greater purpose.						
WellBQ17	At my center, I am treated with respect.						
WellBQ18	My center values my contributions.						
WellBq19	My organization cares about my general satisfaction at work.						
WellBQ20	My organization is willing to extend resources in order to help me perform my job to the best of my ability.						
WellBQ21	I receive recognition for a job well done.						
WellBQ22	I trust the management at my organization.						
WellBQ23	The center where I work is committed to employee health and well-being.						
WellBq24	The center where I work encourages me and provides opportunities to engage in healthy behaviors, such as being physically active, eating a healthy diet, living tobacco free, and managing my stress.						
WellBqe06	I feel motivated in my work.						
WellBqe07	I take pride in my work						
WellBqE11	I think of the people I work with as friends.						
WellBqE12	The people I work with support my health and wellness efforts.						
WellBqE08	My coworkers and I are happy to work alongside each other.						
WellBqE09	My coworkers and I understand and respect each other.						
WellBqE10	My coworkers and I communicate job information to each other effectively.						
TchOutExp5	Having good exercise habits will improve my job performance.						
TchOutExp10	Having good mental and emotional health habits will improve my job performance.						

WBQ_Praise7day

In the last seven days, I have received recognition or praise for doing good work from the center director or assistant director.

- YES (1)
- NO (2)

Absenteeism/Turnover

The next few questions ask about your feeling about the center where your work.

TchWrk_turn02

Over the last month, **How often did you think about getting another job that will better suit your personal needs?**

- Never (1) (1)
- Almost Never (2) (2)
- Rarely (2) (3)
- Sometimes (4) (4)
- Often (5) (5)
- Very Often (6) (6)

TchWrk_turn01

Over the last month, **How often have you seriously considered leaving your current job at this center?**

- Never (1) (1)
- Almost Never (2) (2)
- Rarely (2) (3)
- Sometimes (4) (4)
- Often (5) (5)
- Very Often (6) (6)

TchWrk_turn03

If offered to you, **How likely are you to accept another job** (not at this center) **at the same pay level?**

- Highly Unlikely (1)
- Unlikely (2)
- Slightly Unlikely (3)
- Slightly Likely (4)
- Likely (5)
- Highly Likely (6)

TchWrk_PresGrp

The next questions are about the time you spent during your hours working at this center in the past 4 weeks. Select the response for each that comes closest to your experience.

In the past month, how often did you...

	Never (1)	*Almost Never (2)*	*Rarely (3)*	*Some times (4)*	*Often (5)*	*Very Often (6)*
...not concentrate enough on your work? (TchWrkPref03)						
...find yourself not working as carefully as you should? (TchWrkPref04)						
...find decision making difficult? (TchWrkPref05)						
...notice the quality of your work lower than it should have been? (TchWrkPref06)						
...find it difficult to "get going" at the begining of your workday? (TchWrkPref07)						
...notice your job performance was higher than other center staff? (TchWrkPref08)						
...feel you got less done than other center staff? (TchWrkPref09)						

The next set of questions ask about staffing at your center and some work related behaviors.

CntHire_Full
Does your center currently have enough staff to operate at full capacity?
- ○ Yes (1)
- ○ No (0)
- ○ Not Sure (2)

CntStaffing
How much do you agree or disagree with these statements about staffing at your center.

	Strongly Disagree (1)	*Disagree (2)*	*Slightly Disagree (3)*	*Slightly Agree (4)*	*Agree (5)*	*Strongly Agree (6)*
**Keeping or retaining quality staff at our center is an issue.** (CntStaffing01)						
**Finding quality staff is hard.** (CntStaffing02)						
**Staff turnover makes improving our center difficult.** (CntStaffing03)						
**Staff missing work is an issue at our center.** (CntStaffing04)						

TchWrk_MissYN
Most people miss, or can not go to, work sometimes. We get sick, have appointments, or need to take care of friends or family. **In the past 4 weeks (about 28 days) have you missed any scheduled work time at this center?** *include missing full shift, part of a shift, or being late by more than 15 minutes.* *could be planned (eg. appointment) or unexpected absence (eg. child got sick)*
- ○ YES (1)
- ○ NO (0)

Skip To: TchWrk_MissReason2 If Most people miss, or can not go to, work sometimes. We get sick, have appointments, or need to ta... = NO

TchWrk_missdays
With this question we are asking about the times you missed, or could not go to, work for planned and unplanned reasons.
planned - You were able to ask for time-off ahead of time, center administration was able prepare for your absence
unplanned - Your absence was unexpected and you had to let center administration know you would miss work on short notice. We ask about three types of missed work time: missing full shift, partial shift, and being late.

In the past 4 weeks (about 28 days), how many days did you...

	**Planned / Let center know ahead of time** *(# days you missed work)*	**Unplanned / NOT expected** *(# days you missed work)*
**...missed an entire shift, or workday.** (CntHire_MissFull)	*Drop down 0 to 28 days*	*Drop down 0 to 28 days*
**...missed part of a shift, or part of workday** *(arrive late or leave early by more than 60 minutes)* (CntHire_MissPart)	*Drop down 0 to 28 days*	*Drop down 0 to 28 days*
**...miss 15 to 60 minute because they were late to work.** (CntHire_MissLate)	*Drop down 0 to 28 days*	*Drop down 0 to 28 days*

TchWrk_MissReason
Over the days you missed an entire or partial shift for planned or unplanned reasons, **What were the 1 or 2 primary reasons for missing work on those days?**
- ▢ I was sick or injured (1)
- ▢ My child was sick or injured (2)
- ▢ Caring for family or friend other than your child (3)
- ▢ Transportation issues (4)
- ▢ Did not feel like working (5)
- ▢ Forgot I was scheduled (6)
- ▢ Appointments or errands (non-medical) (7)

- ☐ Medical Appointments (8)
- ☐ Other (9) ____

Display this question:
If Most people miss, or can not go to, work sometimes. We get sick, have appointments, or need to ta... = NO

TchWrk_MissReason2
Over the last 6 months, **What were the 1 or 2 primary reasons you missed work at your center?**

- ☐ ⊗I have not missed work in past 6 months (10)
- ☐ I was sick or injured (1)
- ☐ My child was sick or injured (2)
- ☐ Caring for family or friend other than your child (3)
- ☐ Transportation issues (4)
- ☐ Did not feel like working (5)
- ☐ Forgot I was scheduled (6)
- ☐ Appointments or errands (non-medical) (7)
- ☐ Medical Appointments (8)
- ☐ Other (9) ____

TchWork_AskExtraShif
In the past 4 weeks (about 28 days), **How many days did center staff ask you to work extra on short notice because another staff member did not come to work or left work early?** *This would include asking you to come in early, leave later than scheduled, or work on a scheduled day-off.*

- ○ DROPDOWN: 0 days (0) to 28 Days

TchWork_ShiftCancel
In the past 4 weeks (about 28 days), **How many days did your center cancel a shift you were scheduled to work on short notice?**

- ○ DROPDOWN: 0 days (0) to 28 Days

TchWrk_SchChg
In the past 4 weeks, **How many times did your work schedule for this center change on short notice?**

- ○ 0 times (1)
- ○ 1 time (2)
- ○ 2 times (3)
- ○ 3 times (4)
- ○ 4 times (5)
- ○ 5 times (6)
- ○ 6 or more times (8)

TchWrkPerf10
How would you compare your overall job performance on the days you worked during the past 4 weeks with the performance of most other workers who have a similar type of job at this center?

- ○ You were a lot better than other workers (1)
- ○ You were somewhat better than other workers (2)
- ○ You were a little better than other workers (3)
- ○ You were about average (4)
- ○ You were a little worse than other workers (5)
- ○ You were somewhat worse than other workers (6)
- ○ You were a lot worse than other workers (7)

Director

[All surveys administered through online system (Qualtrics)]

Demographics

The next few questions ask a little about you.

DirDem_position

What is your position at the center?

- ○ Director (1)
- ○ Assistant Director (2)

DirDem_Owner

Are you the owner of this center?

- ○ YES (1)
- ○ NO (0)

DirDem_PosYear

About how long have you been the ${DirDem_position/ChoiceGroup/SelectedChoices} for this child care program?

- DROPDOWN: Less than 3 months (2) to 20 years or more (240)

DirDem_CntWrkYrs

About how long have you worked at this child care program?

(working in any position at the center)

- DROPDOWN: Less than 3 months (2) to 20 years or more (240)

DirDem_wrkFull

Do you currently work part-time or full-time at this child care program?

- ○ Part-Time (less than 30 hours per week) (1)
- ○ Full-Time (30 or more hours per week) (2)

DirDem_wrkHrs

About how many hours do you work for this center during an average week?

- ○ Less than 5 hours per week (3)
- ○ 5 (5)
- ○ 10 (10)
- ○ 15 (15)
- ○ 20 hours per week (20)
- ○ 25 (25)
- ○ 30 (30)
- ○ 35 (35)
- ○ 40 (40)
- ○ 45 hours per week (45)
- ○ 50 (50)
- ○ 55 (55)
- ○ 60 (60)
- ○ 65 or more hours per week (65)

DirDem_edu

What is the highest level of school you have completed?

- ○ Middle School (less than 9th grade) (1)
- ○ Some High School (2)
- ○ High school diploma or equivalent (GED) (3)

- Some college (4)
- Associate degree (5)
- College graduate (6)
- Masters/Doctoral degree (7)
- Prefer not to answer (77)

DirDem_CEtrain
Have you completed any training related to early childhood education and care through a community college, university, or other accredited training program?
- YES (1)
- NO (0)

Display this question:
If Have you completed any training related to early childhood education and care through a community... = YES
And If
What is the highest level of school you have completed? = Associate degree
Or What is the highest level of school you have completed? = College graduate
Or What is the highest level of school you have completed? = Masters/Doctoral degree
Or What is the highest level of school you have completed? = Prefer not to answer

DirDem_CEdegree
Do you have a degree, or certificate, in child development or teaching?
- Yes (1)
- No (0)

DirDem_Age
What is your age? (in years, number only)________________________________

DirDem_sex1
Which of the following describes you best?
- Male (1)
- Female (2)
- Provide other options --> (3)

Display this question:
If Which of the following describes you best? = Provide other options -->

DirDem_sex2
Which of the following describes you?
[mark all that apply]
- ☐ Female (1)
- ☐ Male (2)
- ☐ Non-binary (4)
- ☐ Transgender (5)
- ☐ Cisgender (6)
- ☐ Agender (7)
- ☐ Gender queer (8)
- ☐ Self-Describe (specify) (9) ____
- ☐ ⊗Prefer no response (77)

DirDem_LivSit
Which of the following best describes your current living situation?
- Living alone (i.e. no other adult) (1)
- Living with parent(s) or roommate(s) (2)
- Living with a partner/spouse (3)
- Living temporarily with a relative/friend (short term) (4)

- ○ Self Describe (5) ____

DirDem_Nadlt

Including yourself, how many adults live in your household? *(Adult is anyone 18+ years who helps with household expenses or duties)*

- ○ 1 (just me) (1)
- ○ 2 adults (2)
- ○ 3 adults (3)
- ○ 4 or more adults (4)

DirDem_Nchild

How many children (under 18 years) live in your household?

- ○ 0 (no children) (0)
- ○ 1 child (1)
- ○ 2 children (2)
- ○ 3 children (3)
- ○ 4 or more children (4)

DirDem_Eng

Are you fluent in a language other than English? *(e.g. Spanish, ASL, Chinese, German)*

- ○ YES (1)
- ○ NO (0)

Display this question:

If Are you fluent in a language other than English? (e.g. Spanish, ASL, Chinese, German) = YES

DirDem_Lang

Which language(s), other than English, do you speak fluently? *[Mark all that apply]*

- ☐ Spanish (1)
- ☐ American Sign language (hearing impaired) (2)
- ☐ Chinese (Mandarin, Cantonese, Hokkien and other varieties) (3)
- ☐ Tagalog (including Filipino) (4)
- ☐ Vietnamese (5)
- ☐ French (6)
- ☐ Arabic (7)
- ☐ Korean (8)
- ☐ Other (specify) (9) ____

DirDem_race

Which of these race/ethnicity options describe you? *[Mark all that apply]*

- ☐ American Indian or Alaska Native (1)
- ☐ Asian (including India) (2)
- ☐ Black or African-American (3)
- ☐ Hispanic, Latino/a/x, or Spanish origin (4)
- ☐ Native Hawaiian or Other Pacific Islander (5)
- ☐ White (6)
- ☐ Self-Describe (specify) (7) ____
- ☐ ⊗Prefer not to answer (77)

Display this question:

If Which of these race/ethnicity options describe you? [Mark all that apply] != Hispanic, Latino/a/x, or Spanish origin

DirDem_Ethn **Do you consider yourself of Hispanic, Latino/a/x, or Spanish origin?**

- ○ Yes, Hispanic, Latino/a/x, or Spanish ethnicity (1)

- ○ No, Non-Hispanic (2)
- ○ Prefer not to answer (3)

Display this question:
If Which of these race/ethnicity options describe you? [Mark all that apply] = Hispanic, Latino/a/x, or Spanish origin
Or Do you consider yourself of Hispanic, Latino/a/x, or Spanish origin? = Yes, Hispanic, Latino/a/x, or Spanish ethnicity

DirDem_Hisp **Which of these options describes you?** *[Mark all that apply]*

- ☐ Cuban (1)
- ☐ Mexican, Mexican-American, Chicano/a/x (2)
- ☐ Central-American (3)
- ☐ Spanish (4)
- ☐ Puerto Rican (5)
- ☐ Self-Describe (specify) (6) ____
- ☐ ⊗Prefer not to answer (77)

DirDem_income **Please provide an estimate of the total annual income for your household?** includes income from all adults contributing to household expenses

- ○ under $25,000 per year (25000)
- ○ $25,000 to $34,999 per year (30000)
- ○ $35,000 to $44,999 per year (40000)
- ○ $45,000 to $54,999 per year (50000)
- ○ $55,000 to $64,999 per year (60000)
- ○ $65,000 to $74,999 per year (70000)
- ○ $75,000 to $84,999 per year (80000)
- ○ $85,000 to $99,999 per year (90000)
- ○ $100,000 or more per year (100000)
- ○ Prefer not to answer (77)

DirDem_preg
Are you currently pregnant?

- Why we ask...People who are pregnant can participate.
    - ○ We do not disclose who might be pregnant, but we are required by funding agency to report the number, or percent, of people in a few broad categories in progress reports a few times each year (e.g. # of women, # by age group [20-30yrs], % who might be prenant).
    - ○ Example. Of the 200 people currently enrolled in the program 3% report being pregnant.
- ○ YES (1)
- ○ NO (0)

TchWtHis_Height
How tall are you without shoes? (current height)

- DROPDOWN: under 4 ft (1) to 7ft or taller

TchWtHis_Weight
About how much do you currently weigh without shoes (in pounds)? (in pounds, number only)____________________

DirHealthInsur
What type of health insurance do you currently have?

- ○ I currently DO NOT have health insurance (1)
- ○ Health insurance through my employer (this childcare center) (2)
- ○ Health insurance through spouse's employer (3)
- ○ Medicare (4)
- ○ Medicaid (5)

- Health insurance through HealthCare.gov or other insurance marketplace (6)
- Other (specify) (7) ____

DirDentalInsur

Do you currently have insurance that covers dental care?

- Yes (1)
- No (0)

These questions ask about programs, behaviors, or therapies you may be currently involved with. We are asking about things you are currently doing.

**Are you currently...**	**NO** (0)	**YES** (1)
...enrolled in a stress management or meditation program? (TchBehBL01_1)		
...enrolled in an exercise program or regular exercise class? *(gym, fitness center, recreation dept, community center)* (TchBehBL01_2)		
... working with a nutritionist or dietician to improve your diet? (TchBehBL01_3)		
...working with an exercise trainer or physical therapist to increase your physical activity level? (TchBehBL01_4)		
...going to individual, or group, therapy or working with a mental health provider or employee assistance program, for counseling? (TchBehBL01_5)		

Display this question:

If These questions ask about programs, behaviors, or therapies you may be currently involved with. W... = ...enrolled in a stress management or meditation program? [YES]

TchBehBLstress

About how long have you been enrolled in meditation or stress management program?

- Less than 1 week (1)
- 1-2 weeks (2)
- 1 month (4)
- 2 months (8)
- 3 months (12)
- 4 months (16)
- 5 months (20)
- 6 months or more (24)

Display this question:

If These questions ask about programs, behaviors, or therapies you may be currently involved with. W... = ...enrolled in an exercise program or regular exercise class? (gym, fitness center, recreation dept, community center) [YES]

TchBehBLexProg

About how long have you been regularly participating in exercises classes or programs?

- Less than 1 week (1)
- 1-2 weeks (2)
- 1 month (4)
- 2 months (8)
- 3 months (12)
- 4 months (16)
- 5 months (20)
- 6 months or more (24)

Display this question:

If These questions ask about programs, behaviors, or therapies you may be currently involved with. W... = ... working with a nutritionist or dietician to improve your diet? [YES]

TchBehBLnutr

About how long have you been following the guidance of the nutritionist or dietician?

- Less than 1 week (1)
- 1-2 weeks (2)
- 1 month (4)
- 2 months (8)
- 3 months (12)
- 4 months (16)
- 5 months (20)
- 6 months or more (24)

Display this question:

If These questions ask about programs, behaviors, or therapies you may be currently involved with. W... = ...working with an exercise trainer or physical therapist to increase your physical activity level? [YES]

TchBehBLexTrain

About how long have you been following the guidance of an exercise trainer or physical therapist?

- Less than 1 week (1)
- 1-2 weeks (2)
- 1 month (4)
- 2 months (8)
- 3 months (12)
- 4 months (16)
- 5 months (20)
- 6 months or more (24)

Display this question:

If These questions ask about programs, behaviors, or therapies you may be currently involved with. W... = ...going to individual, or group, therapy or working with a mental health provider or employee assistance program, for counseling? [YES]

TchBehBLmenhealth

About how long have you been following the guidance of a therapist or mental health counselor?

- Less than 1 week (1)
- 1-2 weeks (2)
- 1 month (4)
- 2 months (8)
- 3 months (12)
- 4 months (16)
- 5 months (20)
- 6 months or more (24)

Display this question:

If Please provide an estimate of the total annual income for your household? includes income from al... = Prefer not to answer

TchDem_incMed56

Is your current total household income more than $55,000 per year?

- YES (1)
- NO (0)

Display this question:

If Please provide an estimate of the total annual income for your household? includes income from al... = Prefer not to answer

TchDem_incMed35

Is your current income more than $35,000 per year?

- YES (1)
- NO (0)

Center Information

The first set of questions asks for a little information about your center.
Thank you for completing this section.

- *This information is only used to describe the group of centers participating in the project, not individual centers. In research reports only summaries for the group as a whole (e.g. averages, % of all people) are presented. For example, "Of the 50 centers enrolled 40% participate in CACFP."*

CntDem_OperateYrs

How many years has your center been in operation?

- ○ Years in operation (number only): (1) ____

CntDem_StarRate

What is your center's current star rating through the North Carolina Star Rating program?

- ○ 1 star (1)
- ○ 2 star (2)
- ○ 3 star (3)
- ○ 4 star (4)
- ○ 5 star (5)
- ○ GS-110 (110)
- ○ No Rating/Do not participate (0)

CntDem_Type

Which of the following would you use to describe your child care program? *[Mark all that apply]*

- ☐ Head Start and/or Early Head Start (1)
- ☐ NC Pre-K (2)
- ☐ School-based Pre-Kindergarten (3)
- ☐ Faith-based organization (4)
- ☐ US Military run program (5)
- ☐ Tribal Nation affiliation (Native American or Alaska Native) (6)
- ☐ National or Regional Chain (5 or more programs/centers with centralized management/ownership) (7)
- ☐ Local Center (1 to 4 centers with local ownership) (8)
- ☐ Other (please describe) (9) ____

Display this question:
If Which of the following would you use to describe your child care program? [Mark all that apply] = National or Regional Chain (5 or more programs/centers with centralized management/ownership)

CntDem_ChainNum

About how many centers are in the chain, or franchise, you are part of?

- ○ Less than 5 (3)
- ○ 5-10 (7)
- ○ 11-20 (16)
- ○ 21-30 (26)
- ○ 31-40 (36)
- ○ 41-50 (46)
- ○ 51 or more (52)

CntDem_NAEYC

Is your child care program accredited by NAEYC *(National Association for the Education of Young Children)***?**

- ○ YES (1)
- ○ NO (0)

CntDem_Subsid
Does your center accept child care subsidies?
- YES (1)
- NO (0)

CntDem_CACFP
Is your center part of the Child and Adult Care Food Program *(i.e. CACFP, the food program)***?**
- YES (1)
- NO (0)

CntDem_sttm
About what time does your center open each day? *(i.e. children can arrive)*

- DROP DOWN 5:00 am to 10:00am

CntDem_edtm
What time does your center close each day? *(i.e most children should be picked up)*

- DROP DOWN 3:00 pm to 8:00 pm

CntDem_tmNote
If the time options above do not represent your center's normal hours of operation, please provide additional information in this box. *(e.g. center is open 24-hours most days, have 2nd or 3rd shift drop off and pick-up times)*________________

CntDem_Meals
Which of the following meals/snacks does your program provide for children most days? *[Mark all that apply]*
- ☐ Breakfast (1)
- ☐ Morning Snack (2)
- ☐ Lunch (3)
- ☐ Afternoon Snack (4)
- ☐ Supper/Dinner (5)
- ☐ Other [specify] (6) ____

CntDem_prog
Are your 2-5 year old classrooms currently using any of these health and wellness programs or curricula? *[Mark all that apply]*
- ☐ Color Me Healthy (1)
- ☐ Healthy Kids, Healthy Future (2)
- ☐ Let's Move Child Care (3)
- ☐ I am Moving I am Learning (4)
- ☐ The Incredible Years (5)
- ☐ Growing Minds (6)
- ☐ Rainbow in My Tummy (7)
- ☐ Go NAPSACC (8)
- ☐ CASEL (social emotional learning) (9)
- ☐ Changing Perspectives (social emotional program) (10)
- ☐ Other Physical Activity, motor skills, or Social Emotional Wellness curriculum *[Specify]* (11) ____________
- ☐ ⊗None of the above (12)

CntDem_Cost
About how much does it cost per month for a 3-4 year old to attend your child care program? (please choose amount closest to cost per month from list below)
- DROPDOWN $0 per month to $1600 or more per month (1600)

The next few questions ask about the children who are enrolled in this child care program.

CntChld_Totnum
About how many children are currently enrolled at this child care program?

____

CntChld_Age
Of the children currently enrolled in your child care program, How many are in each of these age groups?

**Number of Children in each AGE group (1)**	
**0 to 6 Months** (CntChld_AGE_6m)	
**7 to 12 Months** (CntChld_AGE_8m)	
**13 to 23 Months** (CntChld_AGE_12m)	
**2.0 to 2.9 years** (CntChld_Age_2yr)	
**3.0 to 3.9 years** (CntChld_Age_3yr)	
**4.0 to 4.9 years** (CntChld_Age_4yr)	
**5.0 to 5.9 years** (CntChld_Age_5yr)	
**6.0 years or older** (CntChld_Age_6yr)	
Total	

CntChld_Race
Of the children currently enrolled in your child care program, how many are in each of these categories?

**Number of Children in each Race/Ethnicity group** (1)	
**American Indian or Alaska Native** (CntChld_RaceAmI)	
**Asian (including India)** (CntChld_RaceASN)	
**Black or African-American** (CntChld_RaceBLK)	
**Hispanic, Latino, or Spanish origin** (CntChld_RaceHisp)	
**Native Hawaiian or Other Pacific Islander** (CntChld_RaceHaw)	
**White** (CntChld_RaceWhT)	
**Two or More Races** (CntChld_RaceTWO)	
**Race or Ethnicity category not listed** (CntChld_RaceNOT)	
**Unknown Race or Ethnicity** (CntChld_RaceUNkn)	
Total	

CntChld_Subsidy
About what percent of children in your program receive child care subsidies? *(if unsure, provide your best estimate)*

- ○ 0% (No children receive subsidies) (0)
- ○ 10% (10)
- ○ 20% (20)
- ○ 25% (25)
- ○ 30% (30)
- ○ 40% (40)
- ○ 50% (about half of children receive subsidies) (50)
- ○ 60% (60)
- ○ 70% (70)

- ○ 75% (75)
- ○ 80% (80)
- ○ 90% (90)
- ○ 100% (All children receive subsidies) (100)

CntChld_Eng

Do any children in your program speak a language other than English as their primary means of communication?

- ○ YES (1)
- ○ NO (0)

Display this question:
If Do any children in your program speak a language other than English as their primary means of com... = YES

CntChld_Lang

Which languages, other than English, do children use as primary means of communication? *[Mark all that apply]*

- ☐ Spanish (1)
- ☐ American Sign language (hearing impaired) (2)
- ☐ Chinese (Mandarin, Cantonese, Hokkien and other varieties) (3)
- ☐ Tagalog (including Filipino) (4)
- ☐ Vietnamese (5)
- ☐ French (6)
- ☐ Arabic (7)
- ☐ Korean (8)
- ☐ Other (specify) (9) ____

CntDSenroll

Does your center currently have any children who attend regularly with a Down Syndrome or Autism diagnosis?

- ○ YES (1)
- ○ NO (0)

The next few questions are about the staff who work for your child care program.

CntStf_num

Including yourself, about how many people work Full-time or Part-time for this child care program*?* *(i.e Full- or Part-time paid employment on-site)*

- Full-Time employees (30+ hours per week) (4) ________________________________
- Part-Time employees (less than 30 hours per week) (5) ________________________________

CntStf_type

Including yourself, about how many people in each of these groups work for this center? Include people who work onsite either full or part time Count each person in only one category Do not include people who work offsite or for other organizations that serve the center (e.g. deliver food, state consultant)

Number of Employees in this group (1)	
**Directors and Assistant Directors** (CntStf_DirNum)	
**Teachers (lead or assistant)** (CntStf_TchNum)	
**Class Aides or Floaters** (CntStf_AidNum)	
**Food/Kitchen Staff** *(not already counted)* (CntStf_FoodNum)	
**Administrative/Office Staff** *(not already counted)* (CntStf_AdminNum)	
Total	

CntStf_Eng

Are any current staff members fluent in a language other than English? *(e.g. Spanish, American Sign Language, Chinese, French)*

- ○ YES (1)
- ○ NO (0)

Display this question:
If Are any current staff members fluent in a language other than English? (e.g. Spanish, American Si... = YES

CntStf_Lang

Which language(s), other than English, can one or more staff members speak fluently? *[Mark all that apply]*

- ☐ Spanish (1)
- ☐ American Sign language (hearing impaired) (2)
- ☐ Chinese (Mandarin, Cantonese, Hokkien and other varieties) (3)
- ☐ Tagalog (including Filipino) (4)
- ☐ Vietnamese (5)
- ☐ French (6)
- ☐ Arabic (7)
- ☐ Korean (8)
- ☐ Other (specify) (9) ____

DirStaffLead1

How often do you feel this statement describes the staff at your center? **The staff at our center lead by example, work hard on improvements, and support the adminstrative staff.**

- ○ Never (1)
- ○ Very Rarely (2)
- ○ Rarely (3)
- ○ Occasionally (4)
- ○ Sometimes (5)
- ○ Frequently (6)
- ○ Very Frequently (7)
- ○ Always (8)

CD-RISC

We are administering CDRISC-25. This survey requires small fee for use and cannot be reproduced here. If interested, please contact survey creator at https://www.cd-risc.com/index.php

Physical Activity

As you answer the next few questions think about the exercise and other physical activities you did over the past week (last 7 days). We will ask you to report activities in two broad categories:

- Vigorous intensity - you are breathing hard and fast, and your heart rate has gone up quite a bit, like jogging.
- Moderate intensity - you are working hard enough to increase your heart rate and breathing some, like walking.

PAvigIntro

First, we will ask about physically strenuous, or vigorous, physical activity you did outside of work during the last week. Moderate intensity activities will be reported in the next question.

- DO NOT count job-related activities or things done as part of your job at the child care program.
- Vigorous activities feel as intense as jogging. Usually, you are breathing hard and fast, and your heart rate has gone up quite a bit.
  - *Examples might be: Zumba, jogging, high intensity exercise class, HITT workout, fast biking, swimming laps, basketball, soccer, jumping rope, heavy weight lifting.*

PAvigDays

Outside of job-related work, **How many days last week did you participate in strenuous, or vigorous intensity, physical activity?**

- 0 days (0) to 7 days (7)

Display this question:
If Outside of job-related work, How many days last week did you participate in strenuous, or vigorou... != 0 days

PAvigMinPerDay

On the ${PAvigDays/ChoiceGroup/SelectedChoices} days you did vigorous activity last week, about how many minutes per day did you do? *if the amount of time each day differs, try to report average day.*

- Less than 5 min per day (3)
- 5 minutes per day (5)
- 10 minutes per day (10)
- 15 minutes per day (15)
- 20 minutes per day (20)
- 30 minutes per day (30)
- 45 minutes per day (45)
- 60 minutes per day (60)
- 75 minutes per day (75)
- 90 minutes per day (90)
- 120 minutes (2 hours) per day or more (120)

PAmodIntro

Now, we will ask about the moderate intensity physical activity you did outside of work during the last week.

- DO NOT count job-related activities or things done as part of your job at the child care program.
- Moderate Activities feel as intense as walking at a normal pace. When doing them you are working hard enough to increase your heart rate and breathing.
  - *Examples might be: Walking briskly, water aerobics, biking slower than 10 MPH, pushing lawn mower, softball, general gardening, light weight lifting.*

PAmodDays

Outside of job-related work, **How many days last week did you participate in moderate intensity physical activity?**

- 0 days (0) to 7 days (7)

Display this question:
If Outside of job-related work, How many days last week did you participate in moderate intensity ph... != 0 days

PAmodMinPerDay

On the ${PAmodDays/ChoiceGroup/SelectedChoices} days you did moderate intensity physical activity last week,

about how many minutes per day did you do? *if the amount of time each day differs, try to report average day.*

- ○ Less than 5 min per day (3)
- ○ 5 minutes per day (5)
- ○ 10 minutes per day (10)
- ○ 15 minutes per day (15)
- ○ 20 minutes per day (20)
- ○ 30 minutes per day (30)
- ○ 45 minutes per day (45)
- ○ 60 minutes per day (60)
- ○ 75 minutes per day (75)
- ○ 90 minutes per day (90)
- ○ 120 minutes (2 hours) per day or more (120)

PAstrIntro

Next, we will ask about muscle strengthening exercise.

- You likely included this type of exercise in your moderate or vigorous activity already reported.
- *Muscle strengthening activities include things like lifting weights, using resistance bands, or doing exercises that use your body weight for resistance (example: push-ups, sit-ups, Yoga, etc.)*

PAstrDays

Outside of job-related work, **How many days last week did you do exercise that included muscle strengthening activities?**

- ○ 0 days (0) to 7 days (7)

Display this question:

If Outside of job-related work, How many days last week did you do exercise that included muscle str... != 0 days

STR_type

What type of muscle strength activities did you do last week? *[Mark all that apply]*

- ☐ ⊗NO strength training activities this week (6)
- ☐ Lifting Weights (free weights or machines) (1)
- ☐ Workout with Resistance Bands (2)
- ☐ Body weight strength exercises (push-ups, pull-ups, burpee) (3)
- ☐ Class/Group Workout with Muscle Strength Focus (like Muscle Pump, HiiT) (4)
- ☐ Other (5) ____

PA_rating

How would you describe your level of physical activity and exercise last week?

- ○ A lot less Active than normal (1)
- ○ Less Active than normal (2)
- ○ Activity level same as usual this week (3)
- ○ More Active than normal (4)
- ○ A lot More Active than normal (5)

Readiness for Action

The next few items ask about things people sometimes do, or change. For each, think about how likely you are to do the behavior over the next 3 months.

	Over the next 3 months, how likely are you to...	*I will Not do (1)*	*Very Unlikely (2)*	*Unlikely (3)*	*Somewhat Unlikely (4)*	*Somewhat Likely (5)*	*Likely (6)*	*Very Likely (7)*
DirRdy13	...respond by text message to a question about your daily habits?	○	○	○	○	○	○	○
DirRdy06	...visit a website weekly to look through lessons, information, and resources, related to your health and wellness goals?	○	○	○	○	○	○	○
DirRdy32	...attend a one-on-one coaching call focused on leadership strategies that promote staff health and wellness every 3 weeks?	○	○	○	○	○	○	○
DirRdy21	...encourage coworkers to try the things you have learned in a health and wellness program?	○	○	○	○	○	○	○
DirRdy22	...read a short chapter in a book each week with information related to a health and wellness program you are attending?	○	○	○	○	○	○	○
DirRdy23	...track your daily health habits? *(like stress management, sleep, meditation, or physical activity)*	○	○	○	○	○	○	○
DirRdy24	...use a journal each day to reflect on your personal growth and wellness journey?	○	○	○	○	○	○	○
DirRdy33	...help connect staff members who may be interested in the same health activities? (e.g. walking, yoga, meditation, cooking)	○	○	○	○	○	○	○
TchRdy25	...decrease prolonged sitting each day? *(sitting more than 30 minutes without moving around)*	○	○	○	○	○	○	○
DirRdy34	...give staff 10 minutes of personal time each workday for health activities? (e.g. walk, quiet reflection, stretching, yoga, meditation)	○	○	○	○	○	○	○
DirRdy18	...add more exercise or physical activity to your weekly routine?	○	○	○	○	○	○	○
DirRdy35	...gather feedback and concerns from staff at your center each week?	○	○	○	○	○	○	○
DirRdy1	...exercise 150 or more minutes per week? *(about 22 minutes per day)*	○	○	○	○	○	○	○
DirRdy36	...organize a way for staff to provide gratitude or positive feedback for coworkers?	○	○	○	○	○	○	○
DirRdy2	...wear a fitness/activity tracker to help monitor your exercise? *(like a Fitbit, Amazfit band, or smart watch)*	○	○	○	○	○	○	○
DirRdy37	...individually thank or encourage (verbal or written) most staff each week?	○	○	○	○	○	○	○
DirRdy28	...practice personal mindfulness through meditation, gratitude practice, and self-compassion for at least 5 minutes each day?	○	○	○	○	○	○	○
DirRdy38	...suggest group activities for center staff to do together outside of work? (e.g. take their kids to a park, meal together, Zumba class)	○	○	○	○	○	○	○
DirRdy30	...add new mindfulness and calming techniques to your weekly routine?	○	○	○	○	○	○	○
DirRdy31	...spend 2 minutes before getting out of bed each morning to think of 5 people you are grateful for and why?	○	○	○	○	○	○	○
DirRdy39	...include educational materials or learning opportunities related to health and wellness behaviors during each staff meeting?	○	○	○	○	○	○	○

Worksite Wellness

The next set of questions are about your center's current efforts to promote health, wellness, and safety among your staff. As you answer questions, please focus on what your center has been doing during the past 6 months.

CNTwell_stfint

In your opinion, how interested do you think your staff are in participating in health and wellness programs offered through the center?

- *Examples: Diabetes prevention, Back health, Weight loss, Healthy eating, Physical activity, Stress reduction Try to rate their overall interest on a scale of 1 to 10, where 1 is not at all interested and 10 is extremely interested.*

- ○ 1 Not at all interested (1)
- ○ 2 (2)
- ○ 3 (3)
- ○ 4 (4)
- ○ 5 (5)
- ○ 6 (6)
- ○ 7 (7)
- ○ 8 (8)
- ○ 9 (9)
- ○ 10 Extremely Interested (10)

CNTwell_offerimp

How important do you think it is for your center to offer health and wellness programs for staff? *Examples: Diabetes prevention, Back health, Weight loss, Healthy eating, Physical activity, Stress reduction*

- ○ 1 Not at all important (1)
- ○ 2 (2)
- ○ 3 (3)
- ○ 4 (4)
- ○ 5 (5)
- ○ 6 (6)
- ○ 7 (7)
- ○ 8 (8)
- ○ 9 (9)
- ○ 10 Extremely Important (10)

CNTwell_goals

Does your center have goals, written policies, or action plans for staff wellness programs and activities?

- ○ YES (1)
- ○ NO (0)

CNTwell_offerYN

During the past 6 months, did your center offer or promote any staff wellness, health, or safety programs, educational materials, or trainings?

- This includes programs, educational materials, and trainings offered by your center, a local community organization, insurance company, or other group.
  - ○ *Programs include things like group meetings, classes, or activities.*
  - ○ *Educational materials can be print/paper or electronic and offer helpful information about a health or safety-related topic or issue.*
  - ○ *Trainings include activities that might be required to work, issue continuing education credits, or result in a certification/certificate*

- ○ YES (1)
- ○ NO (0)

Skip To: CNTwell_CommHealthYN If During the past 6 months, did your center offer or promote any staff wellness, health, or safety... = NO

Display this question:
If During the past 6 months, did your center offer or promote any staff wellness, health, or safety... = YES

CNTwell_Topics
During the past 6-months, **Were any of these topics covered in the staff wellness, health, or safety programs, materials, or trainings?** *[Mark all topics covered]*

- ☐ Physical activity and exercise (1)
- ☐ Personal nutrition (2)
- ☐ Weight management (3)
- ☐ Stress management (4)
- ☐ Sleep health (5)
- ☐ Meditation/Relaxation (6)
- ☐ Smoking cessation (7)
- ☐ Breathing/calming techniques (8)
- ☐ Resilience (9)
- ☐ Flu, cold, illness prevention (10)
- ☐ Injury prevention at work (11)
- ☐ Reducing exposure to work place hazards (12)
- ☐ Reducing work stress (13)
- ☐ Staff communication or conflict resolution (14)
- ☐ Back health (15)
- ☐ Strength Training (16)

CNTwell_ComOrg
During the past 6 months, **Which of these community organizations has your center worked with in connection to staff wellness programs or activities offered, or promoted, at your center?** *Mark all that apply*

- ☐ State or County Health Department (1)
- ☐ Health Insurance Provider (2)
- ☐ Insurance Company (3)
- ☐ Health Related Organization (American Heart Assoc, American Cancer Society) (4)
- ☐ Local Hospital (5)
- ☐ YMCA or YWCA (6)
- ☐ Town, city, or county government organization (Chamber of commerce, town wellness council) (7)
- ☐ Parks and Recreation Department (local or state) (8)
- ☐ Local Gym or Fitness center (9)
- ☐ Community College or Local University (10)
- ☐ Other Community Group (11)
- ☐ ⊗We have NOT worked with any of these organizations (12)

CNTwell_who
During the past 6-months, **Who is mainly responsible for planning, finding, and getting word to staff about wellness and health initiatives/programs?**

- ○ Individual: Administrative staff (Director, Assistant Director, HR, Office Manager) (1)
- ○ Individual: Teacher or other staff (2)
- ○ Small group of administrative staff (3)
- ○ Small group of teachers/staff (4)
- ○ Group, or committee, including teachers and administrative staff (5)
- ○ No one at our center does this (6)

CNTwell_budgetYN

Does your center have a budget for staff wellness, health, and safety programs and activities?

- YES (1)
- NO (0)

Display this question:
If Does your center have a budget for staff wellness, health, and safety programs and activities? = YES

CNTwell_budgetAMT
About how much is budgeted this year for staff wellness, health, and safety programs and activities?

- Less than $100 (1)
- $100 to $250 (2)
- $250 to $500 (3)
- $500 to $750 (4)
- $750 to $1000 (5)
- $1000 to $1500 (6)
- $1500 to $2000 (7)
- $2000 to $2500 (8)
- $2500 to $5000 (9)
- $5000 or more per year (10)
- Not sure (11)

CNTwell_inform
What are the top ways, staff are informed about staff wellness, health, and safety programs and activities being offered?

Select 1 to 4 methods most often used

- ☐ Employee Orientation (1)
- ☐ During Staff Meetings (2)
- ☐ Texts (3)
- ☐ Emails (4)
- ☐ Flyers or Brochures (5)
- ☐ Bulletin Board or Resource Table in Staff Area (6)
- ☐ Newsletter (7)
- ☐ Personal Conversations (8)
- ☐ Word of Mouth (9)
- ☐ Insurance Company (10)
- ☐ Other (specify) (11) ____

CNTwell_CommHealthYN
During the past 6 months, **Did your center or staff work with other businesses or community organizations on improving the health and wellness of the city, town, county, or community where your center is located?**

- YES (1)
- NO (0)

CNTwell_Barrier
Thinking about all the health and wellness programs and activities your center offers, or would like to offer, what are the 3 or 4 biggest barriers or challenges to your center's success?

- ☐ Lack of staff interest (1)
- ☐ Staff distrust of employer-sponsored programs (2)
- ☐ Staff not able to participate due to health risks (3)
- ☐ Lack of funding (4)
- ☐ Lack of resources (5)
- ☐ Conflict with other center business demands are too much (6)
- ☐ Confidentiality (7)
- ☐ Lack of knowledge (8)

- ☐ Lack of support from management (9)
- ☐ Legal issues such as liability (10)
- ☐ Lack of person/people to lead efforts (11)
- ☐ Other (specify) (12) ____

CNTwell_BenfA

During the past 6 months, did your center offer any of the following for center staff? Our center was able to provide...

	**YES** (1)	**NO** (0)
...a mentor for new teachers (CNTbenefit01)	○	○
...behavior specialist for classroom help (CNTbenefit02)	○	○
...reduced/free tuition for staff member's child (CNTbenefit03)	○	○
...on-site gym or space for working out (CNTbenefit04)	○	○
...free or low cost membership to local gym/fitness center (CNTbenefit05)	○	○
...free or low cost mental health support (CNTbenefit06)	○	○
...space at center for quiet relaxation that staff can use (CNTbenefit07)	○	○
...health risk appraisals (CNTbenefit08)	○	○
...health assessments or screenings (CNTbenefit09)	○	○
...free meal(s) and snack when working (CNTbenefit10)	○	○
...common space separate from children for socializing or group activities. (CNTbenefit11)	○	○
...training for new teachers and staff (CNTbenefit12)	○	○

CNTwell_BenfB

During the past 6 months, did your center offer any of the following for center staff? Our center was able to provide...

	**YES** (1)	**NO** (0)
...health insurance (CNTbenefit13)	○	○
...funding assistance for educational costs (CNTbenefit14)	○	○
...retirement program such as 401K (CNTbenefit15)	○	○
...employer contributions to retirement savings (CNTbenefit16)	○	○
...paid maternity leave (CNTbenefit17)	○	○
...paid sick leave (CNTbenefit18)	○	○
...paid vacation days (CNTbenefit19)	○	○
...paid time-off for job-related training or classes (CNTbenefit20)	○	○
...ability to take unpaid leave (CNTbenefit21)	○	○
...help with transportation to and from work (CNTbenefit22)	○	○

CntWell_Champ

Do you feel like your center has an employee wellness champion, or network of champions, who actively publicize and promote health and wellness programs? *Wellness Champions are health-minded employees who are passionate about creating a healthier workplace. They serve as positive role models, provide peer support, and offer feedback to leadership to improve wellness offerings.*

- ○ YES (1)
- ○ NO (0)

The questions in this section ask how you feel about different aspects of your job at this child care center. If you have more than one job, please answer questions as they apply to your job at the center involved in the OnWARD program.

WellBq01

Which response completes this statement best for you today. **Overall, I am ______ with my job.**

- not at all satisfied (1)
- not too satified (2)
- somewhat satisfied (3)
- very satisfied (4)

WellBq13a

How often do you experience physical fatigue when you are working?

- Never (1)
- Almost Never *(a few times a year or less)* (2)
- Rarely *(once a month or less)* (3)
- Sometimes *(a few times a month)* (4)
- Often *(once a week)* (5)
- Very Often *(a few times a week)* (6)
- Always *(every day)* (7)

WellBq13b

How often do you experience mental fatigue when you are working?

- Never (1)
- Almost Never *(a few times a year or less)* (2)
- Rarely *(once a month or less)* (3)
- Sometimes *(a few times a month)* (4)
- Often *(once a week)* (5)
- Very Often *(a few times a week)* (6)
- Always *(every day)* (7)

WellBq14

My work inspires me.

- Never (1)
- Almost Never *(a few times a year or less)* (2)
- Rarely *(once a month or less)* (3)
- Sometimes *(a few times a month)* (4)
- Often *(once a week)* (5)
- Very Often *(a few times a week)* (6)
- Always *(every day)* (7)

WellBq15

I am immersed in my work.

- Never (1)
- Almost Never *(a few times a year or less)* (2)
- Rarely *(once a month or less)* (3)
- Sometimes *(a few times a month)* (4)
- Often *(once a week)* (5)
- Very Often *(a few times a week)* (6)
- Always *(every day)* (7)

WellBq16

When I get up in the morning, I feel like going to work.

- Never (1)
- Almost Never *(a few times a year or less)* (2)
- Rarely *(once a month or less)* (3)

- Sometimes *(a few times a month)* (4)
- Often *(once a week)* (5)
- Very Often *(a few times a week)* (6)
- Always *(every day)* (7)

WellBq27

How often do the demands of your job interfere with your personal life?

- Never (1)
- Almost Never *(a few times a year or less)* (2)
- Rarely *(once a month or less)* (3)
- Sometimes *(a few times a month)* (4)
- Often *(once a week)* (5)
- Very Often *(a few times a week)* (6)
- Always *(every day)* (7)

WellBq28

How often do the demands of your personal life interfere with your work at this center?

- Never (1)
- Almost Never *(a few times a year or less)* (2)
- Rarely *(once a month or less)* (3)
- Sometimes *(a few times a month)* (4)
- Often *(once a week)* (5)
- Very Often *(a few times a week)* (6)
- Always *(every day)* (7)

WellBqintro02

How much do you agree or disagree with each statement about your job at this center.

How much do you agree or disagree with each statement?

	How much do you agree or disagree with each statement about your job at this center.	*Strongly Disagree (1)*	*Disagree (2)*	*Slightly Disagree (3)*	*Slightly Agree (4)*	*Agree (5)*	*Strongly Agree (6)*
WellBq05	I can count on my supervisor for support when I need it.						
WellBq06	I can count on my coworkers for support when I need it.						
WellBq07	I feel my job is secure.						
WellBq08	I am given a lot of freedom to decide how to do my own work.						
WellBq09	I never seem to have enough time to get everything done on my job.						
WellBq10	The work I do is meaningful to me.						
WellBq11	The work I do serves a greater purpose.						
WellBQ17	At my center, I am treated with respect.						
WellBQ18	My center values my contributions.						
WellBq19	My organization cares about my general satisfaction at work.						
WellBQ20	My organization is willing to extend resources in order to help me perform my job to the best of my ability.						
WellBQ21	I receive recognition for a job well done.						
WellBQ22	I trust the management at my organization.						
WellBQ23	The center where I work is committed to employee health and well-being.						
WellBq24	The center where I work encourages me and provides opportunities to engage in healthy behaviors, such as being physically active, eating a healthy diet, living tobacco free, and managing my stress.						

WellBqe06	I feel motivated in my work.						
WellBqe07	I take pride in my work						
WellBqE11	I think of the people I work with as friends.						
WellBqE12	The people I work with support my health and wellness efforts.						
WellBqE08	My coworkers and I are happy to work alongside each other.						
WellBqE09	My coworkers and I understand and respect each other.						
WellBqE10	My coworkers and I communicate job information to each other effectively.						

Absenteeism Turnover

The next set of questions ask about hiring, turnover, and staff retention at your center. Some questions are about staff in general, some focus on teachers and other classroom staff only.

CntHire_Full

Do you currently have enough staff to operate at full capacity?

- Yes (1)
- No (0)

DirRoleHire

Which of these best describes your role in the hiring process at this center?

- I do all the hiring with no help (1)
- I do all the hiring, with a little help (2)
- I am involved in hiring, but other staff handle a lot of the work (3)
- I am not involved in hiring (4)
- Other (please describe) (5) ____

CntHire_open

How many teacher, or classroom, positions are you currently trying to fill? *could be hiring for lead teachers, assitant teachers, or other staff that work in classrooms regularly*

- 0 (we do not have any open teacher positions at this time) (0)
- (1)
- (2)
- (3)
- (4)
- (5)
- or more (6)

CntHire_Filltm

About how long does it take to fill an open teacher position at your center?

- Few days (0.5)
- 1 week (1)
- 2 weeks (2)
- 3 weeks (3)
- 4 weeks (1 month) (4)
- 5-7 weeks (6)
- 2 months (8)
- 3 months (12)
- 4-5 months (16)
- 6 months or more (24)

CntHire_hireNum

Over the last 6 months how many staff have been hired at your center in each of these categories.
How many staff has your center hired to...

- **...fill a new position** (newly created or re-opened after long period) : _______ (1)
- **...fill an open position after someone __changed jobs within your center__** include people who took a job at a center with the same owner, or in same chain *e.g. assistant teacher moved into lead teacher role and needed new assistant teacher* : _______ (2)
- **...fill an open position __after someone left center__?** : _______ (3)

CntHire_TurnType
With this question we are trying to categorize two types of turnover centers often have. **Of the people who left a job at your center in the past 6 months, About how many...** *If no one has left a job at your center in the past 6 months enter 0 for both types*

- **...left or resigned by choice?** *(e.g. retired, found a different job, decided not to work)* (1) __________
- **...were dismissed or fired?** *(e.g. could not do job, missing work, center cut their position)* (2) ________

CntHire_Rate
How much do you agree or disagree with these statements about hiring and turnover.

	Strongly Disagree (1)	*Disagree (2)*	*Slightly Disagree (3)*	*Slightly Agree (4)*	*Agree (5)*	*Strongly Agree (6)*
**Retaining quality staff at our center is an issue.** (CntHire_Rate01)	○	○	○	○	○	○
**Finding quality staff is hard.** (CntHire_Rate02)	○	○	○	○	○	○
**Staff turnover makes improving our center difficult.** (CntHire_Rate03)	○	○	○	○	○	○
**The time it takes to hire and train new staff makes it hard to keep up with other tasks.** (CntHire_Rate04)	○	○	○	○	○	○
**Staff missing work is an issue at our center.** (CntHire_Rate05)	○	○	○	○	○	○

CntHire_MissWork
This question is asking about teachers, or classroom staff, missing work for both planned and unplanned reasons.

- *planned - Administrative staff were aware of, granted permission, and had time to prepare for the absence*
- *unplanned - Administrative staff were NOT aware, absence was unexpected and not planned for*
- We ask about three types of missed work time: missing full shift, partial shift, and being late.

In the past 4 weeks (about 28 days), how many days did at least 1 teacher, or classroom staff member...

	**Planned / Let center know ahead of time** *(# days you missed work)*	**Unplanned / NOT expected** *(# days you missed work)*
**...missed an entire shift, or workday.** (CntHire_MissFull)	*Drop down 0 to 28 days*	*Drop down 0 to 28 days*
**...missed part of a shift, or part of workday** *(arrive late or leave early by more than 60 minutes)* (CntHire_MissPart)	*Drop down 0 to 28 days*	*Drop down 0 to 28 days*
**...miss 15 to 60 minute because they were late to work.** (CntHire_MissLate)	*Drop down 0 to 28 days*	*Drop down 0 to 28 days*

CntHire_extraShift
In the past 4 weeks (about 28 days), **How many days did you have to ask a staff member to work extra on short notice because another staff member did not come to work or left work early?** *This would include asking someone to come in early, leave later than scheduled, or work on a scheduled day-off.*

- DROPDOWN: 0 days to 28 days
